# RXRα suppression drives hepatic metabolic and immune dysfunction in sepsis

**DOI:** 10.1038/s44321-026-00480-y

**Published:** 2026-07-16

**Authors:** Matyas Jelinek, Jolien Vandewalle, Marah Heyerick, Tineke Vanderhaeghen, Céline Van Dender, Steven Timmermans, Madeleine Hellemans, Daria Fijalkowska, Hilde De Rooster, Martin Guilliams, Karolien De Bosscher, Claude Libert

**Affiliations:** 1 VIB Center for Inflammation Research, VIB, Ghent, Belgium; 2 Department of Biomedical Molecular Biology, Ghent University, Ghent, Belgium; 3 VIB Center for Medical Biotechnology, VIB, Ghent, Belgium; 4 Department of Biomolecular Medicine, Ghent University, Ghent, Belgium; 5 Small Animal Department, Faculty of Veterinary Medicine, Ghent University, Merelbeke, Belgium

**Keywords:** Immunology, Microbiology, Virology & Host Pathogen Interaction

## Abstract

Sepsis is a life-threatening condition in which a dysregulated host response to infection leads to organ dysfunction and metabolic and immune failure. We identify hepatocyte retinoid X receptor α (RXRα) as a key integrator of host resilience during polymicrobial sepsis. RXRα is transcriptionally regulated by hepatocyte nuclear factor 4α (HNF4α), and sepsis rapidly decreases RXRα mRNA and protein levels. Transcriptomic analyses show that the septic liver becomes partially resistant to pharmacological activation of RXRα with bexarotene. Prophylactic- but not therapeutic- bexarotene improves survival by preserving metabolic stability and enhancing bacterial clearance. In hepatocyte-specific inducible RXRα-deficient mice, this protection is lost, confirming dependence on hepatocyte RXRα. Loss of RXRα in hepatocytes reduces Kupffer cell numbers, resulting in bacterial dissemination and mortality, a phenotype reproduced in a genetic model of selective Kupffer cell ablation. Overall, RXRα maintains the hepatic macrophage niche, linking hepatocellular transcriptional competence to systemic antibacterial defense.

## Synopsis

Sepsis triggers severe hepatic metabolic and immune collapse, but the underlying mechanisms remains elusive. This study demonstrates that peritoneal sepsis dismantles the hepatic HNF4α-RXRα axis, which is shown to be crucial for preserving the Kupffer cell niche and systemic host defense.

- Hepatic Retinoid X Receptor α (RXRα) is shown to be transcriptionally dependent on Hepatocyte Nuclear Factor α (HNF4α) and is rapidly suppressed at mRNA and protein levels early after sepsis insult (cecal ligation and puncture model, CLP).
- Host survival and metabolic stability are improved by prophylactic, but not therapeutic, pan-RXR agonist administration (Bexarotene) through the stimulation of Kupffer cell mediated phagocytosis causing enhanced multi-organ bacterial clearance.
- A state of partial RXR resistance is induced upon CLP, whereby the liver transcriptomic response to therapeutic Bexarotene is reduced.
- Mice with hepatocyte-specific RXRα deficiency are presented with reduced Kupffer cell abundance, increased bacterial burden and complete mortality following CLP; effects that are potentially mediated through suppressed expression of local niche-supportive factors in these mice.
- The sepsis induced suppression of the hepatic RXR regulatory network is validated as a highly conserved cross-species feature within a clinical cohort of dogs with naturally acquired peritonitis.

## Abstract (web summary)

Sepsis triggers severe hepatic metabolic and immune collapse, but the underlying mechanisms remains elusive. This study demonstrates that peritoneal sepsis dismantles the hepatic HNF4α-RXRα axis, which is shown to be crucial for preserving the Kupffer cell niche and systemic host defense.

## Graphical abstract

**Figure:**
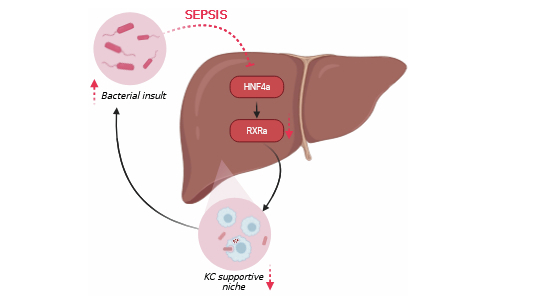

**Figure:**
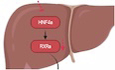

The paper explained **Problem**

Sepsis is a life-threatening medical emergency driven by a dysregulated host response to infection that causes devastating systemic organ dysfunction and a collapse of core immune and metabolic processes. The liver operates as a crucial metabolic hub coordinating energy homeostasis, and its functional failure is a main driver of sepsis progression and mortality. While nuclear receptors are known to integrate metabolic and inflammatory signals, making them highly attractive drug targets, the specific role of hepatic retinoid X receptor α (RXRα) in coordinating host resilience during polymicrobial sepsis has been unknown.

**Results**

Using experimental mouse models and clinical datasets from dogs, this study identifies hepatocyte RXRα as a conserved, central coordinator of metabolic stability and systemic antibacterial defense during sepsis. The researchers discovered that hepatic RXRα expression is downstream of the master regulator HNF4α and is rapidly suppressed at both the mRNA and protein levels during early sepsis. This downregulation induces a state of partial RXR resistance, rendering the liver highly insensitive to pharmacological stimulation by the RXR agonist Bexarotene once systemic inflammation is established. Consequently, prophylactic, but not therapeutic, Bexarotene treatment preserves body temperature, blood glucose levels, and local bacterial control, thereby significantly improving survival. Crucially, genetic deletion of RXRα specifically in hepatocytes suppresses vital microenvironmental niche factors, causing a profound physical depletion of Kupffer cells. This cellular loss causes progressive bacterial accumulation, systemic dissemination, and metabolic collapse, a lethal phenotype that is recapitulated in a genetic model of selective Kupffer cell ablation.

**Impact**

These findings establish a critical non-cell-autonomous mechanism where hepatocellular transcriptional competence directly sustains local immune cell survival and systemic pathogen clearance. By defining RXRα as an essential “immunometabolic hub,” this work demonstrates that protective interventions targeting this pathway must be deployed early in the course of infection before transcriptional resistance sets in. Because standard therapeutic administration of RXR agonists alone is ineffective once the pathway is blunted, future clinical strategies will likely depend on uncovering interventions that reverse the upstream collapse of HNF4α. Successfully stabilizing this upstream regulatory cascade could restore the wider RXR heterodimer network, opening a novel therapeutic window to preserve hepatic immunometabolic function and improve survival outcomes in septic patients.

## Introduction

Sepsis is a life-threatening condition characterized by organ dysfunction resulting from a dysregulated host response to infection, posing a major global health burden with high morbidity and mortality (Singer et al, 2016). In 2017, the World Health Organization recognized sepsis as a global health priority, emphasizing it as one of the most urgent unmet medical challenges of our times (Reinhart et al, 2017). Recent estimates indicate that the global impact is even more severe than previously reported; in 2021 alone, sepsis affected ~166 million individuals worldwide and was responsible for 21.4 million deaths, accounting for roughly one-third of all global mortality (Gray et al, 2025; La Via et al, 2025; Mou et al, 2026). Its complexity stems from the intricate interactions between invading pathogens and the host response, highlighting the need to better understand the molecular mechanisms underlying sepsis to develop effective therapeutic interventions (Delano and Ward, 2016; Jarczak et al, 2021). Current clinical management remains largely supportive, focusing on antibiotics, hemodynamic stabilization, and organ support, rather than curative (Van Wyngene et al, 2018; Tang et al, 2025).

The early immune response to infection simultaneously activates pro- and anti-inflammatory pathways (Cajander et al, 2024). Pro-inflammatory cytokines, including TNF-α, IL-1β, and IL-6, promote pathogen clearance but can also drive tissue damage and shock, whereas anti-inflammatory mediators such as IL-10 and regulatory immune cells attempt to restore homeostasis, sometimes resulting in immune suppression if prolonged. Alongside these immune responses, sepsis profoundly disrupts core metabolic processes across multiple organs, affecting glycolysis, fatty acid oxidation, and mitochondrial function (Nuyttens et al, 2025). The high energetic demands of an activated immune system, compounded by reduced nutrient intake, create a negative energy balance and induce a starvation-like state similar to prolonged fasting. However, unlike the physiological starvation response, this adaptive response fails in sepsis, thereby complicating energy homeostasis (Van Wyngene et al, 2018; Vandewalle and Libert, 2022). Organ-specific metabolic reprogramming further underscores the systemic impact: the liver exhibits an accumulation of lipid droplets due to reduced β-oxidation, skeletal muscle undergoes catabolism to supply amino acids, which, despite their availability, often fail to be utilized effectively for gluconeogenesis, and mitochondria display impaired oxidative phosphorylation and biogenesis (Van Wyngene et al, 2018; Nuyttens et al, 2025). As the liver acts as the metabolic hub coordinating these processes (including gluconeogenesis and lipid metabolism), its failure to maintain these pathways is a critical driver of sepsis pathophysiology. Nuclear receptors (NRs) work at the nexus of these adaptive responses; as transcriptional regulators that integrate metabolic and immune signals, they represent promising targets for therapeutic intervention.

NRs are ligand-activated transcription factors that regulate gene expression in response to both endogenous and exogenous signals, modulating metabolism, immunity, and cellular growth (Frigo et al, 2021). Ligand binding induces conformational changes, facilitating interactions with co-regulators and binding to hormone response elements in target gene promoters (Millard et al, 2013; Rastinejad et al, 2013). Clinically, with ~16% of marketed drugs targeting this family, NRs are highly druggable, highlighting their therapeutic potential in diseases involving metabolic and inflammatory dysregulation, such as sepsis (Santos et al, 2017).

Hepatic nuclear factor 4α (HNF4α and NR2A1) is a key NR in the liver that integrates the aforementioned metabolic and inflammatory signals, emerging as a central regulator of metabolism and survival in sepsis. Progressive loss of HNF4α in the septic liver was observed in mouse sepsis models and disrupts the expression of several other NRs, including PPARα, impairing fatty acid metabolism (Van Wyngene et al, 2020; Van Dender et al, 2024). This suppression of hepatic molecular identity, which involves the rapid protein loss of HNF4α and other master nuclear receptors, is actively driven by endoplasmic reticulum stress during acute liver injury and sepsis (Dubois et al, 2020). Furthermore, hepatic PPARα has been shown to be critically required for the host’s protective metabolic adaptation to sepsis, as its deficiency prevents the essential shift from glucose to lipid utilization and increases mortality (Paumelle et al, 2019). This loss has also been observed in translational porcine models, where septic shock led to hepatic HNF4α downregulation alongside metabolic collapse and inflammation (Halimi et al, 2024). Consequences include lipid accumulation, impaired energy production, and altered IL-6-mediated acute phase responses, which compromise liver regeneration and worsen sepsis severity (Van Dender et al, 2024). Pharmacological activation of HNF4α using ligands such as N-trans-caffeoyltyramine (NCT) conferred sepsis protection by enhancing chromatin binding and gene expression (Van Dender et al, 2024). Furthermore, NCT mitigated lipid accumulation, improved hepatic acute phase response, and reduced inflammation and organ dysfunction. HNF4α also regulates other NRs, including RXR, FXR, and LXR, suggesting its dysfunction may drive broad metabolic and transcriptional derangements in sepsis.

Retinoid X Receptors (RXRs), RXRα (NR2B1, 53 kDa), RXRβ (NR2B2, 57 kDa) and RXRγ (NR2B3, 51 kDa) are key NRs that coordinate metabolism, inflammation, and development. RXRα is highly expressed in the liver, where it heterodimerizes with other NRs (e.g., PPARs, FXR, LXR) to integrate signaling pathways that are crucial for sepsis survival, such as bile acid metabolism and xenobiotic clearance. Consistent with this, Recknagel et al demonstrated that, compared to predicted survivors, rats predicted to die from sepsis exhibited pronounced hepatic accumulation of bilirubin and bile acids, and impaired xenobiotic excretion (Recknagel et al, 2012). Importantly, pathway analysis of liver transcripts from these animals identified FXR/RXR and PXR/RXR signaling among the top downregulated pathways, suggesting an association between RXRα dysfunction and sepsis-induced hepatic failure (Recknagel et al, 2012). Similarly, in human sepsis patients, elevated plasma bile acids predicted mortality, further supporting the clinical relevance of RXRα-regulated pathways (Recknagel et al, 2012).

Therapeutically administered pan-RXR agonists such as Bexarotene (Bex, Targretin) improved heterodimer formation (with PPARα, LXR, etc.) and reduced inflammatory mediators in LPS-induced endotoxemia (a model of systemic inflammation) (Tunctan et al, 2018). On the other hand, myeloid-specific RXRα deficiency decreased chemokine expression, limiting leukocyte recruitment and attenuating systemic inflammation (Núñez et al, 2010). Furthermore, in acute kidney injury, which resembles some features of sepsis, Bex treatment demonstrated complete protection when given pre- and post-injury (Cao et al, 2022). Furthermore, in recent large-scale proteomics analyses of plasma, the downregulation of pathways involved in LXR/RXR activation was found to be associated with an increased incidence of sepsis (Ishigami et al, 2025). These findings collectively suggest that RXRα dysfunction in sepsis is, on the one hand, a consequence of the inflammatory milieu and, on the other hand, a potential driver of organ failure, particularly by disrupting hepatic immunometabolic homeostasis. However, whether RXRα signaling specifically in the liver influences sepsis progression remains unknown. To address this, we investigated hepatic RXR function genome-wide using the cecal ligation and puncture (CLP) mouse model, a gold standard for polymicrobial sepsis that closely recapitulates the human disease trajectory (Rittirsch et al, 2009; Dejager et al, 2011). Additionally, we generated hepatocyte-specific inducible RXRα-deficient mice (RXRα^iAlbKO^) to study the impact of hepatic RXRα loss-of-function on sepsis progression.

## Results

### Hepatic RXRα signaling is suppressed during early sepsis and is a conserved hallmark across species

**Figure 1:**
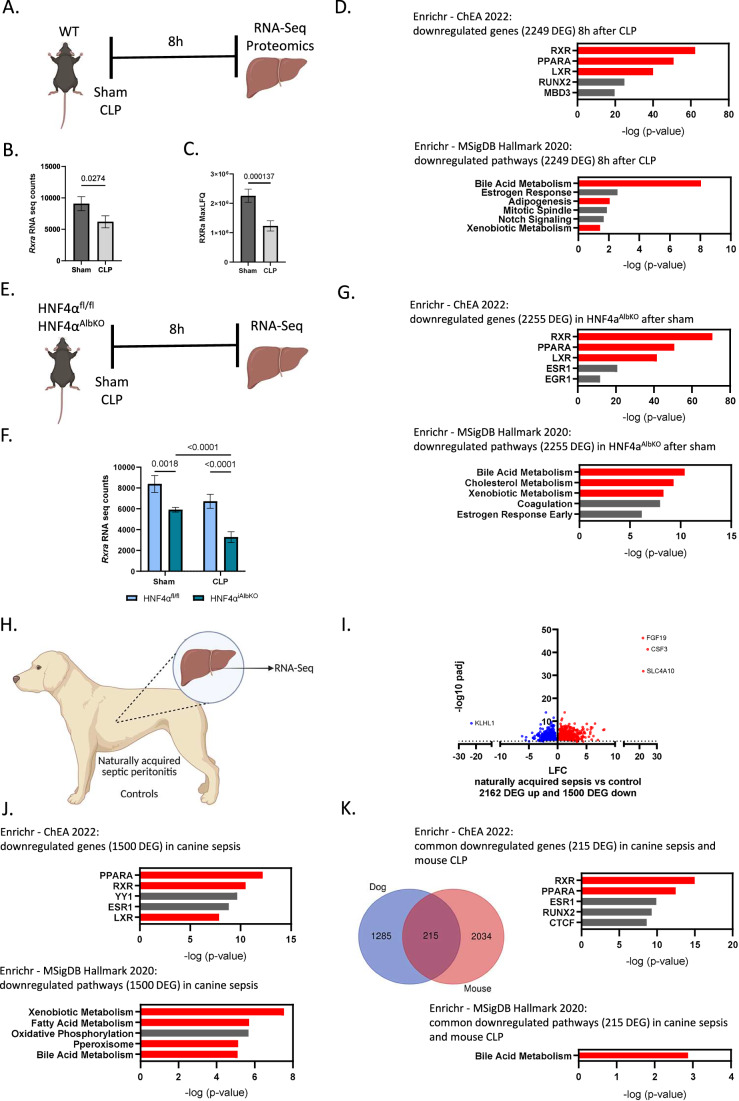
Hepatic RXRα signaling is suppressed during early sepsis and is a conserved hallmark across species. (**A**) Schematic representation of the experimental design for wild-type (WT) mice subjected to sham or CLP surgery. Livers were collected eight h post-surgery for RNA-seq and proteomic analysis. (**B**) Hepatic *Rxra* normalized RNA-seq read counts in WT mice eight h after sham or CLP surgery (*n* = 3 individual mice per group; analyzed by DESeq2 Wald test). (**C**) Hepatic RXRα protein abundance (MaxLFQ intensity) in WT mice eight h after sham or CLP surgery, determined by shotgun proteomics (sham *n* = 7; CLP *n* = 8 individual mice; analyzed by *limma* moderated *t*-test). (**D**) Transcription factor (ChEA 2022, top) and pathway (MSigDB Hallmark 2020, bottom) enrichment analysis of genes significantly downregulated (adj. *p* < 0.05) in WT livers 8 h after CLP compared to sham controls. Pathways and regulators directly associated with the RXRα signaling axis are highlighted in red. (**E**) Schematic representation of the experimental design. Control (HNF4α^fl/fl^) and inducible hepatocyte-specific HNF4α-deficient (HNF4α^iAlbKO^) mice were subjected to sham or CLP surgery, and livers were collected 8-h post-surgery for RNA-seq analysis. (**F**) Hepatic *Rxra* normalized RNA-seq read counts in HNF4α^fl/fl^ and HNF4α^iAlbKO^ mice 8 h after sham or CLP surgery (*n* = 4 individual mice per group; analyzed by DESeq2 Wald test). (**G**) Enrichment analysis of downregulated genes (adj. *p* < 0.05) in HNF4α^iAlbKO^ sham livers compared to HNF4α^fl/fl^. Pathways and regulators directly associated with the RXRα signaling axis are highlighted in red. (**H**) Schematic of the canine study, including non-septic controls (*n* = 9) and dogs with suspected or confirmed complicated intra-abdominal infection (*n* = 14). (**I**) Volcano plot of differentially expressed genes in canine naturally acquired sepsis vs. controls (Blue: downregulated; Red: upregulated; Gray: not significant), identifying 2162 upregulated and 1500 downregulated DEGs. (**J**) Enrichment analysis (ChEA 2022, top; MSigDB Hallmark 2020, bottom) of significantly downregulated genes in canine septic livers. Pathways and regulators directly associated with the RXRα signaling axis are highlighted in red. (**K**) Conservation of the hepatic septic response across species, showing shared downregulated DEGs (215 genes) between mice (eight h CLP) and dogs (naturally acquired septic peritonitis), and enrichment analysis (ChEA 2022, top; MSigDB Hallmark 2020, bottom). Pathways and regulators directly associated with the RXRα signaling axis are highlighted in red. Where applicable, data represent mean ± SD. Exact *p* values are indicated directly within the figure panels. Note: Normalized RNA-seq read counts (panels **B**, **F**) and proteomic abundance (panel **C**) represent distinct biological replicates (*n*) derived from a single dedicated discovery cohort; the complete underlying expression datasets are available via GEO GSE139484, GSE260635, GSE334778, and the manuscript Source Data files. Source data are available online for this figure.

**Figure EV1:**
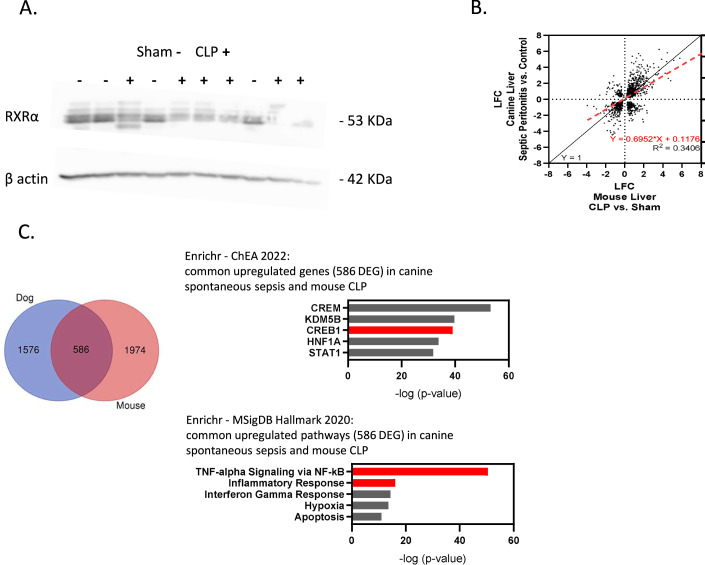
Cross-species transcriptional conservation and hepatic RXRα protein dynamics. (**A**) Western blot analysis of RXRα protein expression in WT liver homogenate 8 h after CLP (*n* = 4 sham; *n* = 6 CLP individual mice). (**B**) Correlation analysis of log_2_ fold-changes (LFC) between spontaneous canine sepsis and murine induced CLP (8 h) evaluated using linear regression and Pearson correlation analysis (y = 0.6952x + 0.1176; *R*^2^ = 0.3406). (**C**) Venn diagram illustrating overlap of upregulated DEGs between septic dogs and mice (586 shared genes), with corresponding enrichment analysis (ChEA 2022, top; MSigDB Hallmark 2020, bottom). Pathways and regulators directly associated with the RXRα signaling axis are highlighted in red.

To characterize hepatic RXRα dynamics during early sepsis, we first analyzed our previously published bulk RNA-sequencing (RNA-seq) datasets (GEO GSE139484) on livers derived from wild-type (WT) mice eight hours after CLP or sham surgery (Fig. 1A); this time point represents an optimal window where septic symptoms are clinically manifest but preceding late-stage decline (Van Wyngene et al, 2020). Hepatic *Rxrα* mRNA levels from RNA-seq were significantly decreased in septic mice compared to sham (Fig. 1B). Furthermore, CLP reduced RXRα protein levels twofold, as shown by shotgun proteomics label-free quantification (LFQ) of RXRα (Fig. 1C) and western blot (Fig. EV1A), confirming that RXRα expression is downregulated at both the mRNA and protein levels during early sepsis. We performed enrichment analysis (using the Enrichr platform) of genes downregulated eight hours after CLP in WT mice (*n* = 3/group; adj *p* < 0.05) to study the signaling pathways negatively affected in the liver after sepsis. Because the upregulated gene signature predominantly reflects the already well-characterized immune and inflammatory responses, we focused our unbiased analysis specifically on the downregulated gene set. In fact, increasing evidence suggests that transcriptionally repressed (or loss-of-function) programs are key drivers of disease severity during sepsis, particularly through disruption of metabolic and homeostatic functions. In agreement with the reduction in RXRα expression, the top five most significantly perturbed regulators included RXR, PPARα, and LXR (Fig. 1D).

Next, using tamoxifen-inducible hepatocyte-specific HNF4α knockout mice (HNF4α^iAlbKO^), we investigated whether sepsis-induced suppression of RXRα is driven by HNF4α. We analyzed our recently published RNA-seq data (GEO GSE260635) from HNF4α^iAlbKO^ and control (HNF4α^fl/fl^) livers collected 8 h after sham or CLP surgery (Fig. 1E). *Rxra* transcript levels were significantly reduced in HNF4α^iAlbKO^ livers under both basal (sham) and septic (CLP) conditions compared to their respective controls (Fig. 1F). Notably, HNF4α^iAlbKO^ mice subjected to CLP exhibited the lowest *Rxra* expression, significantly lower than both HNF4α^iAlbKO^ sham and HNF4α^fl/fl^ CLP mice (Fig. 1F). This indicates that HNF4α is required to maintain basal hepatic *Rxra* expression, and that its genetic loss exacerbates the suppression of RXRα during sepsis. To identify transcriptional regulators and pathways downstream of HNF4α, we performed an unbiased enrichment analysis of genes downregulated in HNF4α^iAlbKO^ mice (HNF4α^iAlbKO^ sham vs HNF4α^fl/fl^ sham). This analysis revealed significant enrichment for RXRα and its heterodimerization partners, including LXR and PPARα, as well as a prominent downregulation of metabolic pathways (bile acid metabolism, cholesterol metabolism, xenobiotic metabolism) (Fig. 1G).

To establish the clinical translatability and generalizability of hepatic HNF4α-linked RXRα dysregulation in sepsis, we compared hepatic transcriptomes from septic dogs with naturally acquired septic peritonitis (*n* = 14) to those of control dogs (*n* = 9) (Fig. 1H). Differential expression analysis (adj *p* < 0.05) of canine septic livers compared to controls revealed broad transcriptomic reprogramming, identifying 2162 upregulated and 1500 downregulated differentially expressed genes (DEGs) (Fig. 1I). Consistent with our murine findings, enrichment analysis of the significantly downregulated genes in septic dogs again identified RXR and its partners, PPARα and LXR, as the top transcriptional regulators (Fig. 1J). Furthermore, metabolic pathways typically controlled by RXR heterodimers, such as xenobiotic and bile acid metabolism, were also significantly suppressed in the canine septic liver (Fig. 1J). These results demonstrate that RXRα dysregulation is a conserved hallmark of both induced and naturally acquired sepsis in mice and dogs, respectively.

To investigate the degree of conservation of this response, we compared the canine sepsis dataset to our murine 8 h CLP model (Fig. 1A–D). DEG correlation analysis of log_2_ fold-changes (LFC) between naturally acquired canine sepsis and induced murine CLP demonstrated a significant conservation of the hepatic septic response (y = 0.6952x ± 0.1176; *R*^2^ = 0.3406) (Fig. EV1B). Furthermore, we analyzed the overlap of downregulated genes between the two species, identifying 215 common downregulated DEGs (Fig. 1K). Enrichment analysis of these shared downregulated genes confirmed that RXR-mediated pathways and its partner, PPARα, represent a highly conserved transcriptional core suppressed during sepsis in both mice and dogs (Fig. 1K). A similar analysis of upregulated DEGs (586 shared genes) revealed a common induction of inflammatory and stress response pathways (Fig. EV1C). Collectively, these data establish that the suppression of RXRα and its associated metabolic regulome, identified here as being under HNF4α control, represents a central, highly conserved feature of the acute hepatic response to sepsis across species.

### Sepsis blunts transcriptional responses to pharmacological RXRα activation

**Figure 2:**
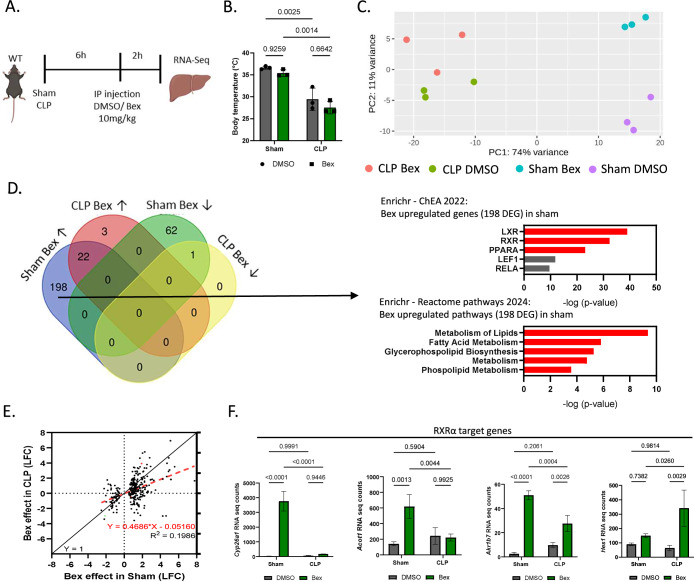
Sepsis blunts transcriptional responses to pharmacological RXRα activation. (**A**) Schematic representation of the experimental design. WT mice were subjected to sham or CLP surgery. Six hours later, mice received an IP injection of Bexarotene (Bex, 10 mg/kg) or vehicle (DMSO). Livers were collected 2-h post-treatment (8 h post-surgery) for RNA-seq analysis. (**B**) Body temperature of mice 8-h post-surgery (*n* = 3 individual mice per group; analyzed by Two-way ANOVA followed by Tukey’s multiple comparisons test). (**C**) Principal component analysis (PCA) of hepatic transcriptomes from Sham and CLP mice treated with DMSO or Bex (*n* = 3/group). (**D**) Venn diagram illustrating the number and overlap of differentially expressed genes (upregulated, left; downregulated, right; adj. *p* < 0.05) in Sham and CLP mice treated with Bex compared to DMSO controls (left). Transcription factor (ChEA 2022) and pathway (Reactome 2024) enrichment analysis of genes significantly upregulated by Bex in Sham mice (right). Pathways and regulators directly associated with the RXRα signaling axis are highlighted in red. (**E**) Scatter plot correlating the LFC of genes significantly upregulated by Bex in sham livers against their LFC in CLP livers. The solid black line represents the unity line (y = x), and the red dashed line represents the linear regression fit (y = 0.4686x ± 0.05160). The one downregulated gene by Bex in both sham and CLP is indicated in green; the three upregulated genes by Bex in the CLP condition only are indicated in red. (**F**) Normalized RNA-seq read counts for selected RXR target genes (*Cyp26a1,* *Acot1, Akr1b7,* and* Hes1*) illustrating distinct response patterns to Bex treatment during sepsis (*n* = 3 individual mice per group; analyzed by two-way ANOVA followed by Tukey’s multiple comparisons test). Where applicable, data represent mean ± SD. Exact *p* values are indicated directly within the figure panels. Note: Transcriptomic profiles and normalized read counts (panels **C**, **F**) represent distinct biological replicates (*n*) from a single dedicated treatment cohort. Source data are available online for this figure.

To confirm that sepsis-induced downregulation of RXRα mRNA and predicted pathways indeed compromise RXRα-dependent signaling pathways, we treated WT mice with Bex (a validated pan-RXR agonist) or vehicle (DMSO) 6 h after sham or CLP surgery, and harvested livers 2 h later for bulk RNA-sequencing (Fig. 2A). At this 6-h post-CLP time point, the acute systemic response is actively developing, while late-stage organ failure and secondary complications remain limited. Mice in both the DMSO- and Bex-treated CLP groups developed significant hypothermia compared to their respective sham controls (Fig. 2B).

Principal component analysis (PCA) of hepatic transcriptomes revealed that PC1 (74% variance) was driven by the sepsis effect, clearly separating sham from CLP samples, while PC2 (11% variance) captured the BEX effect (Fig. 2C). Notably, Bex treatment induced a strong transcriptional shift along PC2 in sham-operated mice, whereas this shift was substantially attenuated in septic mice. This suggests that sepsis impairs the hepatic ability to induce a canonical RXR-driven transcriptional response to Bex stimulation.

**Figure EV2:**
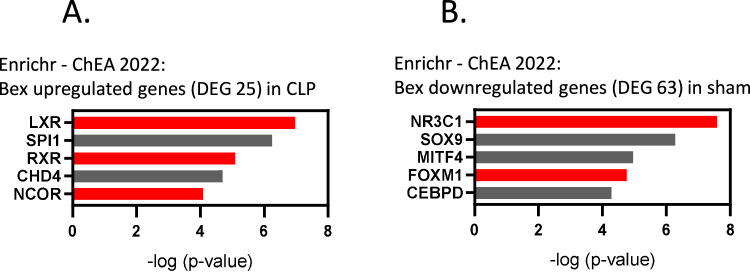
Enrichment analysis of bexarotene treatment. (**A**) Transcription factor (ChEA 2022) enrichment analysis of genes significantly upregulated by Bex in CLP mice compared to DMSO controls (adj. *p* < 0.05) (*n* = 3 individual mice per group). (**B**) Transcription factor (ChEA 2022) enrichment analysis of genes significantly downregulated by Bex in Sham mice compared to DMSO controls (adj. *p* < 0.05). Bars represent negative log10 *p* values. Pathways and regulators directly associated with the RXRα signaling axis are highlighted in red.

Consistent with the PCA plot, differential expression analysis (adj. *p* < 0.05) revealed that 220 genes were upregulated and 63 downregulated by Bex in sham animals, compared to only 25 upregulated and 1 (*Zfp750*) downregulated gene(s) in CLP mice (Fig. 2D). Only three genes (*Hes1, Il10,* and *Tnfsf14*) were uniquely upregulated by Bex in CLP mice. Genes induced uniquely by Bex in the sham condition were significantly enriched for RXR and its known heterodimerization partners (LXR and PPARα), as well as pathways involved in lipid metabolism, fatty acid oxidation, and phospholipid biosynthesis (Fig. 2D). These enrichments confirm that Bex engages canonical RXR signaling in the control liver. When analyzing Bex-upregulated genes in CLP livers, enrichment for LXR and RXR targets was still detected, though with markedly reduced significance, likely due to the lower number of differentially expressed genes (Fig. EV2A). Conversely, genes downregulated by Bex in sham mice were enriched for regulators of inflammation (NR3C1/GR) and cell proliferation (FOXM1) (Fig. EV2B), suggesting that RXR activation in the control liver simultaneously promotes metabolic functions while repressing basal inflammatory and proliferative signals.

To evaluate how the transcriptional response to Bex is altered by sepsis, we performed correlation analysis of log2 fold-changes in response to Bex in sham vs. CLP conditions. The resulting regression line (y = 0.4686x ± 0.05160; *R*^2^ = 0.1986) deviated substantially from the unity line (y = 1), which would indicate equal responses, but was not horizontal either (y = 0) (Fig. 2E). This pattern indicates a broadly dampened RXR response of 46.86% in CLP, where many Bex-responsive genes responded to a lesser extent rather than being completely unresponsive.

We further validated and highlighted selected transcripts representing distinct response patterns (Fig. 2F). *Cyp26a1*, a cytochrome P450 enzyme responsible for retinoic acid catabolism and a canonical RAR/RXR target, and *Acot1*, an acyl-CoA thioesterase linked to PPAR/RXR-driven fatty acid oxidation, were strongly induced by Bex in sham but not in CLP mice, indicating complete resistance. *Akr1b7*, an aldo-keto reductase involved in detoxification and lipid homeostasis and known to be regulated by PPAR/RXR signaling, showed attenuated, although significant residual induction after Bex during CLP compared to sham. In contrast, *Hes1*, a transcriptional repressor downstream of Notch signaling that may be selectively engaged in the inflammatory context, was selectively upregulated by Bex only in CLP mice. Taken together, these results demonstrate that sepsis generally impairs RXRα-dependent gene activation, although some transcriptional responses to RXR agonism remain intact or even become selectively induced in the sepsis-affected liver.

### Bexarotene protects against sepsis-induced mortality and metabolic dysfunction through hepatocyte RXRα

**Figure 3:**
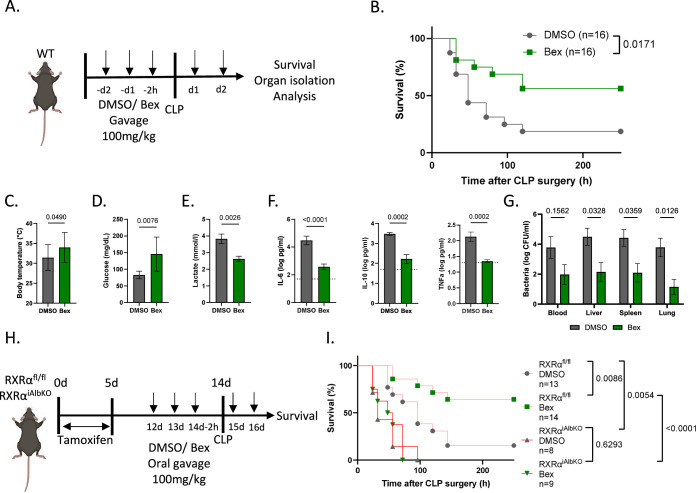
Bexarotene protects against sepsis-induced mortality and metabolic dysfunction through hepatocyte RXRα. (**A**) Schematic of the prophylactic dosing regimen. WT mice received Bex (100 mg/kg) or DMSO via oral gavage daily starting two days prior to CLP, with additional doses 2 h before and 24/48 h after surgery. (**B**) Kaplan–Meier survival curves of WT mice following CLP surgery treated with DMSO or Bex (*n* = 16 mice/group). (**C**–**F**) Physiological and biochemical parameters 24 h post-CLP in WT mice representing individual biological replicates (individual mice): (**C**) body temperature (*n* = 16 mice/group), (**D**) blood glucose (DMSO *n* = 7; Bex *n* = 8 mice), (**E**) blood lactate (DMSO *n* = 7; Bex *n* = 8 mice), and (**F**) plasma IL-6, IL-10, and TNFα levels (*n* = 10 mice/group). Data in panels (**C**–**F**) were analyzed using an unpaired Student’s *t*-test (following log-transformation for biochemical and cytokine parameters in panels **D**–F). (**G**) Bacterial burden (CFU/ml) in blood, liver, spleen, and lungs 24 h post-CLP (DMSO *n* = 13; Bex *n* = 16 individual mice; data were log-transformed and analyzed using a Two-way ANOVA followed by Šídák’s multiple comparisons test). (**H**) Schematic of the experimental design for inducible hepatocyte-specific RXRα-deficient (RXRα^iAlbKO^) and control (RXRα^fl/fl^) mice. (**I**) Survival of RXRα^fl/fl^ and RXRα^iAlbKO^ mice following CLP surgery treated with DMSO or Bex (RXRα^fl/fl^ DMSO *n* = 13 mice; RXRα^iAlbKO^ DMSO *n* = 8 mice; RXRα^fl/fl^ Bex *n* = 14 mice; RXRα^iAlbKO^ Bex *n* = 9 mice). Where applicable, data represent mean ± SD. Survival curves analyzed by the log-rank (Mantel–Cox) test. Exact *p* values are indicated directly within the figure panels. Note: All physiological, biochemical, and survival parameters represent distinct biological replicates (individual mice) and are representative of two independent in vivo experiments performed in the laboratory. Source data are available online for this figure.

To assess the functional impact of RXRα (in)activation during sepsis, WT mice were treated with Bex at multiple time points relative to the CLP surgery. Given that sepsis blunts the transcriptional response to RXR activation (Fig. 2), we utilized a prophylactic-leaning dosing regimen to stimulate RXRα prior to the onset of sepsis-induced signaling impairment (Fig. 3A). Specifically, Bex was administered daily starting two days prior to CLP to ensure robust RXRα activation, as well as after CLP at the indicated time points (Fig. 3A). This dosing regimen significantly improved survival compared to DMSO-treated controls (Fig. 3B), confirming that prophylactic RXR activation improves survival in septic mice.

**Figure EV3:**
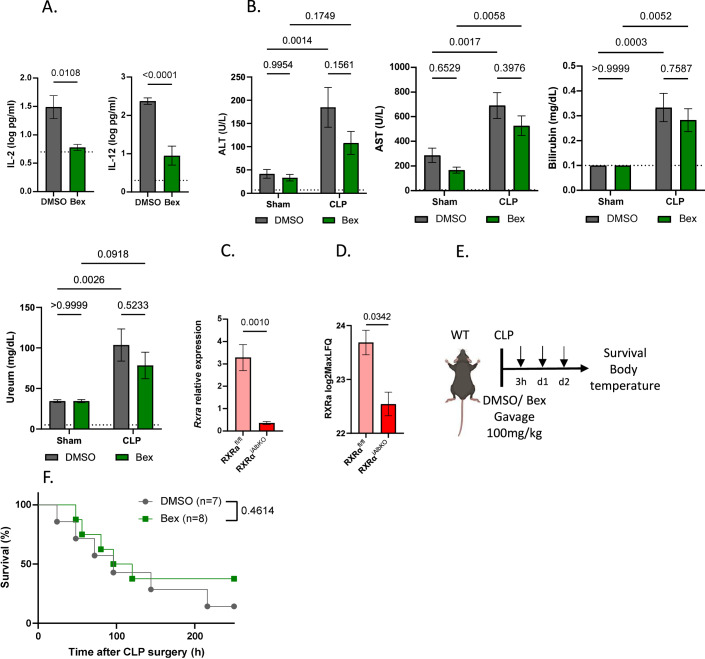
Validation of hepatocyte-specific RXRα deletion and survival analysis of therapeutic bexarotene administration. (**A**) Plasma IL-2 and IL-12 cytokine levels in DMSO and Bex-treated mice after CLP (*n* = 10 individual mice per group; data were log-transformed and analyzed using an unpaired Student’s *t*-test). (**B**) Plasma organ damage parameters (alanine aminotransferase (ALT), aspartate aminotransferase (AST), bilirubin and ureum) in DMSO and Bex-treated mice (*n* = 12 individual mice per group; analyzed using an unpaired Student’s *t*-test). Dashed lines indicate the limit of detection (LOD) for each respective biochemical assay. (**C**) Hepatic *Rxrα* mRNA expression in RXRα^fl/fl^ and RXRα^iAlbKO^ mice was measured using primers specific for the deleted exon 4 (RXRα^fl/fl^ *n* = 9; RXRα^iAlbKO^ n = 6 individual mice; data were log-transformed and analyzed using an unpaired Student’s *t*-test). (**D**) Hepatic RXRα protein abundance (MaxLFQ intensity) in RXRα^fl/fl^ and RXRα^iAlbKO^ livers determined by shotgun proteomics (RXRα^fl/fl^ *n* = 7; RXRα^iAlbKO^ *n* = 8 individual mice; analyzed using a *limma* moderated *t*-test). (**E**) Schematic representation of the therapeutic dosing regimen. Mice were subjected to CLP surgery and subsequently treated with DMSO or Bex (100 mg/kg, oral gavage) at 3, 24, and 48 h post-surgery. (**F**) Survival of septic mice following therapeutic administration of DMSO (*n* = 7) or Bex (*n* = 8 mice per group; analyzed using the log-rank (Mantel–Cox) test). Where applicable, data represent mean ± SD. Exact *p* values are indicated directly within the figure panels.

In addition to improved survival, Bex-treated animals maintained significantly higher body temperature (Fig. 3C) and plasma glucose levels 24 h post-surgery (Fig. 3D), suggesting protection against the hypothermia and hypoglycemia typically observed in severe sepsis. Plasma lactate concentrations were markedly reduced (Fig. 3E). Consistent with this improved metabolic stability, systemic inflammation was attenuated, as reflected by lower circulating IL-2, IL-6, IL-10, IL-12, and TNFα levels, 24 h post-surgery (Figs. 3F and EV3A).

To assess whether Bex modulated bacterial burden, we quantified bacterial colony-forming units (CFUs) in peripheral organs and circulation 24 h after CLP. This time point was selected based on our survival experiment, which showed a significant divergence in body temperature between groups at 24 h that was not yet apparent at 8 h. Bex treatment significantly reduced bacterial loads in the liver, spleen, and lungs, with a strong, though not statistically significant, trend toward reduction in blood (Fig. 3G), suggesting enhanced bacterial clearance or reduced bacterial translocation.

To further evaluate the impact of Bex treatment on sepsis-induced organ dysfunction, we quantified a comprehensive panel of plasma damage markers. Polymicrobial sepsis induced a significant elevation in liver-specific injury markers (AST, ALT, and bilirubin) as well as the renal marker Ureum in DMSO-treated controls (Fig. EV3B). In contrast, the CLP-induced increase for ALT and Ureum was not statistically significant in the Bex-treated group compared to sham controls. While the direct reduction between vehicle- and Bex-treated septic mice did not reach statistical significance, a consistent downward trend was observed for these parameters, indicating that early RXRα activation might limit the trajectory of systemic organ damage and preserves hepatic integrity during the acute phase of infection.

To determine whether the Bex-induced protective effects were hepatocyte-intrinsic and RXRα-dependent, we generated hepatocyte-specific inducible RXRα knockout mice (RXRα^iAlbKO^) and controls (RXRα^fl/fl^) and treated them with the same Bex regimen in an independent survival study (Fig. 3H). While Bex improved survival in control mice, this effect was entirely abolished in RXRα^iAlbKO^ mice (Fig. 3I). This genetic loss-of-function approach confirms that hepatocyte RXRα is required for the protective effects of Bex in sepsis. Furthermore, there was also a difference between RXRα^fl/fl^ and RXRα^iAlbKO^ mice in this sepsis model, showing increased sensitization in the KO group (Fig. 3I).

The KO was validated with primers specific for the floxed sequence in the exon 4 of the RXRα-coding gene *Rxra*, which showed significant loss of mRNA expression (Fig. EV3C). Similar results were obtained for protein levels using shotgun proteomics (Fig. EV3D).

To assess the therapeutic potential of RXRα activation after the onset of sepsis, we administered Bex post-CLP (at 3, 24, and 48 h) (Fig. EV3E). However, this post-treatment failed to improve survival compared to DMSO-treated controls (Fig. EV3F). The lack of benefit is consistent with the transcriptional resistance observed during early sepsis (Fig. 2), suggesting that therapeutic intervention at the level of RXRα is ineffective once hepatic RXRα signaling has been blunted.

### Hepatic RXRα deficiency exacerbates sepsis-induced transcriptional and proteomic reprogramming and impairs the liver antimicrobial program

**Figure 4:**
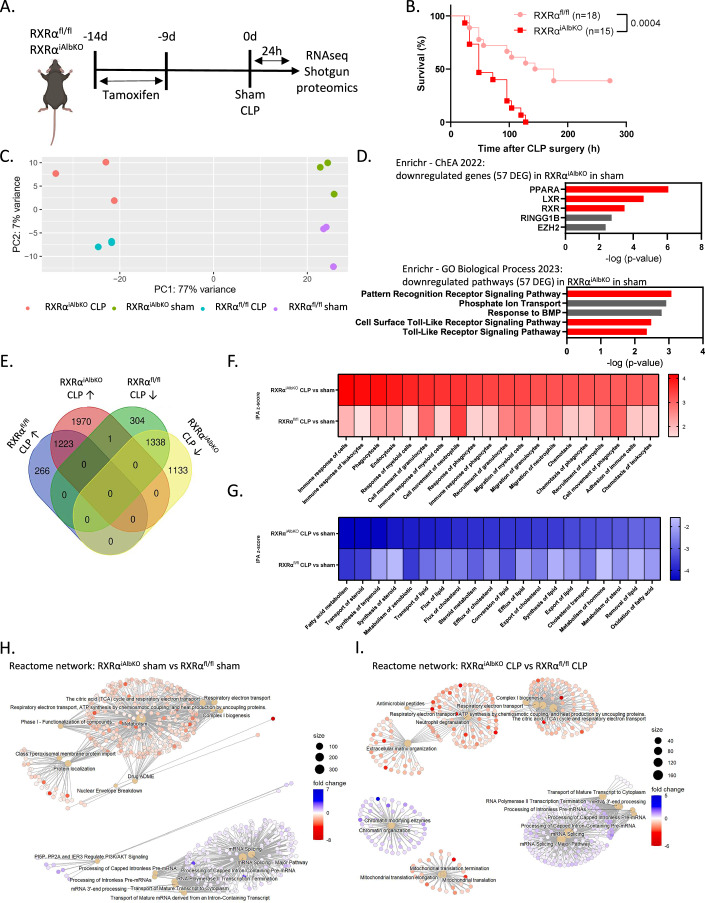
Hepatocyte RXRα deficiency exacerbates sepsis-induced transcriptional and proteomic reprogramming and impairs the hepatic antimicrobial program. (**A**) Experimental design for inducible hepatocyte-specific RXRα deletion (RXRα^iAlbKO^) and control (RXRα^fl/fl^) mice followed by sublethal CLP or sham surgery. (**B**) Kaplan–Meier survival curves of RXR^fl/fl^ (*n* = 18) and RXRα^iAlbKO^ (*n* = 15) mice following moderate CLP surgery. (**C**) Principal component analysis (PCA) of hepatic transcriptomes from RXRα^fl/fl^ and RXRα^iAlbKO^ mice 24 h after sham or CLP surgery (*n* = 3 mice/group). (**D**) Transcription factor (ChEA 2022) and pathway (GO Biological Process 2023) enrichment analysis of genes significantly downregulated in Sham RXRα^iAlbKO^ vs. RXRα^fl/fl^ livers. Pathways and regulators directly associated with the RXRα signaling axis are highlighted in red. (**E**) Venn diagram illustrating the number and overlap of differentially expressed genes (DEGs) (adj. *p* < 0.05) in RXRα^fl/fl^ and RXRα^iAlbKO^ mice 24 h after CLP compared to their respective sham controls. (**F**,** G**) Ingenuity pathway analysis (IPA) comparison of (**F**) inflammatory/immune pathways and (**G**) metabolic/antimicrobial pathways in RXRα^fl/fl^ and RXRα^iAlbKO^ livers after CLP. Heatmap colors represent activation Z-scores. (**H**) Reactome network analysis of the nuclear-enriched proteome in Sham RXRα^iAlbKO^ (*n* = 4) vs. RXRα^fl/fl^ (*n* = 3) livers. (**I**) Reactome network analysis of the nuclear-enriched proteome in CLP RXRα^iAlbKO^ vs. RXRα^fl/fl^ livers (*n* = 4/group). Where applicable, data represent mean ± SD. Survival curves analyzed by the log-rank (Mantel–Cox) test. Exact *p* values are indicated directly within the figure panels. Note: Transcriptomic and proteomic profiles (panels **C**, **H**, **I**) represent distinct biological replicates (*n*) from a single dedicated discovery cohort. Survival data are representative of two independent in vivo experiments. Source data are available online for this figure.

Building on the observation that hepatocyte RXRα loss potentially sensitizes mice to septic death (Fig. 3I), we sought to further dissect the molecular basis of RXRα-dependent hepatoprotection in sepsis. To do so, we performed a low-lethality CLP (achieved by ligating a smaller portion of the cecum to reduce the severity of the infection) in hepatocyte-specific inducible RXRα knockout (RXRα^iAlbKO^) and littermate control (RXRα^fl/fl^) mice (Fig. 4A). By utilizing a model in which mortality in WT mice is moderate, we reasoned it might enable a clearer disentanglement of sensitization in the KO mice. While mortality was ~60% in the control group, RXRα^iAlbKO^ mice showed 100% mortality following CLP (Fig. 4B), confirming that hepatocyte RXRα is essential for host defense against sepsis.

**Figure EV4:**
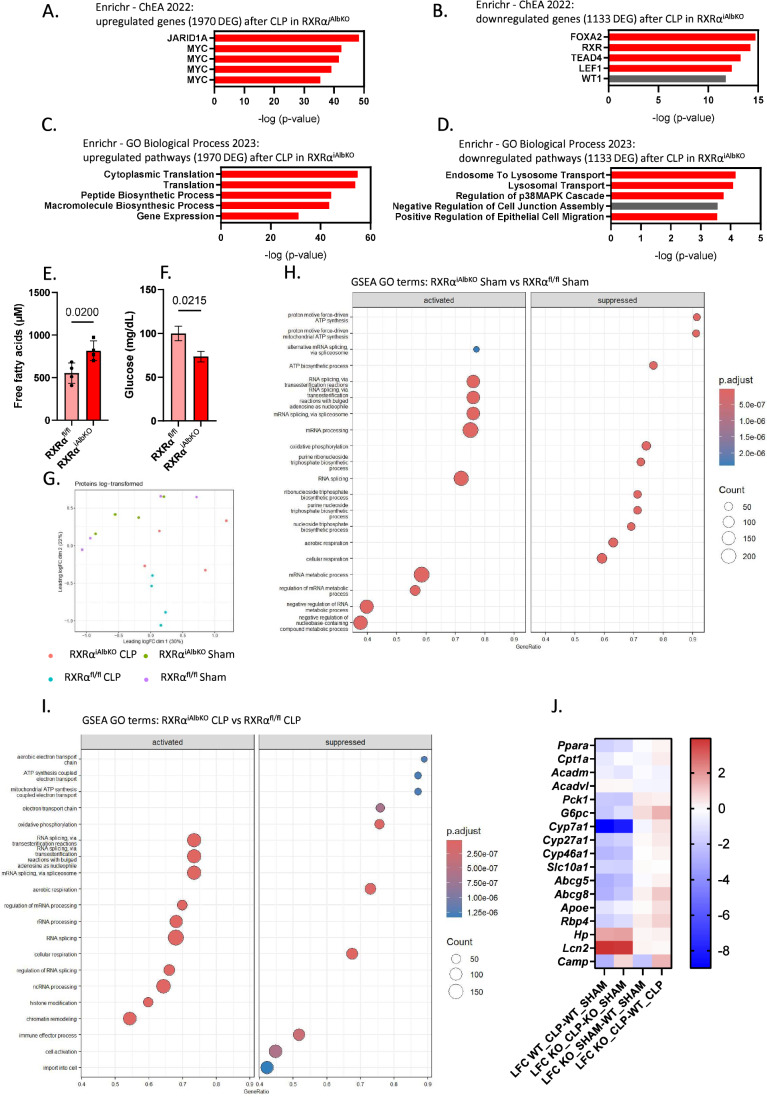
Transcriptomic and proteomic landscape of the septic liver in the absence of hepatocyte RXRα. (**A**–**D**) Transcription factor (ChEA 2022) and pathway (GO Biological Process 2023) enrichment analysis of (**A**, **C**) upregulated and (**B**, **D**) downregulated DEGs (adj. *p* < 0.05) in RXRα^iAlbKO^ vs. RXRα^fl/fl^ livers following CLP (*n* = 3 individual mice per group). Pathways and regulators directly associated with the RXRα signaling axis are highlighted in red. (**E**) Plasma free fatty acids (FFAs) concentration 24 h post-CLP (*n* = 4 individual mice per group; analyzed using an unpaired Student’s *t*-test). (**F**) Blood glucose levels 24 h post-CLP (RXRα^fl/fl^ *n* = 8; RXRα^iAlbKO^ *n* = 11 individual mice; analyzed using an unpaired Student’s *t*-test). (**G**) PCA plot of the nuclear-enriched proteome from RXRα^fl/fl^ and RXRα^iAlbKO^ livers 24 h after sham or CLP surgery. (**H**) Gene set enrichment analysis (GSEA) of the proteome in Sham RXRα^iAlbKO^ vs. RXRα^fl/fl^ livers. (**I**) GSEA of the proteome in CLP RXRα^iAlbKO^ vs. RXRα^fl/fl^ livers. Dot size in GSEA represents gene count, and color indicates the adjusted p-value. (**J**) Heatmap of LFC for representative candidate genes involved in master regulation of fatty acid oxidation, gluconeogenesis, oxysterol and bile acid synthesis, lipid and retinoid transport, and antimicrobial defense across the specified experimental comparisons. Where applicable, data represent mean ± SD. Exact *p* values are indicated directly within the figure panels.

Subsequently, RNA-seq analysis of whole livers harvested 24 h after CLP revealed distinct transcriptional profiles between genotypes and treatments, as shown by PCA (Fig. 4C). At baseline (sham), RXRα^iAlbKO^ livers displayed broad transcriptional changes, with enrichment analysis highlighting downregulation of canonical RXR, PPARα, and LXR signaling pathways, together with impaired immune-associated programs (Fig. 4D). A granular analysis of candidate genes revealed that even in the absence of infection, the loss of RXRα resulted in a basal suppression of the antimicrobial effector *Camp* (LFC −2.10) and the rate-limiting fatty acid transporter *Cpt1a* (LFC −0.35) (Fig. EV4J). This suggests that RXRα deficiency creates an “immunometabolic fragility” that precedes the septic insult.

In the CLP condition, analysis of DEGs revealed an extensive overlap in the response, but RXRα^iAlbKO^ mice exhibited 1133 unique downregulated and 1970 unique upregulated genes, a substantially higher number of genotype-specific DEGs compared to controls (Fig. 4E). This suggests that while the core septic response remains intact, the loss of RXRα significantly exacerbates and rewires the hepatic transcriptional program.

To quantify these differences, we compared the activation Z-scores of biological pathways between genotypes. Enrichr-based analysis of CLP-induced changes in the KO showed preferential upregulation of regenerative (MYC) and epigenetic regulation (JARID1A) pathways (Fig. EV4A,C), while canonical RXR and FOXA2 pathways were among the most downregulated (Fig. EV4B,D). Crucially, IPA comparison revealed that while RXRα^iAlbKO^ mice showed exaggerated induction of inflammatory pathways such as “Immune response of cells” and “Phagocytosis” (Fig. 4F), they simultaneously displayed a significantly more pronounced repression of metabolic programs, including “Fatty acid metabolism” and “Oxidation of fatty acid”, compared to RXR^fl/fl^ controls (Fig. 4G). This was reflected at the gene level by a failure to maintain the expression of mitochondrial β-oxidation genes (*Ppara* and *Acadm*) and the primary gluconeogenic driver *Pck1* (LFC −1.95) (Fig. EV4J). Furthermore, the septic RXRα^iAlbKO^ livers exhibited a profound suppression of the enzymatic machinery and transporters required for oxysterol synthesis and lipid/retinoid efflux (e.g., Cyp27a1, Cyp46a1, Abcg5, Abcg8, and Rbp4), pointing toward a severe transcriptional failure in the production and export of essential lipid mediators (Fig. EV4J). These parallel-omics findings were functionally supported by biochemical analysis 24 h post-CLP, which revealed significantly higher plasma free fatty acid (FFA) levels and lower blood glucose in RXRα^iAlbKO^ mice compared to controls (Fig. EV4E,F). This suggests that the observed transcriptomic and proteomic repression of fatty acid oxidation directly translates into a systemic metabolic collapse, characterized by the accumulation of unused lipids and the failure to maintain glucose homeostasis.

Proteomic profiling of nuclear-enriched liver fractions corroborates our RNA-seq findings. Global proteome analysis showed robust clustering of CLP versus sham samples by PCA, with a distinct separation between genotypes in the septic condition (Fig. EV4G). Pathway analysis of the proteomics dataset partially corroborated the transcriptomic findings. In the basal state (KO vs WT sham), RXRα^iAlbKO^ livers exhibited dysregulation of metabolic processes and upregulation of RNA processing (Figs. 4H and EV4H). Interestingly, while the transcriptome showed a compensatory rise in some immune markers, the proteomic analysis of septic RXRα^iAlbKO^ mice revealed a pronounced repression of antimicrobial and metabolic programs alongside exaggerated activation of RNA splicing and stress responses (Figs. 4I and EV4I).

Together, these parallel-omics data establish that hepatocyte RXRα is critical for maintaining homeostasis in the antimicrobial and metabolic landscape during sepsis. Loss of RXRα sensitizes the liver and whole animal to exaggerated inflammatory activation while critically impairing metabolic and antimicrobial effector functions, thereby worsening outcomes.

### Kupffer cell loss drives impaired bacterial clearance and increased sepsis susceptibility in RXRα^iAlbKO^ mice

**Figure 5:**
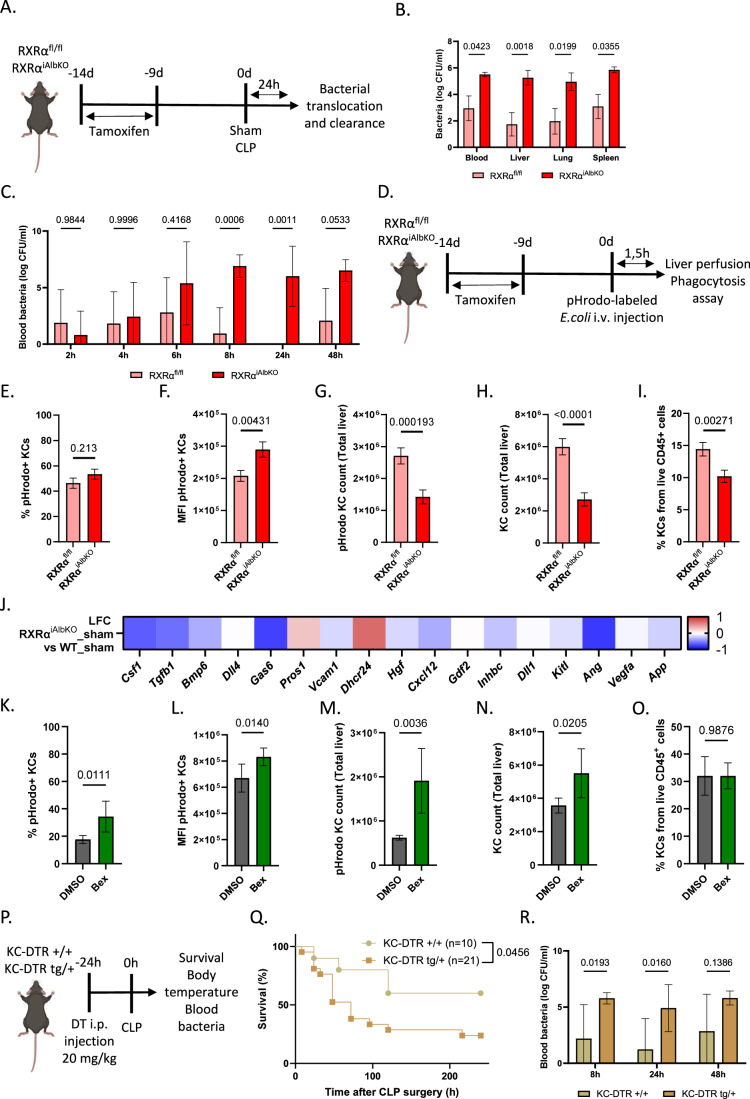
Kupffer cell loss drives impaired bacterial clearance and increased sepsis susceptibility in RXRα^iAlbKO^ mice. (**A**) Experimental design for inducible hepatocyte-specific RXRα deletion (RXRα^iAlbKO^) followed by CLP and organ isolation 24 h post-surgery. (**B**) Bacterial loads (log CFU/ml or CFU/mg) in blood, liver, lung, and spleen 24 h post-CLP in RXRα^fl/fl^ (*n* = 9) and RXRα^iAlbKO^ (*n* = 11) mice (analyzed by two-way ANOVA). (**C**) Time-course analysis of blood bacterial burden at 2, 4, 6, 8, 24, and 48 h post-CLP in RXRα^fl/fl^ (*n* = 6) and RXRα^iAlbKO^ (*n* = 7) mice (analyzed by two-way ANOVA). (**D**) Schematic representation of the in vivo phagocytosis assay using intravenous injection of pHrodo-labeled *E. coli* 1.5 h prior to liver perfusion. (**E**) Percentage of pHrodo-positive Kupffer cells (KCs) among total KCs determined by flow cytometry (*n* = 8/group, analyzed via generalized linear model). (**F**) Mean fluorescence intensity (MFI) of pHrodo-positive KCs in RXRα^fl/fl^ and RXRα^iAlbKO^ livers (*n* = 8/group, analyzed via generalized linear model). (**G**) Absolute number of pHrodo-positive (phagocytosing) KCs per liver (*n* = 8/group, analyzed via generalized linear model). (**H**) Total Kupffer cell counts per liver in RXRα^fl/fl^ and RXRα^iAlbKO^ mice (*n* = 8/group, analyzed via generalized linear model). (**I**) Percentage of KCs among total CD45-positive hepatic leukocytes (*n* = 8/group, analyzed via generalized linear model). (**J**) Heatmap of log_2_ fold-changes (LFC) (bulk RNA-seq) for hepatocyte-derived niche factors and signaling molecules essential for Kupffer cell homeostasis in naïve RXRα^fl/fl^ and RXRα^iAlbKO^ livers (*n* = 3/group; one-sample *t*-test). (**K**) Percentage of pHrodo-positive KCs among total KCs in DMSO- (*n* = 5) and Bex-treated (*n* = 6) mice. (**L**) Mean fluorescence intensity (MFI) of pHrodo-positive KCs in DMSO- (*n* = 5) and Bex-treated (*n* = 6) mice. (**M**) Absolute number of pHrodo-positive (phagocytosing) KCs per liver in DMSO- (*n* = 5) and Bex-treated (*n* = 6) mice. (**N**) Total Kupffer cell counts per liver in WT mice subjected to CLP and treated prophylactically with vehicle (DMSO) (*n* = 5) or Bex (*n* = 6). (**O**) Percentage of KCs among total CD45-positive hepatic leukocytes in DMSO- (*n* = 5) and Bex-treated (*n* = 6) mice post-CLP. Panels (**K**–O) were analyzed using an unpaired Student’s *t*-test. (**P**) Experimental scheme for selective Kupffer cell depletion using DT injection (20 μg/kg) in KC-DTR mice 24 h prior to CLP. (**Q**) Kaplan–Meier survival curves of control (KC-DTR +/+; *n* = 10) and KC-depleted (KC-DTR tg/+; *n* = 21) mice following CLP (log-rank Mantel–Cox test). (**R**) Time-course analysis of blood bacterial burden at 8, 24, and 48 h post-CLP in control and KC-depleted mice (*n* = 5 +/+, *n* = 8 tg/+; analyzed by two-way ANOVA). Where applicable, data represent mean ± SD. Exact *p* values are indicated directly within the figure panels. Note: Flow cytometry, bacterial burden, and phagocytosis parameters represent distinct biological replicates (individual mice) and are representative of two independent in vivo experiments. Transcriptomic data (panel **J**) represent distinct biological replicates (*n*) from a single dedicated transcriptomic cohort. Source data are available online for this figure.

**Figure EV5:**
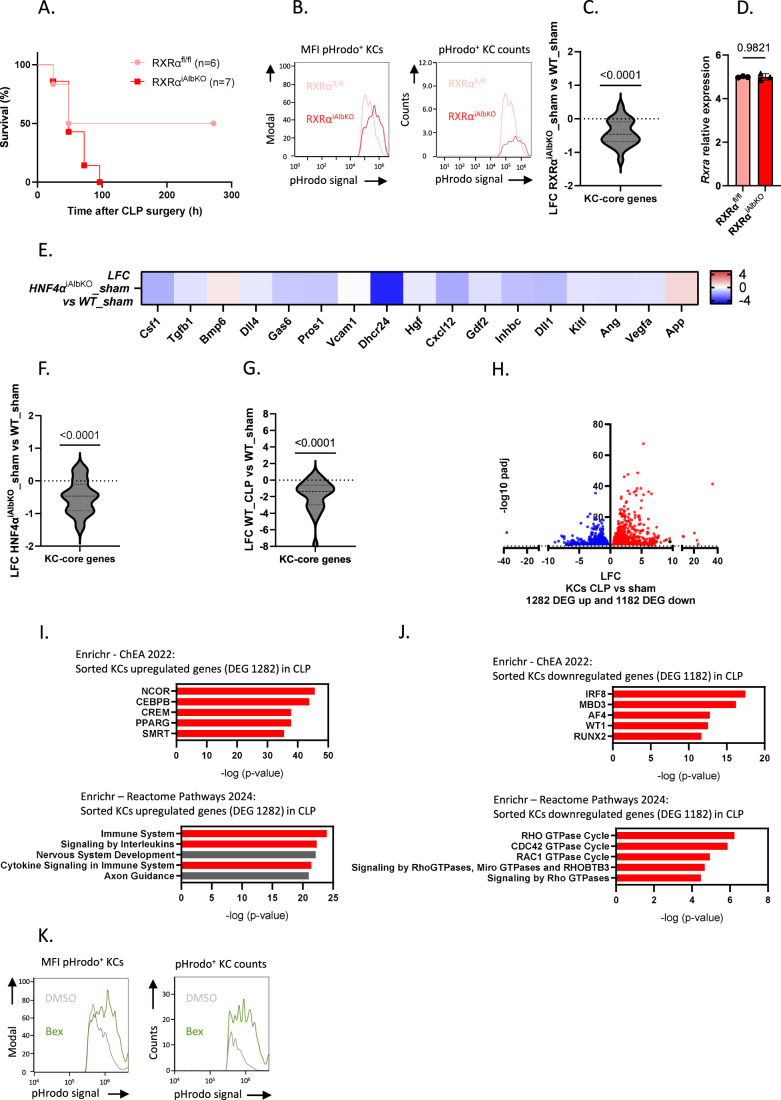
Transcriptomic and functional characterization of Kupffer cells in RXRα deficiency and sepsis. (**A**) Survival curve corresponding to the time-course analysis of bacterial dissemination (Fig. 5C) (RXRα^fl/fl^ *n* = 6 and RXRα^iAlbKO^ *n* = 7 individual mice; analyzed using the log-rank (Mantel–Cox) test). (**B**) Representative spectral flow cytometry plots demonstrating in vivo pHrodo-labeled *E. coli* uptake (MFI) in naïve RXRα^fl/fl^ and RXRα^iAlbKO^ mice. (**C**) Log_2_ fold-change (LFC) of the KC-core gene signature in bulk liver RNA-seq from sham RXRα^iAlbKO^ mice compared to littermate controls (*n* = 3 independent sequencing replicates per group; analyzed using a one-sample *t*-test against a theoretical LFC mean of 0). (**D**) Relative mRNA expression of *Rxra* in FACS-sorted KCs using deleted exon-specific primers (*n* = 3 independent biological replicates per group; data were log-transformed and analyzed using an unpaired Student’s *t*-test). (**E**) LFC of essential KC niche factors in bulk liver RNA-seq from sham HNF4α^iAlbKO^ mice compared to littermate controls (*n* = 4 independent sequencing replicates per group). (**F**) LFC of the KC-core gene signature in bulk liver RNA-seq from sham HNF4α^iAlbKO^ mice compared to littermate controls (*n* = 4 independent sequencing replicates per group; analyzed using a one-sample *t*-test against a theoretical LFC mean of 0). (**G**) LFC of the KC-core gene signature in bulk liver RNA-seq from WT mice 8 h post-CLP compared to sham (*n* = 3 independent sequencing replicates per group; analyzed using a one-sample *t*-test against a theoretical LFC mean of 0). (**H**) Volcano plot displaying differentially expressed genes (DEGs; adj. *p* < 0.05) in FACS-sorted KCs from WT mice following CLP compared to sham surgery. (**I**) Pathway enrichment analysis of significantly upregulated genes (adj. *p* < 0.05) in FACS-sorted KCs following CLP. (**J**) Pathway enrichment analysis of significantly downregulated genes (adj. *p* < 0.05) in FACS-sorted KCs following CLP. (**K**) Representative spectral flow cytometry plots demonstrating pHrodo-labeled *E. coli* uptake (MFI) in WT mice subjected to CLP and treated prophylactically with DMSO or Bexarotene. Where applicable, data represent mean ± SD. Exact *p* values are indicated directly within the figure panels.

Investigating the functional consequences of the impaired antimicrobial programs identified in our parallel-omics analysis in Fig. 4, we subjected RXRα^iAlbKO^ and control mice to CLP and assessed bacterial burden 24 h later (Fig. 5A). Given that RXRα activation by BEX reduced bacterial loads, and our proteomic data indicated a failure of antimicrobial effector production in the RXRα^iAlbKO^, we hypothesized that RXRα deficiency would lead to a failure in systemic pathogen control. Reciprocally to the effects of RXRα activation, RXRα deletion caused markedly higher bacterial loads after CLP in multiple organs (blood, liver, spleen, and lungs) compared to RXRα^fl/fl^ control CLP mice (Fig. 5B), indicating impaired systemic bacterial clearance. Time-course analysis further revealed that, while RXRα^fl/fl^ animals restrained bacterial expansion within 24 h post-CLP, RXRα^iAlbKO^ mice failed to control bacterial growth, leading to progressive bacterial accumulation (Fig. 5C) and increased susceptibility to CLP-induced lethality (Fig. EV5A).

To determine whether this phenotype might involve altered phagocytic function of liver-resident macrophages, we assessed Kupffer cell (KC) phagocytosis in naïve mice using i.v. injection of pHrodo-labeled *E. coli* in vivo (Fig. 5D). The proportion of pHrodo⁺ Kupffer cells was comparable between RXRα^fl/fl^ and RXRα^iAlbKO^ mice (Fig. 5E), suggesting that the probability of bacterial uptake by individual KCs is not altered by hepatocyte RXRα loss. However, KCs in RXRα^iAlbKO^ mice exhibited significantly higher mean fluorescence intensity (MFI) (Figs. 5F and EV5B), implying enhanced per-cell phagosomal bacterial uptake in the remaining cells, which likely reflects a functional compensatory mechanism by the surviving macrophage pool. However, the absolute number of phagocytosing KCs per liver was twofold reduced in the RXRα^iAlbKO^ mice (Figs. 5G and EV5B). Consistently, both the total KC population (Fig. 5H) and their fraction within CD45⁺ hepatic leukocytes (Fig. 5I) were significantly diminished in naïve RXRα^iAlbKO^ mice, indicating a shift in liver-immune composition secondary to hepatocyte RXRα loss.

To elucidate the mechanism driving this KC depletion, we analyzed the expression of liver cells-derived niche factors (except for KCs) known to maintain KC survival and identity (Bonnardel et al, 2019; Sakai et al, 2019; Tutusaus et al, 2020). We observed a global downregulation of essential niche-supportive mRNAs, including *Csf1, Tgfb1, and Bmp6* (Fig. 5J). This reduction in the niche-maintaining transcriptome not expressed in KCs suggests that hepatocyte RXRα is required to maintain the molecular environment necessary for KC viability. Consistent with a disrupted hepatic microenvironment and lower KC numbers, bulk liver RNA-seq confirmed a significant downregulation (*p* < 0.0001) of the KC-core identity signature (e.g., *Clec4f, Timd4,* and *Vsig4*) in sham RXRα^iAlbKO^ compared to control liver (Fig. EV5C), which directly reflects the physical reduction of the Kupffer cell pool within the bulk tissue. To exclude that this KC depletion was due to direct genetic deletion of *Rxra* in the myeloid lineage due to the aspecificity of the albumin cre, we performed fluorescence-activated cell sorting (FACS) to isolate KCs from both naïve RXRα^fl/fl^ and RXRα^iAlbKO^ mice. qPCR analysis using primers specific for the deleted region revealed no difference in *Rxra* transcript levels between KCs derived from RXRα^fl/fl^ and RXRα^iAlbKO^ mice (Fig. EV5D). Because our earlier data established HNF4α as the upstream transcriptional driver of hepatic Rxra, we queried our published bulk RNA-seq dataset from hepatocyte-specific HNF4α knockout mice (HNF4α^iAlbKO^) (GSE260635). Consistent with the HNF4α-RXRα regulatory axis, HNF4α-deficient livers exhibited a similar and significant downregulation of both the essential hepatocyte-derived niche factors (Fig. EV5E) and the KC-core identity signature (Fig. EV5F), indicating that disruption of this upstream regulator also compromises the Kupffer cell niche. To contextualize the loss of KCs within the framework of acute infection, we assessed the status of the KC-core signature in wild-type mice following sepsis. Our bulk RNA-seq comparison of CLP versus Sham livers revealed a significant downregulation (*p* < 0.0001) across the KC-core identity genes (Fig. EV5G). Collectively, these data confirm that the Alb-Cre-mediated deletion is hepatocyte-specific and indicate that the observed loss of Kupffer cells is an indirect consequence of a disrupted hepatic niche following RXRα deletion.

We evaluated the transcriptomic reprogramming of Kupffer cells independent of their physical depletion within the bulk liver, we performed RNA-seq on FACS-sorted KCs isolated from WT mice following sham or CLP surgery. Differential expression and pathway enrichment analysis revealed that surviving KCs in septic livers undergo profound phenotypic shifts (2464 DEGs) (Fig. EV5H). The upregulated transcriptome in the surviving KCs was prominently enriched for inflammatory signatures (Fig. EV5I). Conversely, the downregulated transcriptome showed a significant suppression of pathways associated with Rho GTPase signaling (Fig. EV5J). Given that Rho GTPases are fundamental regulators of actin cytoskeleton dynamics and phagocytic cup formation in macrophages, this transcriptomic signature suggests that the KCs surviving the acute septic insult not only adopt a hyper-inflammatory phenotype, but also become fundamentally compromised in their structural and phagocytic competence.

Furthermore, to determine whether pharmacological activation of RXRα can counter this infection-induced macrophage depletion and improve functional bacterial clearance, we evaluated KC populations and their phagocytic capacity in WT mice subjected to CLP and treated prophylactically with either vehicle (DMSO) or Bex. Consistent with our survival and bacterial burden data (Fig. 3), when assessing in vivo phagocytosis using pHrodo-labeled *E. coli*, we found that Bex treatment significantly increased both the percentage of pHrodo^+^ KCs actively engulfing bacteria (Fig. 5K) and their per-cell phagocytic capacity (measured as the mean fluorescence intensity (MFI), which reflects a higher quantity of internalized bacteria within acidic phagosomes per cell) (Fig. 5L). Consequently, the absolute number of phagocytosing KCs per liver was approximately twofold higher in the Bex-treated cohort (Fig. 5M). Bex treatment significantly preserved the total number of KCs per liver (Fig. 5N), although their overall proportion within the hepatic CD45⁺ leukocyte pool remained comparable to DMSO-treated septic controls (Fig. 5O). This demonstrates that RXR agonism enhances systemic bacterial clearance through a dual mechanism. It not only preserves the total pool of resident KCs available to filter pathogens, but it also boosts their intrinsic phagocytic activity during the acute phase of sepsis.

Finally, to assess whether the increased sepsis susceptibility could be explained by a reduction in Kupffer cell count, we used KC-DTR mice (Scott et al, 2016), in which selective KC ablation was induced prior to CLP (Fig. 5P). KC-depleted mice displayed increased mortality (Fig. 5Q) and elevated blood bacterial burdens (Fig. 5R), phenocopying the response observed in RXRα^iAlbKO^ animals. These findings demonstrate that hepatocyte RXRα is critical for maintaining the Kupffer cell niche, and that its loss causes reduced KC numbers and compromise hepatic antibacterial defense, resulting in increased susceptibility to sepsis.

## Discussion

Our study establishes hepatocyte RXRα as a central integrator of metabolic and innate immune programs that determine host resilience in polymicrobial sepsis, operating downstream of HNF4α and upstream of a broader RXR heterodimer network (PPARα, LXR). Our parallel-omics and genetic loss-of-function data collectively support four interlinked conclusions that emerge directly from the results: (1) HNF4α is required to maintain basal hepatic *Rxra* expression; (2) RXRα expression and protein abundance are rapidly suppressed during early CLP-induced sepsis; (3) sepsis induces a state of partial “RXR resistance” in which the transcriptional response to pharmacological RXR activation (Bex) is severely blunted, and (4) hepatocyte RXRα is necessary for the survival benefit afforded by RXR activation and for maintenance of Kupffer-cell (KC) populations that mediate hepatic bacterial clearance. Together, these findings define an HNF4α-RXRα-coupled functional cascade whose disruption correlates with both metabolic collapse and impaired local immune defense during systemic sepsis.

Two factors drive the loss of RXR signaling in sepsis: the reduction of its transcriptional driver, HNF4α, and the direct impact of inflammatory signaling. First, genetic inactivation of HNF4α markedly lowers *Rxra* transcript levels under basal and septic conditions, demonstrating a direct transcriptional dependency. Cross-species studies in mice, pigs, and humanized liver models consistently identify HNF4α as a central regulator in polymicrobial sepsis, where its broad suppression leads to loss of metabolic gene programs and heightened inflammatory responses (Halimi et al, 2024; Van Dender et al, 2024, 2025a). Our findings in canine sepsis further reinforce this model, as the top downregulated pathways in septic dogs are governed by the RXR heterodimer network, specifically PPARα and LXR. Because sepsis triggers the rapid downregulation of HNF4α (Van Dender et al, 2024), this likely represents the primary upstream driver of *Rxra* mRNA loss. Second, in WT CLP mice, we observe reduced RXRα mRNA and protein by 8 h, indicating that acute inflammation further accelerates RXRα suppression. This inflammatory suppression might be mediated by the binding of pro-inflammatory transcription factors, such as NF-κB and c-Jun, to the Rxra promoter and distal enhancers, which directly represses its transcription (Balasubramaniyan et al, 2016). The combination of HNF4α deficiency and CLP leads to a synergistic collapse: the loss of the transcriptional driver reduces the total receptor pool, but additional mechanisms may be at play, as inflammatory cytokines may also accelerate the post-translational degradation of RXRα protein (Sturm et al, 2005).

Beyond abundance, RXR’s function is compromised at the level of transcriptional competence. A pan-RXR agonist, Bex, robustly reprograms gene expression in sham livers and elicits only a muted response 8 h after CLP. This pattern argues for a mixed state of partial resistance rather than a binary on/off loss: most canonical RXR targets (e.g., *Cyp26a1* and *Acot1*) lose responsiveness entirely, others (e.g., Akr1b7) show attenuated induction, and a very small subset are selectively upregulated only in the septic context. These gene-selective outcomes imply that RXR activity in sepsis is determined not only by receptor abundance, but also by context-dependent factors such as heterodimer partner availability and the altered cellular signaling context that can rewire transcriptional networks. Given these results, the term “RXR resistance” captures two mechanistic layers: (I) a quantitative loss of receptor (lowered RXRα transcript/protein) and (II) a qualitative deficit of transcriptional competence (failure to respond to pharmacological stimulation).

The functional readouts tie RXRα biology directly to sepsis outcomes. Prophylactic and early post-CLP Bex treatment preserved core physiological parameters (including controlled body temperature, blood glucose, and lactate levels), improved cytokine levels, reduced organ bacterial burdens, and improved survival. The maintenance of glycemia and reduction of plasma lactate in Bex-treated animals suggest a robust preservation of hepatic gluconeogenesis and improved metabolic clearance. This might prevent the transition into the terminal hypometabolic state of sepsis, where a failure in hepatic glucose output often coincides with a lethal drop in core body temperature. These protective effects were abolished in RXRα^iAlbKO^ mice, which conversely exhibited severe metabolic failure and higher bacterial loads. This suggests that RXRα acts as an “immunometabolic hub”: by maintaining hepatocellular metabolic competence, it preserves the specialized environment required for local immune functions. Our results align with earlier work showing that Bex reduces inflammatory mediators in endotoxemia and improves lipid homeostasis in non-septic contexts (Lalloyer et al, 2006; Tunctan et al, 2018). Furthermore, genome-wide and transcriptomic analyses demonstrate that pharmacological RXR agonism actively diverts the receptor from its basal homodimeric state to form permissive heterodimers with partners such as PPARα, LXR, and FXR (Menéndez-Gutiérrez et al, 2015; Menéndez-Gutiérrez and Ricote, 2017). In the liver, Bex strongly increases RXR recruitment to shared genomic response elements extensively co-occupied by these heterodimer partners, driving distinct transcriptional programs that regulate metabolism (Boergesen et al, 2012). This ligand-induced shift, which heavily favors heterodimer-driven pathways over RXR homodimers, provides a mechanistic basis for the metabolic stabilization observed here. The loss of RXRα responsiveness once systemic inflammation and HNF4α reduction are established explains why Bex was protective only when administration started before CLP, whereas therapeutic treatment conferred no survival benefit.

Parallel-omics profiling of RXRα^iAlbKO^ livers reveals a pre-existing vulnerability (even before infection) with downregulation of canonical metabolic RXR/PPARα/LXR programs and altered immune-associated signatures. After CLP, RXRα^iAlbKO^ livers show exaggerated induction of stress and regenerative pathways (MYC), and even stronger repression of metabolic programs. Interestingly, while IPA of the transcriptome suggested an increase in some inflammatory markers, functional and proteomic data revealed a net failure of the antimicrobial effector program. In accordance with our results, RXR deletion in hematopoietic stem cells has been shown to induce MYC pathway upregulation (Menéndez-Gutiérrez et al, 2023). This convergence on MYC-driven modules suggests that RXRα and HNF4α act as gatekeepers that restrain the maladaptive rewiring of hepatocyte programs during the transition to sepsis (Van Dender et al, 2025b). Their absence leads to a fragilized hepatic state characterized by elevated biosynthetic demand, impaired metabolic flexibility, and diminished capacity to support immune homeostasis. Of note, the RNA-seq (whole-liver) and shotgun proteomics (nuclear fraction) datasets are not one-to-one, so an overlap might be misleading.

A major functional consequence of this dysregulation is the striking increase in bacterial loads across multiple organs in RXRα^iAlbKO^ mice, accompanied by the failure to contain bacterial expansion over time. These findings identify hepatocyte RXRα as a central upstream regulator of systemic antibacterial defense, acting not only through hepatocyte-intrinsic pathways but also through its essential role in sustaining liver–immune communication. Although per-cell phagocytic MFI of KCs was elevated in RXRα^iAlbKO^ mice, suggesting enhanced compensatory activity among the remaining macrophages, the significant reduction in both the number of phagocytosing KCs and total KC abundance indicates that cellular loss, rather than impaired phagocytic competence, is the primary cause of failed bacterial clearance. This conclusion is supported by our bulk RNA-seq analysis, which revealed a significant downregulation of the KC-core gene signature (e.g., *Clec4f, Timd4*, and* Vsig4*) (Bonnardel et al, 2019) in naïve RXRα^iAlbKO^ livers. As these identity markers are not expressed by hepatocytes, their suppression at the bulk liver level provides independent validation that the resident macrophage pool is physically reduced. Additionally, our data shows that a similar decline in these identity markers occurs in WT mice during sepsis, confirming that the KC population is a vulnerable niche during the progression of the disease. The KC-DTR model phenocopied this susceptibility, providing evidence that KC reduction alone is sufficient to reproduce the antimicrobial defect observed in hepatocyte RXRα deficiency. It is important to note that while KC-DTR induces a near-complete depletion (Scott et al, 2016), the roughly twofold reduction observed in the RXRα^iAlbKO^ model is already sufficient to compromise host defense. This is particularly relevant as recent evidence highlights that CLP itself causes a rapid 27–50% loss of Kupffer cells within 6–24 h, where a greater magnitude of loss directly correlates with higher bacterial load and increased mortality (Li et al, 2025). This was also corroborated in our RNA-seq dataset. Collectively, our results suggest that RXRα deficiency effectively “pre-depletes” the KC niche, leaving the host with a dangerously low baseline of resident macrophages that might reach a state of near-total exhaustion during the secondary insult of sepsis.

Perturbations in lipid metabolism are known to destabilize tissue-resident macrophage niches (Scott and Guilliams, 2018), and KC identity depends on continuous cues from hepatocytes, LSECs, and stellate cells (Bonnardel et al, 2019). Within this framework, hepatocyte RXRα seems to be a key regulator of the metabolic environment that supports KC survival. Crucially, our transcriptomic analysis revealed that the loss of hepatocyte RXRα leads to the significant downregulation of a cohort of essential niche-supportive factors (Bonnardel et al, 2019; Sakai et al, 2019; Tutusaus et al, 2020). This suggests that the KC depletion observed in the RXRα^iAlbKO^ liver is driven by the collapse of these hepatocyte-derived signals. Central to this process is the RXR/LXR heterodimer network. LXRs (LXRα and LXRβ) are highly expressed in KCs and control both lipid metabolism and inflammatory tone (Endo-Umeda et al, 2018; Scott et al, 2018; Bonnardel et al, 2019). Importantly, activation of LXR/RXR heterodimers has been shown to inhibit macrophage apoptosis and promote survival (Valledor et al, 2004), supporting a mechanistic link between disrupted RXR/LXR signaling and KC loss in our model. While direct lipidomic profiling was not performed in this study, our parallel-omics data provide a strong transcriptomic proxy for this hypothesized metabolic deprivation. Specifically, during sepsis, RXRα-deficient livers exhibit a profound suppression of the machinery required to synthesize and export LXR/RXR ligands, including major oxysterol synthesis enzymes (*Cyp27a1, Cyp46a1*), cholesterol transporters (*Abcg5* and *Abcg8*), and lipid/retinoid carriers *(Rbp4* and *Apoe*). However, we acknowledge that this deprivation of hepatocyte-derived metabolic support remains speculative in the absence of direct metabolic analyses. Future studies incorporating liver and/or Kupffer cell metabolomic profiling will be required to determine definitively whether altered hepatocyte-derived metabolic support functionally contributes to the impaired immune response observed in RXRα^iAlbKO^ mice during sepsis. Because LXR-RXR heterodimers depend on RXR for stable DNA binding and transcriptional activity, loss of RXRα in hepatocytes potentially alters hepatic lipid and retinoid metabolism in ways that reduce the availability of essential LXR/RXR ligands, such as oxysterols or retinoid derivatives, to resident macrophages. Our observation that sorted surviving KCs maintain normal *Rxra* expression in RXRα^iAlbKO^ mice, while the bulk tissue exhibits a collapse of the KC identity signature, reinforces this non-cell autonomous model. It suggests that KCs retain their own RXR but become deprived of hepatocyte-derived metabolic support required for tonic LXR/RXR activation. Insufficient LXR activity in KCs would be expected to diminish pro-survival, lipid-handling, and anti-inflammatory programs, thereby compromising niche stability and contributing to their depletion. This model is consistent with the observed reduction in KC numbers, impaired bacterial clearance, and the heightened inflammatory reprogramming that characterizes RXRα-deficient livers.

Importantly, the role of RXRα in sepsis appears to be highly cell-type specific, a distinction that becomes clear when comparing our findings with existing literature. While our data demonstrate that hepatocyte RXRα is protective and essential for maintaining KC populations and systemic antibacterial defense, Núñez et al, (2010) demonstrated that targeted deletion of RXRα in the myeloid compartment actually prolongs mouse survival in both CLP- and LPS-induced sepsis models. In their study, myeloid RXRα deficiency resulted in decreased macrophage production of the chemokines CCL6 and CCL9 (Núñez et al, 2010). This reduction impaired leukocyte recruitment to inflammatory sites, effectively protecting the mice from the detrimental hyper-inflammatory phase of sepsis (Núñez et al, 2010). This striking contrast highlights a highly compartmentalized function for RXR signaling during systemic infection: whereas myeloid RXRα acts as a driver of potentially lethal hyper-inflammatory leukocyte recruitment, hepatocyte RXRα functions as a critical immunometabolic hub required to prevent metabolic collapse and indirectly sustain local immune niches.

Although our multi-omics and genetic approaches converge on the HNF4α-RXRα-KC axis, a limitation of our study is the absence of a definitive “rescue” experiment (such as specifically preserving hepatocyte RXRα expression during infection) to establish absolute causal proof. Furthermore, translating these findings to human sepsis presents inherent challenges. Murine models cannot fully capture the immunological heterogeneity and complex comorbidities of human patients. Crucially, our mechanistic data derive primarily from an untreated CLP model, which lacks the human standard-of-care clinical interventions, like fluid resuscitation and broad-spectrum antibiotics, that define modern sepsis management.

However, our inclusion of a naturally acquired canine sepsis model demonstrates that RXRα loss-of-function is a conserved feature across species and also in a clinically relevant setting. This is further supported by human data from Dubois et al, 2020, showing that master liver-identity transcription factors (including key RXRα partners) are profoundly repressed in the livers of deceased sepsis patients (Dubois et al, 2020). Additionally, the prophylactic and early post-CLP efficacy of Bex observed here may be difficult to replicate clinically, as patients typically present with severe, established systemic inflammation. Overcoming this therapeutic hurdle likely requires finding and reverting the upstream cause of HNF4α loss-of-function. Stabilizing HNF4α could restore the RXRα signaling axis, potentially enabling therapeutic Bex administration, perhaps in combination with other ligands like PPARα that are also ‘victims’ of the HNF4α collapse. Future translational studies using humanized models or clinical datasets are therefore essential to evaluate the true therapeutic viability of targeting the HNF4α-RXRα axis.

In summary, our study demonstrates that hepatocyte RXRα is a critical node linking hepatic metabolic stability to systemic antimicrobial defense. We show that RXRα operates in an HNF4α-dependent cascade that is rapidly dismantled by septic inflammation, leading to a state of RXR resistance. We suggest a non-cell-autonomous role for hepatocyte RXRα in sustaining the KC population, the primary filter for systemic bacteremia. These findings highlight that restoring RXRα signaling axis early in the course of infection may provide a therapeutic window to preserve the hepatic “immunometabolic hub” and improve sepsis outcomes.

## Methods

**Table:** Reagents and tools table

Reagent/resource	Reference or source	Identifier or catalog number
**Experimental models**		
C57BL/6J (*M. musculus*)	Janvier	N/A
AlbCreERT2^Tg/+^: SA^+/CreERT2^ (*M. musculus*)	Prof. Daniel Metzger and Pierre Chambon (Igbmc, France)	N/A
B6.Rxra^tm1Krc^/J (*M. musculus*)	The Jackson Laboratory (Chen et al, 1998)	JAX stock number 013086
Clec4f-DTR (*M. musculus*)	Prof. Dr. M. Guilliams (Scott et al, 2016)	N/A
**Antibodies**		
Anti-RXRα	Abcam	AB_10975632
Anti-β-actin	Thermo Fisher Scientific	AB_10979409
Amersham ECL anti-mouse	GE Healthcare	AB_772210
Amersham ECL anti-rabbit	GE Healthcare	AB_772206
Anti-CD16/32	Bioceros	AB_3746267
CD45-eFluor 506	Thermo Fisher Scientific	AB_2637147
CD31-eFluor 450	Thermo Fisher Scientific	AB_10598807
F4/80-BV785	BioLegend	Cat# 123141
CD64-AF647	BioLegend	Cat# 305012
CLEC2-PeCy7	BioLegend	Cat# 146108
VSIG4-PE-eFluor 610	Thermo Fisher Scientific	AB_2802395
TIM-4 PE	BioLegend	AB_2201843
**Oligonucleotides and other sequence-based reagents**		
Gene	Forward primer (5’-3’)	Reverse (5’-3’)
*Rxra*	GATATCAAGCCGCCACTAGG	TTGCAGCCCTCACAACTGTA
*Gapdh*	TGAAGCAGGCATCTGAGGG	CGAAGGTGGAAGAGTGGGAG
*Hprt*	AGTGTTGGATACAGGCCAGAC	CGTGATTCAAATCCCTGAAGT
*Rpl*	CCTGCTGCTCTCAAGGTT	TGGTTGTCACTGCCTCGTACTT
**Chemicals, enzymes and other reagents**		
Metronidazole	Sigma-Aldrich	Cat# M-1547
Ceftriaxone	Sigma-Aldrich	Cat# C5793
Tamoxifen	Sigma-Aldrich	Cat# T5648
Bexarotene	Thermo Fisher Scientific	Cat# J63701.MC
**Software**		
GraphPad Prism v.11	GraphPad Software	SCR_002798
Enrichr	(Chen et al, 2013; Kuleshov et al, 2016; Xie et al, 2021)	SCR_001575
IPA	Ingenuity pathway analysis (Qiagen)	SCR_008653
qbase	qbase+ software (Biogazelle, Gent, Belgium)	SCR_003370
DESeq2	(Love et al, 2014)	SCR_015687
SpectroFlo	Cytek Biosciences	SCR_025494
FlowJo™ v10.8	BD Life Sciences	SCR_008520
STAR	(Dobin et al, 2013)	SCR_004463
featureCounts	(Liao et al, 2014)	SCR_012919
DIA-NN	(Demichev et al, 2020)	SCR_022865
**Other**		
Aurum total RNA mini kit	Bio-Rad	Cat# 732-6820
iScript cDNA synthesis kit	Bio-Rad	Cat# 170-8891
SensiFast SYBR No-Rox Kit	GeC Biotech	Cat# CSA-01190
WesternBright ECL kit	Advansta	Cat# K-12045-D20
Free Fatty Acid Assay kit	Abnova	Cat# KA1667
Bio-Plex Pro Mouse Cytokine Assay	Bio-Rad Laboratories	Cat# M60000007A
pHrodo Red dye	Thermo Scientific	Cat# P36600
Collagenase IV	Sigma-Aldrich	Cat# C4-BIOC
DNase I	Roche	Cat # 10104159001
Fixable Viability dye eFluor 780	Thermo Fisher Scientific	Cat # 65-0865-14
RNeasy Plus Micro Kit	Qiagen	Cat# 74034

### Mice

Male C57BL/6J mice were obtained from Janvier Labs. Liver-specific inducible RXRα-deficient mice were generated by crossing RXRα^fl/fl^ mice (strain B6.Rxra^tm1Krc^/J; JAX stock number 013086 (Chen et al, 1998); received from Dr. F. Dardel, Center Européen de Recherche en Biologie Moleculaire (CERBM GIE), France, under an MTA and by courtesy of Prof. Dr. M. Guilliams with Alb^CreERT2^Tg/+ mice (provided by Drs. D. Metzger & P. Chambon under an MTA). In this RXRα^fl/fl^ line, exon 4, which encodes the second zinc finger of the DNA-binding domain (DBD), was flanked by loxP sites via homologous recombination in embryonic stem (ES) cells (Wan et al, 2000; Sucov and Evans, 1994). KC-DTR mice (strain Clec4f-DTR; provided by Prof. Dr. M. Guilliams) were also used (Scott et al, 2016). For experiments utilizing wild-type (WT) mice (including CLP, sham, and Bex- and DMSO-treated cohorts), exclusively 8-week-old male mice were used (obtained from Janvier Labs). For experiments involving the transgenic RXRα^iAlbKO^ mice and their littermate controls, both male and female mice aged between 8 and 20 weeks were used from in-house breeding colonies. Mice were housed in specific pathogen-free IVC cages (22 °C, 14/10 h light/dark cycle) with ad libitum access to water and standard chow (18% protein, 4.5% fat; Provimi Kliba SA). All procedures complied with Ghent University ethics committee guidelines (EC2023-083).

Polymicrobial sepsis was induced via Cecal Ligation and Puncture (CLP) under isoflurane anesthesia (Rittirsch et al, 2009), utilizing a 75% cecal ligation and single puncture (21 G needle); sham mice underwent surgery without cecal injury. For the low-lethality CLP model, the procedure was modified by reducing the ligated portion of the cecum (to ~50%) to limit the necrotic burden and subsequent bacterial leakage. For survival studies, antibiotics (25 mg/kg ceftriaxone, 12.5 mg/kg metronidazole; IP) were administered 8 and 24 h post-CLP, with a humane endpoint of <28 °C body temperature. Animals utilized for short-term mechanistic analyses, tissue sampling, and bacterial counts (8 and 24 h endpoints) did not receive antibiotics or fluid resuscitation. In these tissue collection studies, no mice died prior to the planned sampling time points in the WT or Bex-treated cohorts. However, at the 24 h time point, four mice from the highly susceptible RXRα^iAlbKO^ group died prior to sample collection; these animals were not included in the downstream tissue analyses. Hepatic RXRα depletion was induced by IP tamoxifen (1 mg in 1:8 ethanol:oil) for 5 consecutive days, with experiments commencing 9 days post-treatment.

Mice were randomly allocated to experimental and control groups prior to the start of the procedures. Littermates were distributed across groups where possible to minimize confounding genetic or maternal effects. Data collection and downstream sample analyses were performed in a blinded manner where investigator bias could influence outcome assessment.

Pre-established exclusion criteria were applied to ensure data integrity. Animals were excluded from downstream analysis only in cases of severe procedural complications (such as excessive hemorrhaging during surgery) or if they met humane endpoints prior to the scheduled experimental timeline. No healthy, successfully treated animals were intentionally excluded from the final datasets.

The pan-RXR agonist Bexarotene (Bex) (Thermo Scientific) was administered IP at 10 mg/kg (in pure DMSO; 6 h after CLP) for the transcriptomics characterization experiment, or by oral gavage at 100 mg/kg (1/8 DMSO/corn oil; prophylactic and therapeutic combination: 48, 24, and 2 h before CLP, and 24 and 48 h post-CLP (as in (Cao et al, 2022); therapeutic 3, 24, and 48 h after CLP) for the protection experiments.

### Dogs

Dogs of various breeds with suspected or confirmed complicated intra-abdominal infection (cIAI) requiring a surgical procedure (laparotomy) were enrolled for the study at the Faculty of Veterinary Medicine, Merelbeke (see Dataset EV1). The septic group (*n* = 14) represented a diverse clinical population, including breeds such as German Shepherds, Labrador Retrievers, and Mixed Breeds. Diagnosis of sepsis was established based on the presence of at least two systemic inflammatory response syndrome (SIRS) criteria, including abnormalities in heart rate, respiratory rate, or white blood cell count, coupled with a confirmed intra-abdominal infectious focus identified during surgery or via preoperative cytology. The control group comprised non-septic dogs (*n* = 9) undergoing laparotomy for reasons other than sepsis (e.g., elective procedures such as ovariohysterectomy or non-infectious conditions like gastric foreign body removal without perforation). Dogs in the control group showed no clinical or biochemical evidence of systemic inflammation. During surgery, a liver biopsy (0.5 × 0.5 × 0.5 cm) was collected and preserved in RNAlater for further analysis. All procedures complied with Ghent University ethics committee guidelines (EC2022-073). All animal experiments were conducted and reported in accordance with the Animal Research: Reporting of In Vivo Experiments (ARRIVE) guidelines (Percie du Sert et al, 2020).

### Biochemical assays

Blood glucose and lactate levels were measured in tail blood with the use of, respectively, OneTouch Verio glucose meter (LifeScan) and Lactate Plus meter (Nova Biomedical).

### Cytokine quantification

Systemic inflammation was assessed by quantifying plasma cytokine concentrations (IL-2, IL-6, IL-10, IL-12, and TNFα) using a magnetic bead-based multiplex assay (Bio-Plex Pro Mouse Cytokine Assay, Bio-Rad Laboratories) according to the manufacturer’s instructions. Briefly, plasma samples were incubated with capture antibody-coupled magnetic beads in a black 96-well plate for 30 min at room temperature with orbital shaking (300 rpm). Following magnetic washing, samples were incubated with biotinylated detection antibodies for 30 min, followed by a 10-min incubation with streptavidin-phycoerythrin (PE). After a final wash, the beads were resuspended in assay buffer and fluorescence signals were acquired using a Bio-Plex reader system (Bio-Rad). Data were collected for a minimum of 50 beads per targeted analyte, and absolute concentrations (pg/mL) were determined from standard curves using a five-parameter logistic (5PL) regression model.

### Plasma organ damage assessment

Plasma markers of systemic organ dysfunction and tissue damage were quantified by the Clinical Biology Laboratory at Ghent University Hospital (UZ Gent). Plasma samples were analyzed for aspartate aminotransferase (AST), alanine aminotransferase (ALT), ureum, and bilirubin. Measurements were performed using automated clinical chemistry analyzers (Architect and Cobas platforms) according to standard accredited diagnostic laboratory procedures.

### Bacterial load quantification

Bacterial load in the liver, spleen and lungs was determined by homogenizing the organ in 1 mL sterile PBS. From this homogenate, 50 μL was plated out on a quarter of tryptic soy agar (TSA) plates. 10 μL of collected blood was plated out to determine systemic bacterial load. For time-course experiments 10 μl of blood was collected by making an incision on the tail of the same animal. Colony-forming units (CFUs) were counted 24 h post-inoculation at 37 °C, and CFU/mL was calculated.

### Gene expression analysis

Liver samples stored in RNAlater (Life Technologies Europe) were processed for total RNA extraction using the Aurum Total RNA Mini kit (Bio-Rad). Following quantification via Nanodrop 1000 (Thermo Scientific), 1 µg of RNA was converted to cDNA using the iScript cDNA Synthesis kit (Roche). The cDNA was diluted tenfold and analyzed by qPCR on a Light Cycler 480 (Roche) using Sensifast Bioline Mix (Bio-Line). Expression levels were normalized to *Gapdh*, *Hprt*, and/or *Rpl* using qbase+ software (Biogazelle), with samples run in duplicate.

### RNA-seq analysis

RNA quality control was performed using a Nanodrop 8000 (Thermo Scientific) and Agilent 5300 Fragment Analyzer. Libraries were prepared using the Illumina Stranded mRNA Ligation LP kit and sequenced (single-end, 100 bp) on either a NovaSeq 6000 or an Element Bio AVITI (VIB Nucleomics Core). Reads were mapped to the mm10 mouse genome using STAR (Dobin et al, 2013), and the count matrix was created with featureCounts (Liao et al, 2014). DESeq2 (Love et al, 2014) was used for differential expression analysis (FDR <0.05), including only uniquely mapped reads. Downstream analysis utilized Enrichr (Chen et al, 2013; Kuleshov et al, 2016; Xie et al, 2021), and ingenuity pathway analysis (IPA) (Qiagen). For RNA-seq processing details, see (Vandewalle et al, 2021).

The RNA-seq analysis of mice CLP at 8 h time point was performed from a published and publicly available dataset (GEO GSE139484) (Van Wyngene et al, 2020). HNF4α^iAlbKO^ mice RNA-seq analysis was performed from a published and publicly available dataset (GEO GSE260635) (Van Dender et al, 2024). The RNA-seq analysis of FACS-sorted Kupffer cells from WT mice 8 h post-sham or CLP surgery was performed using a publicly available dataset (GEO GSE311736).

### Western blotting

Total protein was extracted from snap-frozen liver tissue using RIPA lysis buffer containing a protease inhibitor cocktail (Roche), with concentrations quantified via Bradford assay. Thirty micrograms of protein were resolved on an 8% SDS-polyacrylamide gel and transferred to a 0.45-μm nitrocellulose membrane. Following blocking with diluted Starting Block/0.1% PBST (Thermo Fisher Scientific), membranes were incubated overnight at 4 °C with primary antibodies against RXRα (Abcam; AB_10975632; 1:1000), using β-actin (Thermo Fisher Scientific; AB_10979409; 1:5000) as a loading control. Membranes were washed with 0.1% PBST and incubated for 1 h at room temperature with HRP-conjugated Amersham ECL anti-mouse (AB_772210; 1:5000) or anti-rabbit (AB_772206; 1:5000) antibodies (GE Healthcare). Protein bands were visualized using the WesternBright Quantum kit (Advansta) and quantified on an Amersham Imager 600 (GE Healthcare).

### Nuclear fractionation protein extraction

Nuclear lysates were prepared from 50 mg of snap-frozen liver tissue. Following homogenization in PBS supplemented with protease inhibitors (Roche), nuclei were isolated using a buffer containing 0.5% NP-40 and 0.25% Triton X-100. The nuclear pellet was washed and solubilized in lysis buffer (0.5% *N*-lauroylsarcosine, 0.1% Na-deoxycholate) containing protease inhibitors. Samples were sonicated for 15 min using a PIXUL Multisample sonicator (Active Motif) with default parameters (Pulse 50 cycles, PRF 1 kHz, burst rate 20 Hz), and protein concentrations were quantified via Bradford assay.

### LC-MS/MS and proteomic data analysis

Peptides were resuspended in loading solvent A (0.1% TFA, 0.5% ACN) and 2 μl were injected onto an Ultimate 3000 ProFlow nanoLC system coupled to a Q Exactive HF mass spectrometer (Thermo Fisher Scientific) featuring a pneu-Nimbus dual ion source (Phoenix S&T). Samples were trapped on a PepMap™ Neo column (Thermo; 300 μm I.D., 5-μm beads) at 20 μl/min for 2 min, then separated on a 45 °C thermostated Odyssey Ultimate C18 column (IonOpticks; 250 mm, 1.7 μm, 75 μm I.D.). Peptides were eluted at 250 nl/min using a gradient of solvent B (0.1% FA in ACN), reaching 26.4% at 75 min, 44% at 95 min, and 56% at 100 min. The mass spectrometer operated in data-independent acquisition (DIA) mode, alternating full MS scans (375–1500 m/z; 60000 resolution; AGC 5 × 10⁶; max IT 50 ms) with 30 HCD fragmentations (isolation width 10 m/z; NCE 30%; AGC 3 × 10⁶; max IT 45 ms; 15,000 resolution). Isolation windows (400–900 m/z) were generated via Skyline, and lock mass 445.120028 was used for internal calibration. Instrument performance was monitored via QCloud (Chiva et al, 2018; Olivella et al, 2021).

Raw data were processed using DIA-NN (v1.9.1) (Demichev et al, 2020) against the UniProt Mus musculus database (June, 2024; UP000000589) with a 1% precursor FDR. Search settings included trypsin specificity (max 1 missed cleavage), fixed cysteine carbamidomethylation, variable methionine oxidation and N-terminal acetylation, and “Match between runs” enabled. Mass and scan window tolerances were automatically determined by DIA-NN (Demichev et al, 2020). Subsequent analysis in R utilized the QFeatures package to filter for proteotypic peptides and a 1% *q*-value cutoff. Protein group MaxLFQ intensities were log_2_-transformed. After filtering for proteins with at least one valid value in at least one group, missing values were imputed using the “min” method within the DEP package (Zhang et al, 2018). Differential expression was determined using limma (Ritchie et al, 2015), with significance defined as adjusted *p* value <0.05 and |log2FC| ≥ 0.58. Functional characterization of differentially expressed proteins was performed via Gene Ontology (GO) and KEGG pathway enrichment analysis using the clusterProfiler package.

### Phagocytosis assay and KC sorting

For the phagocytosis assay and Kupffer cell (KC) sorting, E. coli (K12 MG1655, BCCM) were cultured in Luria Broth (LB) medium at 37 °C and 5% CO_2_ for 9 h. Bacteria were subsequently collected via centrifugation at 3220 × *g* for 5 min, resuspended in 100 mM sodium bicarbonate (pH 8.5), and conjugated with pHrodo Red dye (Thermo Scientific) for 45 min at room temperature in the dark. To remove residual dye, the suspension was washed with HBSS and fixed in 100% methanol prior to final resuspension in HBSS for intravenous delivery. Following a 1.5-h period for in vivo phagocytosis, liver cells were isolated using a modified perfusion-digestion technique. Livers were subjected to retrograde cannulation and flushed for 2 min with an EDTA-HBSS solution, followed by a 5-min perfusion (6 ml/min) with 0.2 mg/ml Collagenase IV (Sigma-Aldrich). Excised livers were minced and further processed in a 20-min incubation with 0.4 mg/ml Collagenase IV and 10 U/ml DNase I (Roche) at 37 °C. All subsequent handling was performed at 4 °C to ensure cell stability. The resulting suspension was filtered through a 100 µm mesh, subjected to osmotic erythrocyte lysis, and passed through a 40-µm filter. To deplete hepatocytes and debris, the cells were centrifuged twice at 50 × *g* (1 min) before the non-parenchymal fraction was recovered at 400 × *g* for 5 min. For spectral flow cytometry, ~1–5 × 10⁶ cells were incubated with anti-CD16/32 (1:100; clone 2.4G2; Bioceros; AB_3746267) to block Fc receptors and stained for 30–45 min at 4 °C. The antibody panel included: Fixable Viability dye eFluor 780 (1:500; Thermo Fisher; 65-0865-14), CD45-eFluor 506 (1:200; Thermo Fisher; AB_2637147), CD31-eFluor 450 (1:500; Thermo Fisher; AB_10598807), F4/80-BV785 (1:200; BioLegend; 123141), CD64-AF647 (1:200; BioLegend; 305012), CLEC2-PeCy7 (1:400; BioLegend; 146108), and VSIG4-PE-eFluor 610 (1:400; Thermo Fisher; AB_2802395), was based on (Ponti, 2025). TIM-4 PE was omitted from the pHrodo phagocytosis panel due to spectral overlap with the pHrodo Red signal. Data were collected on a Cytek Aurora spectral flow cytometer. Data acquisition was performed using SpectroFlo software (Cytek Biosciences), and downstream gating analysis was executed using FlowJo™ v10.8 Software (BD Life Sciences). Phagocytic activity was determined by the mean fluorescence intensity (MFI) of the pHrodo Red signal within the live CD45+, CD31–, F4/80+, CLEC2+, CD64+, VSIG4 + KC population. For KC sorting, the panel was supplemented with TIM-4 PE (1:200; BioLegend; AB_2201843). KCs (50000 cells) were isolated using a BD FACS Discover S8 directly into tubes containing sort buffer (HBSS with 2% FCS and 2 mM EDTA). Post-sort, cells were pelleted (400×*g*, 7 min, 4 °C), and the resulting pellet was lysed in 300 μl RLT buffer with 1% β-mercaptoethanol. Samples were snap-frozen and stored at -80 °C until RNA extraction via the RNeasy Plus Micro Kit (Qiagen).

### Statistical analysis

To ensure normal distribution, qPCR data, bacterial loads (CFU/mL), and plasma cytokine levels were log-transformed. Normality was verified using GraphPad Prism 10.0 (GraphPad Software, Inc.), which was also utilized for data visualization. Comparisons between two group means were performed using an unpaired Student’s *t*-test; Welch’s correction was applied in cases of significantly different standard deviations (SD). For comparisons involving more than two groups, a one-way ANOVA followed by Dunnett’s or Tukey’s multiple comparisons test was conducted. Analyses involving a second variable utilized a two-way ANOVA with Tukey’s or Sidak’s post hoc tests. Kaplan–Meier survival curves were assessed using the log-rank, Gehan–Breslow–Wilcoxon, or one-sided chi-squared tests. Statistical significance was defined as *P* < 0.05 (ns: not significant). Sample size (*n*) denotes the number of biological replicates, which was determined based on prior experiments. All experiments presented in the main figures were replicated at least twice. Data were displayed as bar plots representing the mean ± SD. Specific statistical details are provided in the figures or figure legends. To minimize confounding factors, treatment groups were balanced for age and sex during allocation, and treatments were distributed across different cages. All data analyses were performed blindly to prevent subjective bias.

## Supplementary information

Peer Review File

Dataset EV1

Source data Fig. 1

Source data Fig. 2

Source data Fig. 3

Source data Fig. 4

Source data Fig. 5

Expanded View Figures

## Data Availability

RNA-Seq data have been deposited at the National Center for Biotechnology Information Gene Expression Omnibus (GEO) public database (http://www.ncbi.nlm.nih.gov/geo/) and are publicly available under accession numbers GSE319754 (BEX CLP/sham), GSE319852 (RXRα^iAlbKO^ in CLP/sham), GSE334778 (Dog naturally acquired septic peritonitis). Publicly available RNA-sequencing datasets used in this study are accessible through GEO under accession numbers GSE139484 (WT CLP/sham), GSE260635 (HNF4α^iAlbKO^ in CLP/sham) and GSE311736 (sorted KCs upon CLP/sham). The source data of this paper are collected in the following database record: biostudies:S-SCDT-10_1038-S44321-026-00480-y.
