# Supplementary material for: RXRα suppression drives hepatic metabolic and immune dysfunction in sepsis
Source: EMBO Mol Med. 2026 Jul 16;18(8):3318–44. doi: 10.1038/s44321-026-00480-y (PMC13469149; doi:10.1038/s44321-026-00480-y)
Supplement: Supplementary file 1 [file 44321_2026_480_MOESM1_ESM.pdf]

EMBO Molecular Medicine

RXRα Suppression Drives Hepatic Metabolic and Immune Dysfunction in Sepsis

Matyas Jelinek, Jolien Vandewalle, Marah Heyerick, Tineke Vanderhaeghen, Céline Van Dender, Steven Timmermanns, Madeleine Hellemans, Daria Fijalkowska, Hilde De Rooster, Martin Guilliams, Karolien De Bosscher, and Claude Libert

Corresponding author: Claude Libert (jclaude.libert@irc.vib-ugent.be)

Review Timeline:

Submission Date:	17th Feb 26
Editorial Decision:	6th Mar 26
Revision Received:	2nd Jun 26
Editorial Decision:	10th Jun 26
Revision Received:	23rd Jun 26
Accepted:	24th Jun 26

Editor: Zeljko Durdevic

Transaction Report:

(Note: With the exception of the correction of typographical or spelling errors that could be a source of ambiguity, letters and reports are not edited. Depending on transfer agreements, referee reports obtained elsewhere may or may not be included in this compilation. Referee reports are anonymous unless the Referee chooses to sign their reports.)

6th Mar 2026

Dear Dr. Vandewalle,

Thank you for the submission of your manuscript to EMBO Molecular Medicine. We have now received feedback from the three reviewers who agreed to evaluate your manuscript. All three referees recognize interest of the study but also raise importnat concerns that should be addressed in a major revision. If you would like to discuss further the points raised by the referees, I am available to do so via email or video. Let me know if you are interested in this option.

Further consideration of a revision that addresses reviewers' concerns in full will entail a second round of review. EMBO Molecular Medicine encourages a single round of revision only and therefore, acceptance or rejection of the manuscript will depend on the completeness of your responses included in the next, final version of the manuscript. For this reason, and to save you from any frustrations in the end, I would strongly advise against returning an incomplete revision.

We would welcome the submission of a revised version within three months for further consideration. Please let us know if you require longer to complete the revision.

I look forward to receiving your revised manuscript.

Yours sincerely,

Zeljko Durdevic

Zeljko Durdevic
Senior Editor
EMBO Molecular Medicine

*****
When preparing your revised manuscript, please refer to our guidelines: https://link.springer.com/journal/44321/submission-guidelines#cms-Revised-submissions. We perform an initial quality control of all revised manuscripts before re-review; failure to include requested items will delay the evaluation of your revision.

We require:

1) A .docx formatted version of the manuscript text (including legends for main figures, EV figures and tables). Please make sure that the changes are highlighted to be clearly visible.

2) Individual production quality figure files as .eps, .tif, .jpg (one file per figure). For guidance, download the 'Figure Guide PDF': https://media.springernature.com/original/springer-cms/rest/v1/content/27825798/data/v1.

3) A .docx formatted letter INCLUDING the reviewers' reports and your detailed point-by-point responses to their comments. As part of the EMBO Press transparent editorial process, the point-by-point response is part of the Review Process File (RPF), which will be published alongside your paper.

4) A complete author checklist, which you can download from our author guidelines. Please insert information in the checklist that is also reflected in the manuscript. The completed author checklist will also be part of the RPF.

5) Please note that all corresponding authors are required to supply an ORCID ID for their name upon submission of a revised manuscript.

6) It is mandatory to include a 'Data Availability' section after the Materials and Methods. Before submitting your revision, primary datasets produced in this study need to be deposited in an appropriate public database, and the accession numbers and database listed under 'Data Availability'. Please remember to provide a reviewer password if the datasets are not yet public.

In case you have no data that requires deposition in a public database, please state so in this section. Note that the Data Availability Section is restricted to new primary data that are part of this study.

7) For data quantification: please specify the name of the statistical test used to generate error bars and P values, the number (n) of independent experiments (specify technical or biological replicates) underlying each data point and the test used to calculate p-values in each figure legend. The figure legends should contain a basic description of n, P and the test applied. Graphs must include a description of the bars and the error bars (s.d., s.e.m.).

8) At EMBO Press we ask authors to provide source data for the main manuscript figures. You will receive a separate email with instructions for providing source data with your revised manuscript, including how to upload and organize the files.

9) Our journal encourages inclusion of *data citations in the reference list* to directly cite datasets that were re-used and obtained from public databases. Data citations in the article text are distinct from normal bibliographical citations and should directly link to the database records from which the data can be accessed. In the main text, data citations are formatted as follows: "Data ref: Smith et al, 2001" or "Data ref: NCBI Sequence Read Archive PRJNA342805, 2017". In the Reference list, data citations must be labeled with "[DATASET]". A data reference must provide the database name, accession number/identifiers and a resolvable link to the landing page from which the data can be accessed at the end of the reference.

10) We replaced Supplementary Information with Expanded View (EV) Figures and Tables that are collapsible/expandable online. A maximum of 5 EV Figures can be typeset. EV Figures should be cited as 'Figure EV1, Figure EV2" etc... in the text and their respective legends should be included in the main text after the legends of regular figures.

- For the figures that you do NOT wish to display as Expanded View figures, they should be bundled together with their legends in a single PDF file called *Appendix*, which should start with a short Table of Content. Appendix figures should be referred to in the main text as: "Appendix Figure S1, Appendix Figure S2" etc.

- Additional Tables/Datasets should be labeled and referred to as Table EV1, Dataset EV1, etc. Legends have to be provided in a separate tab in case of .xls files. Alternatively, the legend can be supplied as a separate text file (README) and zipped together with the Table/Dataset file.

11) The paper explained: EMBO Molecular Medicine articles are accompanied by a summary of the articles to emphasize the major findings in the paper and their medical implications for the non-specialist reader. Please provide a draft summary of your article highlighting

- the medical issue you are addressing,

- the results obtained and

- their clinical impact.

This may be edited to ensure that readers understand the significance and context of the research. Please refer to any of our published articles for an example.

12) Author contributions: You will be asked to provide CRediT (Contributor Role Taxonomy) terms in the submission system. These replace a narrative author contribution section in the manuscript.

13) A Conflict of Interest statement should be provided in the main text.

14) Every published paper includes a 'Synopsis' to further enhance discoverability. Synopses are displayed on the journal webpage and are freely accessible to all readers. They include a short stand first (maximum of 300 characters, including space) as well as 2-5 one-sentences bullet points that summarizes the paper. Please write the bullet points to summarize the key NEW findings. They should be designed to be complementary to the abstract - i.e. not repeat the same text. We encourage inclusion of key acronyms and quantitative information (maximum of 30 words / bullet point). Please use the passive voice. Please attach these in a separate file or send them by email, we will incorporate them accordingly.

Please also suggest a striking image or visual abstract to illustrate your article as a PNG file 550 px wide x 300-600 px high.

15) Include a Reagents and Tools Table as part of the Methods section, which can be downloaded from our author guidelines.

EMBO Molecular Medicine has a "scooping protection" policy, whereby similar findings that are published by others during review or revision are not a criterion for rejection. Should you decide to submit a revised version, I do ask that you get in touch after three months if you have not completed it, to update us on the status.

***** Reviewer's comments *****

Referee #1 (Comments on Novelty/Model System for Author):

In this paper Jelinek et al present data showing that in sepsis conditions induced by cecal ligation and puncture (CLP), RXRa expression levels in hepatocytes decrease, which leads to transcriptome changes affecting sepsis-mediated tissue tolerance. Pre-treatment of mice with RXRa agonist, bexarotene, improved survival and reduced bacterial load in four different organs. Using hepatocyte-specific RXRa-KO mouse model, the authors show that the protective effect of RXRa agonist is mediated through hepatocyte-expressed RXRa. The authors also show that hepatocyte-specific RXRa inactivation results in Kupffer cell depletion.
The findings of this paper are interesting, and in light of previous publications of the laboratory on sepsis induced decrease of PPARa and HNF4 functions, they are not unexpected. This is because HNF4 is an upstream regulator of RXRa and PPARa requires heterodimerization with RXRa to bind to its targets. The important novel finding of the paper is that pharmacological targeting of RXRa can have protective effect on sepsis-mediated tissue damage.
The results as presented raise the following questions and concerns:

1) The mechanism of Bexarotene is not clear. Bex can efficiently activate RXRa/PPARa, RXRa/FXRa, RXRa/LXR heterodimers but not RXRa/RXRa homodimers and to a lesser extent RXRa/RaR heterodimers. The authors could clarify this by comparing the effects on the transcriptomes modulated by PPARa, FXR and LXR agonists and by the analysis of existing or new ChIP-seq data with PPARa, FXR and LXR antibodies. Comparing binding profiles with RNA level changes would provide information about functional interactions.

2) The results of this study and those in HNF4 and PPAR study point to an important role of hepatocyte metabolic state in systemic antimicrobial defense. The authors provide overall survival curves body temperature, glucose, lactate levels and bacterial loads in the different in different organs (e.g., in Figure 3). Some information e.g., histological evaluation of damage in liver and other tissues in the different treatment conditions and cytokine profiling should be included to assess how Bex treatment and hepatic RXRa expression influence systemic effects.

3) The role of hepatic RXRa in the expression of hepatic niche factors, like Tgfb, Bmp6 and Csf1, which support Kupffer cell maintenance is an interesting finding. The heat bar in Figure 5J should be replaced with a correct one! It should be indicated if the colors indicate fold changes or z-scores.
Are these genes also downregulated in HNF4-KO, PPARa-KO, LXRa-KO or FXRa-KO livers? Is it known if Kupffer cells are depleted in the above models?
The decreased expression of Kupffer cell core genes in Fig S5B and D was detected using RNA preps from whole liver. Therefore, these data simply reflect the reduced number of Kupffer cells in RXRa-KO livers and in wild type livers following CLP. It would be interesting to compare Kupffer cell transcriptomes after FACS sorting in wild type livers and in cells remaining in CLP livers to evaluate effects independent of hepatic niche perturbation. Measurements of the total number of Kupffer cells and the number of phagocytosing cells in CLP condition and in conditions after treatment with Bex should be included.

4) The data in Fig5 E are important, because they show that the phagocytosis potential of the Kupffer cells is actually increased, which reflects possible compensatory effect and that the main mechanism of immune dysfunction in RXR-KO mice is due to the reduced number of Kupffer cells. Representative spectral flow cytometry images should be shown next to the bar graphs.

Referee #2 (Comments on Novelty/Model System for Author):

The manuscript contains some interesting and novel findings but is insufficiently discussing limitations and also pre-existing knowledge in the field including own data by the group.
In the reviewer's opinion, the manuscript will only be suitable for publication after a thorough, (self-)critical review and discussion.

Referee #2 (Remarks for Author):

The study by Jelinek et al. is using pharmacological and genetic approaches to explore immune functions of the hepatocyte retinoid receptor α (RXRα) in a mouse model of polymicrobial sepsis. The data of the authors reveal suppression of RXR signaling during sepsis progression and a protective role of the RXR agoinist bexarotene on sepsis induced mortality. Hepatocyte specific RXR deficiency was shown to provoke bacteremia involving a loss of undefined vitalizing effects of

hepatocytes on liver resident myeloid Kupffer cells.
These data are new and significantly deepen the insights in the complex regulatory functions and mechanisms of RXR in the mouse liver. Nevertheless, some aspects need attention:

Major comments
The integration of the authors' experimental data into the current state of knowledge regarding the role of RXR receptors in sepsis-relevant processes is consistently inadequate.
As a prime example the study by Nunez et al. (PNAS 2010) might be mentioned, which, contrary to the authors results, shows reduced mortality in a myeloid RXRalpha knockout model. Whereas the decreased chemokine in myeloid ko mice has been mentioned in the discussion part of the present manuscript the striking result of lower susceptibility of these mice to sepsis has been ignored.
Similar shortcomings exist in the presentation of the medical relevance of the results. Mouse models are useful, but due to the animals' lifestyle and lifespan, they can only provide limited information regarding clinically applicable therapeutic approaches.This needs to be addressed via translational material/data or at least clearly be stated.

Referee #3 (Remarks for Author):

This is a very elegant study, using different experimental approaches to investigate the role of RXRalpha suppression in sepsis. The results and methodological setup requires however some clarification, as the design or rationale is not always clear.
Specific comments:
1.The introduction could use some citations other than the own work of the authors. E.g. PMID: 30677458 important role for PPARalpha, PMID: 32407006 on HNF4alpha and NR
2.It should be better clarified in the text and figure legends that the first part of the results (Figure 1A-G) are collected from previous studies from the same authors. Also, n=3 is quite a low number for transcriptomics analysis?
3.In the first analysis (Fig 1D) it is stated 'prioritizing metabolic dysfunction over the more extensively characterized inflammatory response" what do you mean by that? How was the analysis done? Did the authors selectively excluded specific pathways? Was the same methodology used in the following signaling pathways analyses 5Fig 1G, Fig 1J, Fig 2D, Fig 3D?
4.In the first BEX treatment experiment, what was the rationale to give the injection 6h post CLP/sham?
5.Why is the number of animals in the survival analysis in Fig3I different between groups? With only n=5 in the 2 KO groups, it is not surprising that no statistical difference was reached. Was a power calculation performed beforehand? In the follow-up experiment (Fig4 B) 15/16 animals per group were tested; What is the rationale to these different numbers?
6.Information on the CLP procedures is to limited. In the methods it is stated that only animals in the survival studies received antibiotics. Did none of the animals on which tissues analyses were performed receive fluids or antibiotics? How many animals died in the non-survival studies with end-points at 8h, or 24h? I am asking because with such a high lethality model, and no treatment , you might have created some bias by only selecting animals that survived the CLP until the time of sampling?
7.Please explain how the "low-lethality" was reached in the exp of Fig4A. What is the age and sex of the animals per experiment?
8.The authors suggest a deprivation of hepatocyte-derived metabolic support to the Kuppfer Cells, but this remains very speculative. Any information on the metabolic signature in the CLP RXR KO mice?
9.Although the story includes a lot of different interventions, there is no 'rescue' experiment that ties the different aspects together. Furthermore, these findings in an untreated CLP mouse model cannot easily be extrapolated to a therapeutic intervention for humans. The translation from mice to men has failed more than it has succeeded. These limitations need to be discussed.

Referee #1 (Comments on Novelty/Model System for Author):

In this paper Jelinek et al present data showing that in sepsis conditions induced by cecal ligation and puncture (CLP), RXRa expression levels in hepatocytes decrease, which leads to transcriptome changes affecting sepsis-mediated tissue tolerance. Pre-treatment of mice with RXRa agonist, bexarotene, improved survival and reduced bacterial load in four different organs. Using hepatocyte-specific RXRa-KO mouse model, the authors show that the protective effect of RXRa agonist is mediated through hepatocyte-expressed RXRa. The authors also show that hepatocyte-specific RXRa inactivation results in Kupffer cell depletion.

The findings of this paper are interesting, and in light of previous publications of the laboratory on sepsis induced decrease of PPARa and HNF4 functions, they are not unexpected. This is because HNF4 is an upstream regulator of RXRa and PPARa requires heterodimerization with RXRa to bind to its targets. The important novel finding of the paper is that pharmacological targeting of RXRa can have protective effect on sepsis-mediated tissue damage.

We thank the reviewer for the positive and constructive feedback. We have carefully addressed all comments raised by the reviewer, and the corresponding changes incorporated into the revised manuscript are highlighted in yellow.

The results as presented raise the following questions and concerns:

1) The mechanism of Bexarotene is not clear. Bex can efficiently activate RXRa/PPARa, RXRa/FXRa, RXRa/LXR heterodimers but not RXRa/RXRa homodimers and to a lesser extent RXRa/RaR heterodimers. The authors could clarify this by comparing the effects on the transcriptomes modulated by PPARa, FXR and LXR agonists and by the analysis of existing or new ChIP-seq data with PPARa, FXR and LXR antibodies. Comparing binding profiles with RNA level changes would provide information about functional interactions.

We agree that comparing the transcriptomic and ChIP-seq profiles of PPARa, FXR, and LXR elegantly clarifies how Bex preferentially activates permissive heterodimers over RXR homodimers. We will update the manuscript with the following evidence to clarify this mechanism:

- Genomic Co-occupation (Binding Profiles): Boergesen et al. demonstrated extensive overlap in the genome-wide binding profiles of these receptors. In mouse liver, PPARa binds to 71–88% of shared LXR-RXR sites, and there are ~2,000 genomic "hot spots" co-occupied by LXR, RXR, PPARa, and FXR. Bex specifically increases RXR recruitment to these shared sites, confirming that these permissive heterodimers functionally interact and share degenerate response elements.
- Ligand-Induced Dimerization Switch (RNA/Transcriptome Changes): Menendez-Gutierrez et al. (2015) provided direct evidence of this shift. Under basal conditions, RXR operates as a homodimer to directly regulate specific genes (e.g., Mafb). However, pharmacological activation by rexinoids actively shifts RXR away from homodimerization, forcing the formation of RXR/LXR heterodimers that drive an entirely different transcriptomic program (e.g., SREBP-1c induction).
- Permissive Activation: As reviewed by Menendez-Gutierrez and Ricote (2017), RXR/PPAR, RXR/LXR, and RXR/FXR are "permissive" heterodimers, meaning they can be fully activated by an RXR ligand alone. Bex powerfully exploits this permissivity, hijacking RXR to trigger broad systemic responses through these heterodimer partners rather than the more limited basal homodimer pathways.

We have incorporated this synthesis of binding profiles and RNA-level changes into the revised text to explicitly address the functional interactions of these receptors upon Bex treatment.

"Furthermore, genome-wide and transcriptomic analyses demonstrate that pharmacological RXR agonism actively diverts the receptor from its basal homodimeric state to form permissive heterodimers with partners such as PPARα, LXR, and FXR (Menéndez-Gutiérrez et al., 2015; Menéndez-Gutiérrez and Ricote, 2017). In the liver, Bex strongly increases RXR recruitment to shared genomic response elements extensively co-occupied by these heterodimer partners, driving distinct transcriptional programs that regulate metabolism (Boergesen et al., 2012). This ligand-induced shift, which heavily favors heterodimer-driven pathways over RXR homodimers, provides a mechanistic basis for the metabolic stabilization observed here. The loss of RXRα responsiveness once systemic inflammation and HNF4α reduction are established explains why Bex was protective only when administration started before CLP, whereas therapeutic treatment conferred no survival benefit."

2) The results of this study and those in HNF4 and PPAR study point to an important role of hepatocyte metabolic state in systemic antimicrobial defense. The authors provide overall survival curves body temperature, glucose, lactate levels and bacterial loads in the different in different organs (e.g., in Figure 3). Some information e.g., histological evaluation of damage in liver and other tissues in the different treatment conditions and cytokine profiling should be included to assess how Bex treatment and hepatic RXRa expression influence systemic effects.

We thank the reviewer for highlighting the importance of assessing the systemic inflammatory and organ damage landscape. We agree that this information is supportive for contextualizing the protective effects of Bex and the role of hepatic RXRα. To address this, we have performed additional profiling;

- To assess cytokine profiling, we performed Bioplex cytokine analysis on plasma collected from mice treated with vehicle or Bex 24 hours post-CLP. Consistent with the reduced bacterial burden and enhanced Kupffer cell phagocytosis observed in these animals, Bex treatment resulted in significantly lower levels of circulating cytokines. We have included the core pro- and anti-inflammatory mediators (IL-6, IL-10, and TNFα) in the main text (Figure 3F) and additional relevant cytokines (IL-2 and IL-12) in the supplement (Supplementary Figure S3A). Rather than indicating a direct immunosuppressive effect, these attenuated cytokine profiles likely reflect a consequence of improved systemic bacterial clearance, correlating with the lower overall disease severity and preserved metabolic status in the BEX-treated group.

  *"In addition to improved survival, Bex-treated animals maintained significantly higher body temperature (**Figure 3C**) and plasma glucose levels 24 hours post-surgery (**Figure 3D**), suggesting protection against the hypothermia and hypoglycemia typically observed in severe sepsis. Plasma lactate concentrations were markedly reduced (**Figure 3E**). Consistent with this improved metabolic stability, systemic inflammation was attenuated, as reflected by lower circulating IL-2, IL-6, IL-10, IL-12 and TNFα levels, 24h post-surgery (**Figure 3F** and Supplementary figure S3A)."*

- To assess tissue damage, we performed preliminary histological assessments of the liver; however, at this relatively early 24-hour post-CLP time point, standard H&E staining did not yet reveal overt structural damage in the CLP mice treated with vehicle. As our data suggests, the primary pathology at this stage is driven by a functional, immunometabolic collapse rather than gross structural disintegration. To provide a more sensitive, quantitative, and objective assessment of systemic organ dysfunction, we measured a comprehensive panel of plasma damage markers. This panel evaluates the functional integrity of the liver (AST, ALT, Bilirubin, GGT), kidneys (Ureum, Creatinine), heart (Troponin T), and general tissue/muscle (LDH, CK) (EV1). We are providing the graphs for the entire panel here for the reviewer.

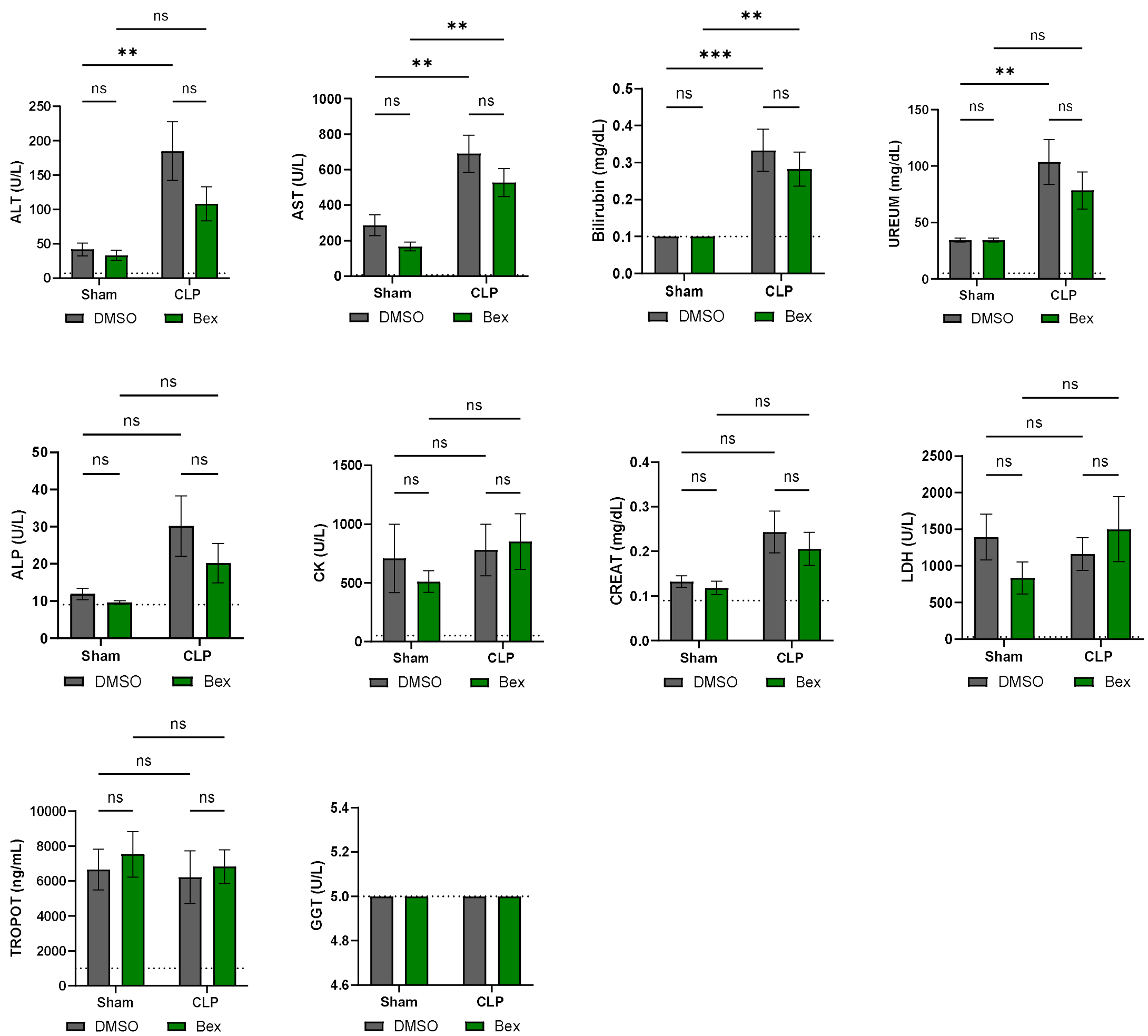

EV1: Quantification of a comprehensive panel of plasma damage markers to assess systemic organ dysfunction 24 hours post-sham or CLP surgery in mice treated prophylactically with vehicle (DMSO) or Bex. The panel evaluates the functional integrity of the liver (ALT, AST, Bilirubin, ALP, GGT), kidneys (UREUM, CREAT), heart (TROPOT), and general tissue/skeletal muscle (CK, LDH). Dashed horizontal lines indicate the limit of detection (LOD) for each respective biochemical assay. Data are represented as mean ± SD. Statistical significance was determined by two-way ANOVA with Tukey's multiple comparisons test; $p < 0.01$, *** $p < 0.001$, ns = not significant.

This analysis revealed a significant CLP-induced increase in the liver-specific injury markers AST, ALT, and Bilirubin, as well as the renal marker Ureum, in the control (DMSO) group. Importantly, this CLP-induced increase was not statistically significant for ALT and Ureum in the Bex-treated group when compared to their respective sham controls. While the direct comparison between the DMSO-CLP and Bex-CLP groups did not reach statistical significance, a downward trend was consistently observed in the Bex-treated animals. The fact that these prominent downward trends were observed in the hepatic markers (AST, ALT, and Bilirubin) provides an independent argument emphasizing the critical role of the liver in this sepsis model. We have now included these specific markers (AST, ALT, Bilirubin, and Ureum) in the manuscript (Supplementary Figure S3B) and discussed how Bex influences systemic organ integrity in the Results section.

"To further evaluate the impact of Bex treatment on sepsis-induced organ dysfunction, we quantified a comprehensive panel of plasma damage markers. Polymicrobial sepsis induced a significant elevation in liver-specific injury markers (AST, ALT, and Bilirubin) as well as the renal marker Ureum in DMSO-treated controls (Supplementary Figure S3B). In contrast, the CLP-induced increase for ALT and Ureum was not statistically significant in the Bex-treated group compared to sham controls. While the direct reduction between vehicle- and Bex-treated septic mice did not reach statistical significance, a consistent downward trend was observed for these parameters, indicating that early RXRα activation might limit the trajectory of systemic organ damage and preserves hepatic integrity during the acute phase of infection."

3a) The role of hepatic RXRa in the expression of hepatic niche factors, like Tgfb, Bmp6 and Csf1, which support Kupffer cell maintenance is an interesting finding. The heat bar in Figure 5J should be replaced with a correct one! It should be indicated if the colors indicate fold changes or z-scores.

We apologize for the oversight in Figure 5J. We have updated the figure and the accompanying legend to explicitly state that the colours represent Log2 Fold Changes (LFC).

3b) Are these genes also downregulated in HNF4-KO, PPARa-KO, LXRa-KO or FXRa-KO livers? Is it known if Kupffer cells are depleted in the above models?

The depletion of niche factors and general KC depletion in other knockout models is a highly relevant question to determine whether this niche collapse is specific to the RXRα hub or a general feature of knocking out any nuclear receptor in hepatocytes. To answer this, we analyzed our own published HNF4α-KO dataset and re-analyzed publicly available RNA-seq datasets for hepatocyte-specific PPARα, LXR, and FXR knockouts;

HNF4α (GSE260635): Because HNF4α is the direct upstream driver of RXRα, we hypothesized it would share this phenotype. Indeed, our published $HNF4\alpha^{iAlbKO}$ dataset showed a significant downregulation of both the essential niche-supportive signals and the KC-core identity signature (indicating KC depletion). Because this perfectly supports our proposed HNF4α-RXRα-KC axis, we have now included these data in the revised manuscript (Results section and Supplementary Figure S5E and S5F).

"Because our earlier data established HNF4α as the upstream transcriptional driver of hepatic Rxra, we queried our published bulk RNA-seq dataset from hepatocyte-specific HNF4α knockout mice ($HNF4\alpha^{iAlbKO}$) (GSE260635). Consistent with the HNF4α-RXRα regulatory axis, HNF4α-deficient livers exhibited a similar and significant downregulation of both the essential hepatocyte-derived niche factors (Supplementary Figure S5E) and the KC-core identity signature (Supplementary Figure S5F), indicating that disruption of this upstream regulator also compromises the Kupffer cell niche."

PPARα, LXR, and FXR: We performed the exact same gene set enrichment analysis on public datasets for PPARα KO (GSE132386), LXRα/β double KO (GSE191030), and FXR KO(GSE58827) mice studies. We are providing the resulting plots here for the reviewer's reference.

- PPARα KO: showed no significant downregulation in either the niche signals (EV2) or the KC-core genes (EV3) (one-sample t-test), indicating the KC niche remains intact. This lack of an overt baseline phenotype could potentially be explained by functional redundancy or compensatory metabolic mechanisms by other nuclear receptors following the loss of PPARα.
- LXR KO: did not show a significant change in the niche signals (EV2), though it did show a significant upregulation in KC-core genes (EV3) (one-sample t-test). This suggests a potential enrichment or baseline activation of the KC pool (rather than depletion), though we note this dataset was limited by an n=2/group.

- FXR KO: In contrast to the LXR model, the FXR KO yielded results similar to LXR, with no significant drop in niche signals (EV2), but a significant decrease in KC-core genes (EV3) (one-sample t-test), suggesting some level of KC depletion or loss of KC identity. (Note that we had to rely on published FPKM values as raw counts were unavailable, limiting the depth of our re-analysis).

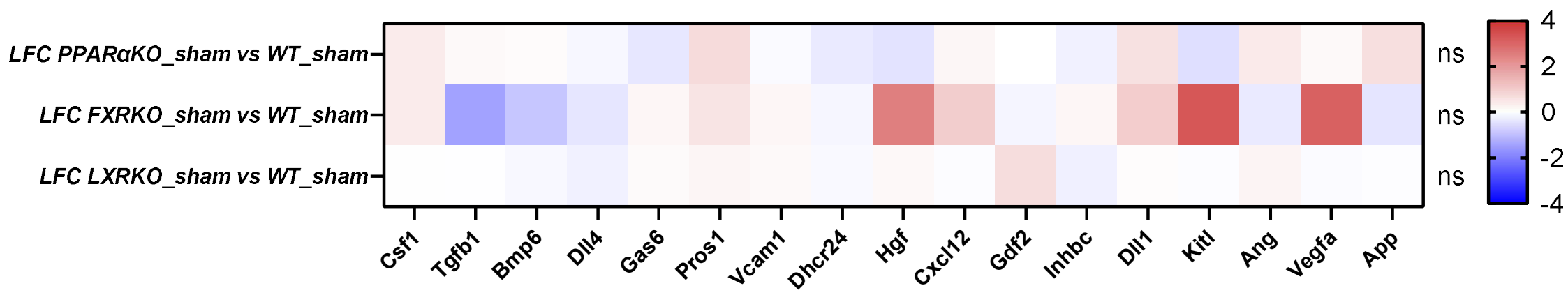

EV2: Heatmap displaying the log2 fold-changes (LFC) of essential niche factors and signaling molecules required for Kupffer cell homeostasis. The bulk RNA-seq data compares basal (sham) livers from PPARαKO, FXRKO, and LXRKO mice to wild-type (WT) controls. Significance was calculated with one-sample t-test.

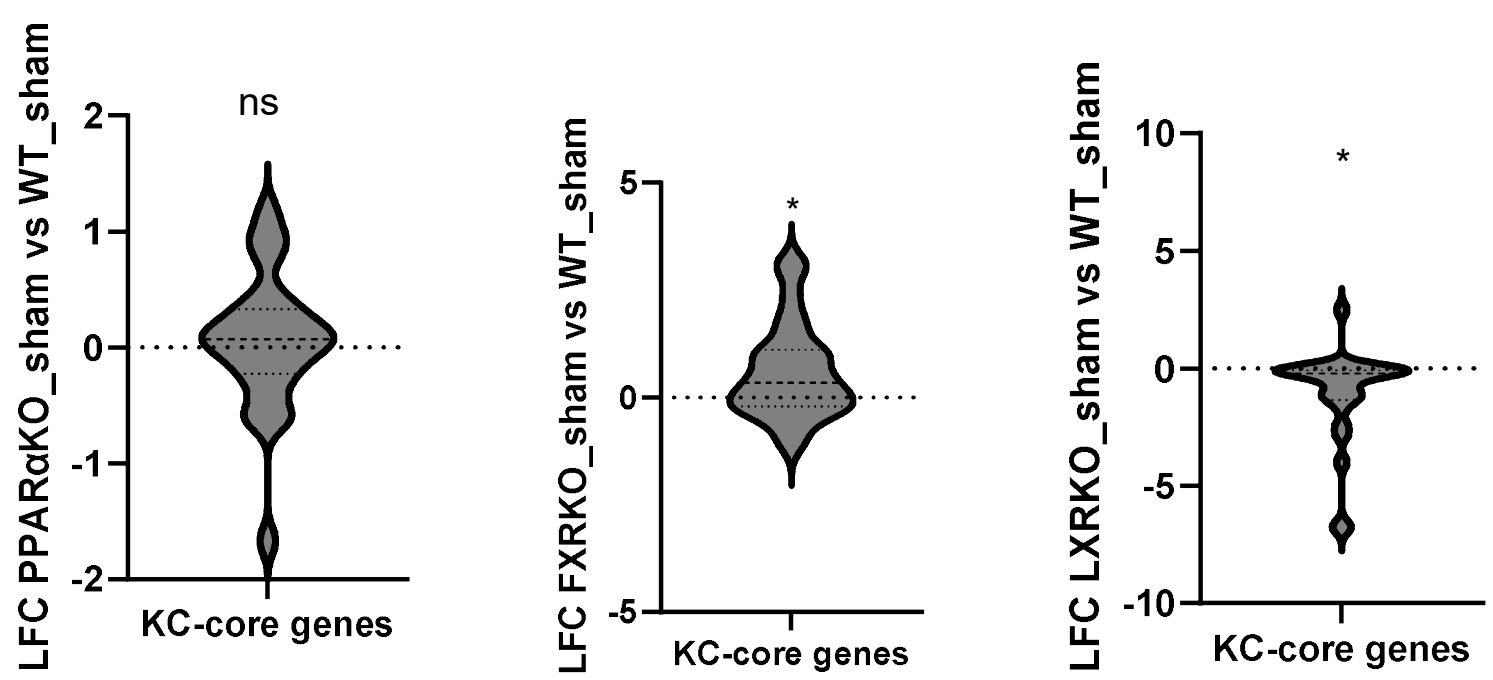

EV3: Violin plots illustrating the distribution of log2 fold-changes (LFC) for the Kupffer cell (KC)-core identity gene signature in the livers of PPARαKO, FXRKO, and LXRKO mice compared to WT controls under basal (sham) conditions. Statistical significance indicates the global shift of the KC-core signature within each knockout model (one-sample t-test); * $p < 0.05$, ns = not significant.

These comparative analyses reveal an interesting distinction. While knocking out LXR or FXR may lead to some level of Kupffer cell loss, neither model replicates the coordinate collapse of the hepatocyte-derived niche signals seen in the RXRα and HNF4α knockouts. This strongly reinforces our conclusion that the HNF4α-RXRα axis uniquely operates as the central regulatory hub responsible for maintaining the molecular microenvironment required for Kupffer cell survival. As the HNF4α data directly builds on the mechanistic narrative of our paper, we have added it to the manuscript, while keeping the broader public dataset re-analyses here for the reviewers.

3c) The decreased expression of Kupffer cell core genes in Fig S5B and D was detected using RNA preps from whole liver. Therefore, these data simply reflect the reduced number of Kupffer cells in RXRa-KO livers and in wild type livers following CLP. It would be interesting to compare Kupffer cell transcriptomes after FACS sorting in wild type livers and in cells remaining in CLP livers to evaluate effects independent of hepatic niche perturbation.

We completely agree with the reviewer's assessment that the decreased expression of KC-core genes in the bulk RNA-seq data primarily reflects the physical reduction of Kupffer cell numbers within the tissue that we observed in Figure 5H. While we originally intended this as an indirect corroboration of our flow cytometry data, we have now explicitly clarified in the Results text that this bulk downregulation directly reflects the physical reduction of the KC pool.

> *"Consistent with a disrupted hepatic microenvironment and lower KC numbers, bulk liver RNA-seq confirmed a significant downregulation ($p < 0.0001$) of the KC-core identity signature (e.g., Clec4f, Timd4, Vsig4) in sham RXRα^{iAlbKO} compared to control liver (Supplementary Figure S5C), which directly reflects the physical reduction of the Kupffer cell pool within the bulk tissue."*

Next, we agree that evaluating the transcriptomic shifts of the surviving Kupffer cells independent of this physical depletion provides crucial insight into their functional state during sepsis. To address this, we analysed RNA-seq data from FACS-sorted KCs isolated from WT mice subjected to either Sham or CLP surgery at the 8 h timepoint. This analysis utilizes a newly available dataset (GEO GSE311736). The results underscore a severe functional compromise in the surviving macrophage population.

As now detailed in the Results section and shown in Supplementary Figures S5H-J, the surviving KCs undergo significant transcriptomic reprogramming. Pathway analysis reveals a prominent induction of an inflammatory signature (Supp. Fig. 5I). Intriguingly, the downregulated transcriptome is significantly enriched for the suppression of Rho GTPase signaling pathways (Supp. Fig. 5J). Because Rho GTPases are essential drivers of actin cytoskeletal remodeling and phagocytosis in macrophages, this signature might indicate that the acute septic insult not only drives the surviving KCs toward a hyper-inflammatory state, but also actively paralyzes the structural machinery they require for efficient bacterial engulfment.

This new analysis demonstrates that CLP not only physically depletes the KC niche, but it also fundamentally impairs the functional competence of the cells that manage to survive. We thank the reviewer for suggesting this analysis, as it significantly strengthens our characterization of the septic liver microenvironment.

> *"We evaluated the transcriptomic reprogramming of Kupffer cells independent of their physical depletion within the bulk liver, we performed RNA-seq on FACS-sorted KCs isolated from WT mice following sham or CLP surgery. Differential expression and pathway enrichment analysis revealed that surviving KCs in septic livers undergo profound phenotypic shifts (2464 DEGs) (Supplementary Figure S5H). The upregulated transcriptome in the surviving KCs was prominently enriched for inflammatory signatures (Supplementary Figure S5I). Conversely, the downregulated transcriptome showed a significant suppression of pathways associated with Rho GTPase signaling (Supplementary Figure S5J). Given that Rho GTPases are fundamental regulators of actin cytoskeleton dynamics and phagocytic cup formation in macrophages, this transcriptomic signature suggests that the KCs surviving the acute septic insult not only adopt a hyper-inflammatory phenotype, but also become fundamentally compromised in their structural and phagocytic competence."*

> *"The RNAseq analysis of FACS-sorted Kupffer cells from WT mice 8 hours post-sham or CLP surgery was performed using a publicly available dataset (GEO GSE311736)."*

3d) Measurements of the total number of Kupffer cells and the number of phagocytosing cells in CLP condition and in conditions after treatment with Bex should be included.

We agree that quantifying Kupffer cell numbers and their phagocytic capacity under Bex treatment provides the necessary functional link to explain how RXR agonism improves bacterial clearance and

survival. To address this, we have performed the requested *in vivo* phagocytosis assay (using pHrodo-labeled *E. coli*) in WT mice subjected to CLP, comparing prophylactic Bex treatment to vehicle (DMSO) controls (see Figure 5 K-O, and Supplementary Figure S5K). We are pleased to report that these new experiments perfectly validate our functional data, revealing a dual mechanism of protection (i.e; higher total number of KC and higher % of phagocytosing KCs);

> *"Furthermore, to determine whether pharmacological activation of RXRα can counter this infection-induced macrophage depletion and improve functional bacterial clearance, we evaluated KC populations and their phagocytic capacity in WT mice subjected to CLP and treated prophylactically with either vehicle (DMSO) or Bex. Consistent with our survival and bacterial burden data (Figure 3), when assessing in vivo phagocytosis using pHrodo-labeled E. coli, we found that Bex treatment significantly increased both the percentage of pHrodo^{+} KCs actively engulfing bacteria (**Figure 5K**) and their per-cell phagocytic capacity (measured as the Mean Fluorescence Intensity (MFI), which reflects a higher quantity of internalized bacteria within acidic phagosomes per cell) (**Figure 5L**). Consequently, the absolute number of phagocytosing KCs per liver was approximately twofold higher in the Bex-treated cohort (**Figure 5M**). Bex treatment significantly preserved the total number of KCs per liver (**Figure 5N**), although their overall proportion within the hepatic CD45^{+} leukocyte pool remained comparable to DMSO-treated septic controls (**Figure 5O**). This demonstrates that RXR agonism enhances systemic bacterial clearance through a dual mechanism. It not only preserves the total pool of resident KCs available to filter pathogens, but it also boosts their intrinsic phagocytic activity during the acute phase of sepsis."*

4) The data in Fig5 E are important, because they show that the phagocytosis potential of the Kupffer cells is actually increased, which reflects possible compensatory effect and that the main mechanism of immune dysfunction in RXR-KO mice is due to the reduced number of Kupffer cells. Representative spectral flow cytometry images should be shown next to the bar graphs.

We completely agree with your interpretation: the fact that the individual surviving Kupffer cells in the RXRα knockout show an increased phagocytic potential (MFI) strongly suggests a compensatory mechanism attempting to make up for the dangerously reduced absolute number of cells. We have now explicitly incorporated this "compensatory effect" interpretation into the Results section of the revised manuscript.

> *"To determine whether this phenotype might involve altered phagocytic function of liver-resident macrophages, we assessed Kupffer cell (KC) phagocytosis in naïve mice using i.v. injection of pHrodo-labeled E. coli in vivo (**Figure 5D**). The proportion of pHrodo^{+} Kupffer cells was comparable between RXRα$^{fl/fl}$ and RXRα^{iAlbKO} mice (**Figure 5E**), suggesting that the probability of bacterial uptake by individual KCs is not altered by hepatocyte RXRα loss. However, KCs in RXRα^{iAlbKO} mice exhibited significantly higher mean fluorescence intensity (MFI) (**Figure 5F**, Supplementary Figure S5B), implying enhanced per-cell phagosomal bacterial uptake in the remaining cells, which likely reflects a functional compensatory mechanism by the surviving macrophage pool. However, the absolute number of phagocytosing KCs per liver was 2-fold reduced in the RXRα^{iAlbKO} mice (**Figure 5G**, Supplementary Figure S5B). Consistently, both the total KC population (**Figure 5H**) and their fraction within CD45^{+} hepatic leukocytes (**Figure 5I**) were significantly diminished in naïve RXRα^{iAlbKO} mice, indicating a shift in liver immune composition secondary to hepatocyte RXRα loss."*

Furthermore, we agree that visualizing the raw flow cytometry data is important for readers to fully appreciate these shifts. To avoid overcrowding the main Figure 5 (which now also includes the

extensive new Bex flow cytometry panels requested in a previous comment), we have added the representative spectral flow images to the supplementary materials. Specifically, the representative flow plots demonstrating the pHrodo fluorescence shifts for the RXRα-KO model can now be found in Supplementary Figure S5B, and the corresponding representative plots for the new Bex-treated CLP experiments can be found in Supplementary Figure S5K. We have referenced these supplementary figures directly next to their corresponding bar graphs in the main text.

Referee #2 (Comments on Novelty/Model System for Author):

The manuscript contains some interesting and novel findings but is insufficiently discussing limitations and also pre-existing knowledge in the field including own data by the group. In the reviewer's opinion, the manuscript will only be suitable for publication after a thorough, (self)critical review and discussion.

Referee #2 (Remarks for Author):

The study by Jelinek et al. is using pharmacological and genetic approaches to explore immune functions of the hepatocyte retinoid receptor α (RXRα) in a mouse model of polymicrobial sepsis. The data of the authors reveal suppression of RXRα signaling during sepsis progression and a protective role of the RXRα agoinist bexarotene on sepsis induced mortality. Hepatocyte specific RXRα deficiency was shown to provoke bacteremia involving a loss of undefined vitalizing effects of hepatocytes on liver resident myeloid Kupffer cells. These data are new and significantly deepen the insights in the complex regulatory functions and mechanisms of RXRa in the mouse liver.

We thank the reviewer for the positive and constructive feedback. We have carefully addressed all comments raised by the reviewer, and the corresponding changes incorporated into the revised manuscript are highlighted in yellow

Nevertheless, some aspects need attention:

Major comments

The integration of the authors' experimental data into the current state of knowledge regarding the role of RXR receptors in sepsis-relevant processes is consistently inadequate. As a prime example the study by Nunez et al. (PNAS 2010) might be mentioned, which, contrary to the authors results, shows reduced mortality in a myeloid RXRalpha knockout model. Whereas the decreased chemokine in myeloid ko mice has been mentioned in the discussion part of the present manuscript the striking result of lower susceptibility of these mice to sepsis has been ignored.

Similar shortcomings exist in the presentation of the medical relevance of the results. Mouse models are useful, but due to the animals' lifestyle and lifespan, they can only provide limited information regarding clinically applicable therapeutic approaches. This needs to be addressed via translational material/data or at least clearly be stated.

We thank the reviewer for highlighting the Núñez et al. (2010) study and for pointing out the necessity of addressing the translational limitations of our animal models. We agree that integrating these points significantly strengthens the manuscript by providing a more balanced view of RXR biology and its clinical applicability.

We agree with the reviewer that the striking survival benefit observed in the myeloid RXRα knockout model reported by Núñez et al. is a crucial piece of the literature. We have now more explicitly incorporated this study into our Discussion. We contrast their findings with our own to highlight the highly compartmentalized, cell-type-specific roles of RXRα in sepsis. Specifically, we emphasize that while myeloid RXRα drives chemokine production (CCL6/CCL9) and potentially lethal hyper-

inflammation (making its deletion protective), hepatocyte RXRα is required to maintain metabolic competence and Kupffer cell survival (making its deletion detrimental).

We also appreciate the reviewer's point regarding the medical relevance of the results. To bridge the translational gap inherent to murine models, we deliberately incorporated transcriptomic data from dogs with naturally acquired septic peritonitis in our study. This outbred model with naturally occurring infection provides a highly clinically relevant intermediate to validate our core findings across species. Furthermore, we now explicitly refer in the manuscript to the findings of Dubois et al. (2020), who demonstrated that the expression of master liver-identity transcription factors—including key RXRα heterodimeric partners—is severely repressed in the livers of deceased human sepsis patients.

Finally, we would like to inform the reviewer that our group is currently analyzing genome-wide data from liver biopsies obtained from living human sepsis patients and matched controls. To our knowledge, this has not been done before, as previous studies primarily relied on post-mortem samples. Our preliminary analysis of these human patients confirms that hepatic RXRα loss-of-function indeed occurs during human sepsis (manuscript in preparation), further substantiating the medical relevance of the pathways described in this murine study.

We have added a new paragraph to the Discussion explicitly acknowledging the limitations of murine models, highlighting the dog and human clinical data, and noting that the prophylactic efficacy of our pharmacological intervention (Bex) faces translational hurdles, as human patients present with established disease. These changes have been incorporated into the latter half of the Discussion section in the revised manuscript.

> *"Importantly, the role of RXRα in sepsis appears to be highly cell-type specific, a distinction that becomes clear when comparing our findings with existing literature. While our data demonstrate that hepatocyte RXRα is protective and essential for maintaining KC populations and systemic antibacterial defense, Núñez et al. (2010) demonstrated that targeted deletion of RXRα in the myeloid compartment actually prolongs mouse survival in both CLP- and LPS-induced sepsis models. In their study, myeloid RXRα deficiency resulted in decreased macrophage production of the chemokines CCL6 and CCL9 (Núñez et al., 2010). This reduction impaired leukocyte recruitment to inflammatory sites, effectively protecting the mice from the detrimental hyper-inflammatory phase of sepsis (Núñez et al., 2010). This striking contrast highlights a highly compartmentalized function for RXR signaling during systemic infection: whereas myeloid RXRα acts as a driver of potentially lethal hyper-inflammatory leukocyte recruitment, hepatocyte RXRα functions as a critical immunometabolic hub required to prevent metabolic collapse and indirectly sustain local immune niches.*
>
> *Although our multi-omics and genetic approaches converge on the HNF4α-RXRα-KC axis, a limitation of our study is the absence of a definitive 'rescue' experiment (such as specifically preserving hepatocyte RXRα expression during infection) to establish absolute causal proof. Furthermore, translating these findings to human sepsis presents inherent challenges. Murine models cannot fully capture the immunological heterogeneity and complex comorbidities of human patients. Crucially, our mechanistic data derive primarily from an untreated CLP model, which lacks the human standard-of-care clinical interventions, like fluid resuscitation and broad-spectrum antibiotics, that define modern sepsis management.*
>
> *However, our inclusion of a naturally acquired canine sepsis model demonstrates that RXRα loss-of-function is a conserved feature across species and also in a clinically relevant setting. This is further supported by human data from Dubois et al. (2020), showing that master liver-identity transcription factors (including key RXRα partners) are profoundly repressed in the livers of deceased sepsis patients (Dubois et al., 2020). Additionally, the prophylactic and early post-CLP*

efficacy of Bex observed here may be difficult to replicate clinically, as patients typically present with severe, established systemic inflammation. Overcoming this therapeutic hurdle likely requires finding and reverting the upstream cause of HNF4α loss-of-function. Stabilizing HNF4α could restore the RXRα signaling axis, potentially enabling therapeutic Bex administration, perhaps in combination with other ligands like PPARα that are also 'victims' of the HNF4α collapse. Future translational studies using humanized models or clinical datasets are therefore essential to evaluate the true therapeutic viability of targeting the HNF4α-RXRα axis."

Referee #3 (Remarks for Author):

This is a very elegant study, using different experimental approaches to investigate the role of RXRalpha suppression in sepsis. The results and methodological setup requires however some clarification, as the design or rationale is not always clear.

We thank the reviewer for the positive and constructive feedback. We have carefully addressed all comments raised by the reviewer, and the corresponding changes incorporated into the revised manuscript are highlighted in yellow.

Specific comments:

1. The introduction could use some citations other than the own work of the authors. E.g. PMID: 30677458 important role for PPARalpha, PMID: 32407006 on HNF4alpha and NR

We thank the reviewer for this constructive feedback and for bringing these two highly relevant studies to our attention. We completely agree that broadening the citations in the introduction provides a more comprehensive background on the role of nuclear receptors in sepsis.

We have updated the Introduction to include these references. We incorporated the findings of Dubois et al. (PMID: 32407006) to highlight how endoplasmic reticulum stress acts as a critical upstream driver of the rapid suppression of HNF4α and other master nuclear receptors, resulting in the loss of hepatic molecular identity during sepsis. We integrated the work of Paumelle et al. (PMID: 30677458) to emphasize the indispensable role of hepatic PPARα in coordinating the vital metabolic shift from glucose to lipid utilization to prevent mortality in sepsis.

These additions frame the severe metabolic consequences of nuclear receptor failure in the septic liver.

> *"Hepatic nuclear factor 4α (HNF4α, NR2A1) is a key NR in the liver that integrates the aforementioned metabolic and inflammatory signals, emerging as a central regulator of metabolism and survival in sepsis. Progressive loss of HNF4α in the septic liver was observed in mouse sepsis models and disrupts the expression of several other NRs, including PPARα, impairing fatty acid metabolism (Van Wyngene et al., 2020; Van Dender et al., 2024). This suppression of hepatic molecular identity, which involves the rapid protein loss of HNF4α and other master nuclear receptors, is actively driven by endoplasmic reticulum stress during acute liver injury and sepsis (Dubois et al., 2020). Furthermore, hepatic PPARα has been shown to be critically required for the host's protective metabolic adaptation to sepsis, as its deficiency prevents the essential shift from glucose to lipid utilization resulting in increased mortality (Paumelle et al., 2019)."*

2. It should be better clarified in the text and figure legends that the first part of the results (Figure 1A-G) are collected from previous studies from the same authors. Also, n=3 is quite a low number for transcriptomics analysis?

We thank the reviewer for pointing this out. We have now explicitly clarified in both the results section and the figure legend for Figure 1 that the data presented in panels 1A-G are derived from the re-analysis of our previously published datasets.

> *"To characterize hepatic RXRα dynamics during early sepsis, we first analyzed our previously published bulk RNA-sequencing (RNA-seq) datasets (GEO GSE139484) on livers derived from wild-type (WT) mice eight hours after CLP or sham surgery (**Figure 1A**); this timepoint represents an optimal window where septic symptoms are clinically manifest but preceding late-stage decline (Van Wyngene et al., 2020)."*

> *"Next, using tamoxifen-inducible hepatocyte-specific HNF4α knockout mice (HNF4α^{iAlbKO}), we investigated whether sepsis-induced suppression of RXRα is driven by HNF4α. We analyzed our recently published RNA-seq data (GEO GSE260635) from HNF4α^{iAlbKO} and control (HNF4α$^{fl/fl}$) livers collected eight hours after sham or CLP surgery (**Figure 1E**)."*

> *"Note: Data presented in panels A–G were generated from the re-analysis of our previously published cohorts; GEO GSE139484 and GSE260635."*

Regarding the sample size for the transcriptomics analysis (n=3/group), we acknowledge the reviewer's concern. While n=3 is a minimal sample size, it provides sufficient statistical power in this specific context for several reasons. First, we use highly controlled, inbred mouse strains housed under strictly standardized environmental conditions, which intrinsically minimizes biological variation and intra-group variance. Second, the biological effect size of the CLP-induced septic response at 8 hours is extremely large and robust, allowing for the reliable detection of genome-wide transcriptional shifts even with a smaller sample size. Furthermore, the transcriptomic trends we observed are strongly corroborated by orthogonal methods within the study. For instance, the RNA-level downregulation of RXRα is highly consistent with our LFQ proteomics data, which utilizes a much larger sample size (n=7-8/group, Figure 1C).

Finally, to directly address the limitations of a small inbred murine cohort, we extended our transcriptomic analysis to a naturally acquired canine sepsis model (Figure 1H-K, Supplementary Figure S1B-C). This outbred dog cohort introduced significant biological variation and utilized a substantially higher sample size (n=9 controls, n=14 septic). Even within this highly variable, clinically relevant setting, we observed the exact same fundamental finding: a profound loss-of-function signature for RXRα and its heterodimeric partners. Because the murine RNA-seq data serves primarily as a foundational observation that is subsequently validated by orthogonal downstream assays and cross-species cohorts, we are highly confident in the reproducibility and biological validity of these transcriptomic results.

3. In the first analysis (Fig 1D) it is stated 'prioritizing metabolic dysfunction over the more extensively characterized inflammatory response" what do you mean by that? How was the analysis done? Did the authors selectively excluded specific pathways? Was the same methodology used in the following signaling pathways analyses 5Fig 1G, Fig 1J, Fig2D, Fig 3D?

We thank the reviewer for pointing out this ambiguous phrasing, and we apologize for the confusion it caused. We want to clarify that we absolutely did not selectively exclude, filter, or manually curate any specific pathways from our results.

The original phrasing was simply intended to explain our rationale for focusing our enrichment analysis specifically on the downregulated gene set rather than the upregulated genes. In the context of sepsis, upregulated genes overwhelmingly map to the well-characterized systemic inflammatory and immune responses. By strictly analyzing the complete set of significantly downregulated genes (adj $p < 0.05$)

using the Enrichr platform, the unbiased results naturally and predominantly pointed toward metabolic dysfunction.

We put the entire list of significantly downregulated genes into Enrichr and simply reported the top five most significantly enriched pathways and transcriptional regulators. No pathways were manually excluded or selectively prioritized.

This exact same unbiased methodology (analyzing the complete downregulated gene sets via Enrichr and reporting the top results without filtering) was applied consistently for the pathway analyses shown in Figures 1G, 1J, 2D, and 3D.

We have revised the text in the Results section to remove the confusing phrasing and to explicitly state that the analysis was unbiased and performed using Enrichr.

> *"We performed enrichment analysis (using the Enrichr platform) of genes downregulated eight hours after CLP in WT mice (n=3/group; adj $p < 0.05$) to study the signaling pathways negatively affected in the liver after sepsis. Because the upregulated gene signature predominantly reflects the already well-characterized immune and inflammatory responses, we focused our unbiased analysis specifically on the downregulated gene set. In fact, increasing evidence suggests that transcriptionally repressed (or loss-of-function) programs are key drivers of disease severity during sepsis, particularly through disruption of metabolic and homeostatic functions. In agreement with the reduction in RXRα expression, the top five most significantly perturbed regulators included RXR, PPARα, and LXR* ***(Figure 1D)****."*

4. In the first BEX treatment experiment, what was the rationale to give the injection 6h post CLP/sham?

The 6-hour post-CLP time point was selected because it represents a well-established and standardized early phase of sepsis in our model, during which the acute systemic response is actively developing while overt late-stage organ failure and secondary complications remain limited. This time point therefore enables the investigation of early molecular and transcriptional events associated with sepsis pathogenesis.

Administering the Bex treatment at this specific window allowed us to assess the transcriptional responsiveness of RXRα exactly when the septic milieu is established and the receptor levels are acutely declining.

To clarify this rationale for the readers, we have added a brief explanatory note to the Results section.

> *"To confirm that sepsis-induced downregulation of RXRα mRNA and predicted pathways indeed compromises RXRα-dependent signaling pathways, we treated WT mice with Bex (a validated pan-RXR agonist) or vehicle (DMSO) six hours after sham or CLP surgery, and harvested livers two hours later for bulk RNA sequencing (****Figure 2A****). At this 6-hour post-CLP time point, the acute systemic response is actively developing, while late-stage organ failure and secondary complications remain limited."*

5. Why is the number of animals in the survival analysis in Fig3I different between groups? With only n=5 in the 2 KO groups, it is not surprising that no statistical difference was reached. Was a power calculation performed beforehand? In the follow-up experiment (Fig4 B) 15/16 animals per group were tested; What is the rationale to these different numbers?

We agree that the low number of animals in the initial KO groups (n=5) might have left the study underpowered to reach definitive statistical significance in that specific comparison, and the imbalance between the WT and KO groups was a valid concern.

No formal *a priori* power calculation was performed for the initial survival experiment. The initial experiment (with n=8 for the WT groups and n=5 for the KO groups) revealed a clear trend towards a difference in survival between KO and WT mice, but this did not reach statistical significance, likely due to limited sample size. The discrepancy in animal numbers in our initial submission was strictly due to breeding constraints. Generating sufficient numbers of the tamoxifen-inducible RXRα^{iAlbKO} transgenic mice (with age- and sex-matched littermate controls) takes considerable time.

Therefore, the experiment was independently repeated under the same experimental conditions to assess reproducibility and increase statistical power. Importantly, animals were added through an independent replicate experiment rather than through interim expansion of a single ongoing experiment. To directly address your concern regarding the imbalance between the groups, we have chosen to present here for the reviewer the latest independent survival curve (EV4). Importantly, the exact same trend, where Bex confers a survival benefit in WT mice but fails to protect the KO mice, was consistently observed in every single independent repeat.

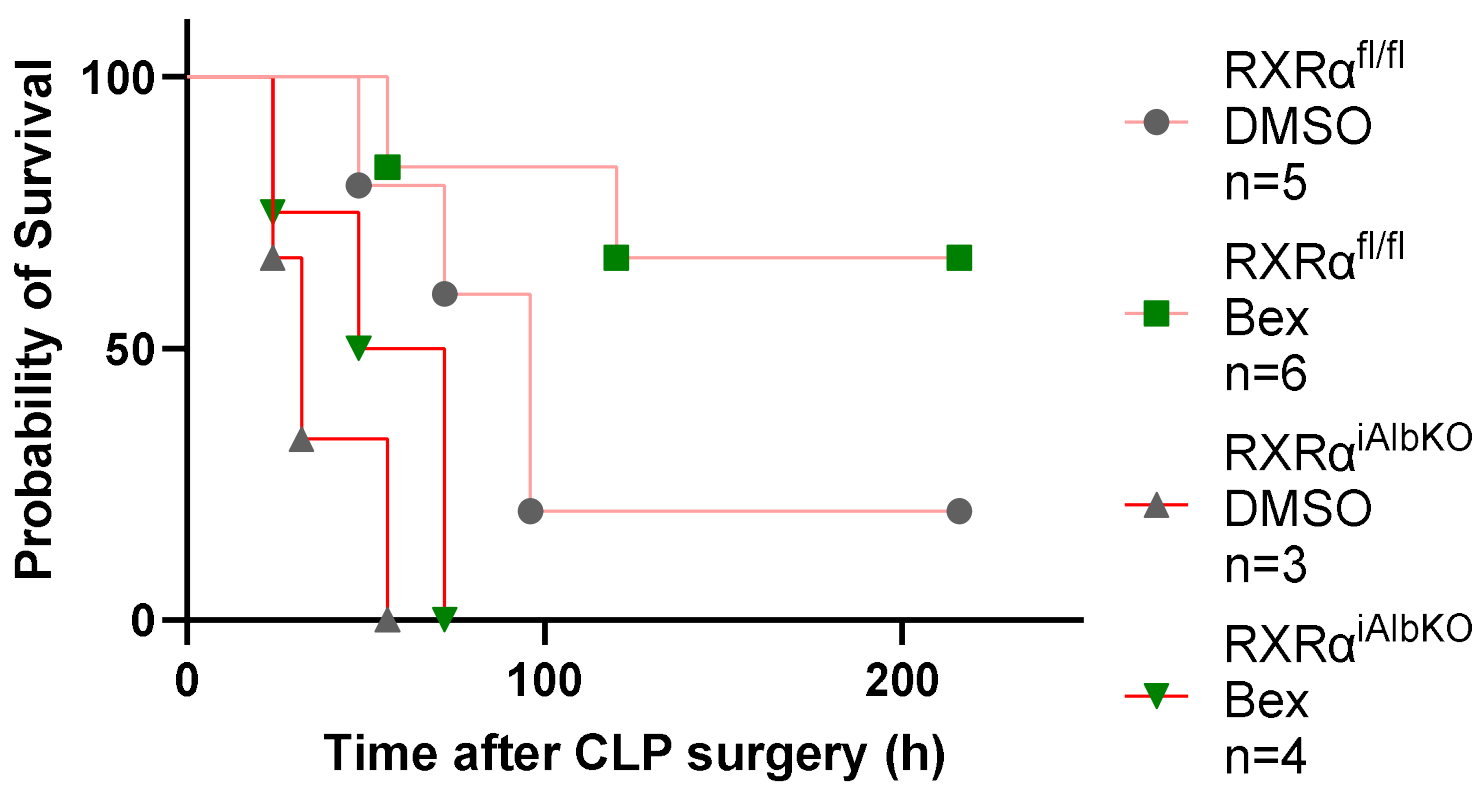

EV4: Kaplan-Meier survival curves representing the second, independent experimental cohort of control (RXRα$^{fl/fl}$) and inducible hepatocyte-specific RXRα knockout (RXRα^{iAlbKO}) mice. Mice were subjected to CLP surgery and treated prophylactically with vehicle (DMSO) or Bex.

We decided to show the complete pooled data from both independent experiments in the revised Figure 3I. The combined analysis of both independent experiments resulted in a statistically significant difference in survival. Thus, the sizes of the groups are 13 and 14 for the WT groups, and 8 and 9 for the KO groups.

The experiment in Figure 4B was designed from the outset to be a definitive, large-scale survival study. Because we anticipated a smaller effect size in the low-lethality model, we deliberately pooled multiple breeding rounds over several months specifically to achieve n=15/16 per group, ensuring we had the robust statistical power needed for that specific comparison.

6. Information on the CLP procedures is to limited. In the methods it is stated that only animals in the survival studies received antibiotics. Did none of the animals on which tissues analyses were performed receive fluids or antibiotics? How many animals died in the non-survival studies with end-points at 8h, or 24h? I am asking because with such a high lethality model, and no treatment ,

you might have created some bias by only selecting animals that survived the CLP until the time of sampling?

We agree that transparency regarding the CLP post-operative care and potential survivorship bias is essential, and therefore we have expanded information on the CLP procedure in the M&M section.

Antibiotics were administered only in the survival experiments, whereas mice included in the tissue collection studies did not receive antibiotic treatment prior to sample isolation. This experimental design was chosen deliberately to allow direct comparison between the 8 h and 24 h timepoints and to assess the natural progression of tissue and inflammatory parameters during sepsis development without therapeutic intervention. Importantly, the first tissue collection timepoint (8 h post-CLP) coincided with the time at which antibiotics would normally have been administered in the survival experiments, so it would not make sense to isolate the mice immediately after injection of antibiotics. For consistency, animals collected at the 24 h timepoint were also not treated with antibiotics, enabling direct comparison of disease progression between both timepoints. Furthermore, no fluid resuscitation was administered in any of the cohorts.

We carefully tracked the mortality in our tissue collection cohorts. No animals died before the predefined 8 h endpoint. In all WT and Bex-treated experiments, zero mice died prior to the scheduled 24 h sampling endpoints. Therefore, there is no survivorship bias in the transcriptomic or tissue analyses of these groups.

At the 24 h endpoint, 4 mice in the RXRα^{iAlbKO} group died prior to sample collection. These animals were excluded from the downstream tissue analyses. We acknowledge that this could potentially introduce a degree of survival bias inherent to severe CLP models. However, the use of identical experimental conditions across genotypes allows relative comparison between groups. We believe this early, accelerated mortality actually reinforces the core hypothesis of our manuscript: the genetic loss of hepatic RXRα renders the host extremely vulnerable to metabolic and immunological collapse during sepsis.

We have updated the Materials and Methods section to explicitly state the absence of fluids and antibiotics in the mechanistic studies, as well as the exact numbers of early dropouts in the knockout cohort.

> *"Animals utilized for short-term mechanistic analyses, tissue sampling, and bacterial counts (8 h and 24 h endpoints) did not receive antibiotics or fluid resuscitation. In these tissue collection studies, no mice died prior to the planned sampling timepoints in the WT or Bex-treated cohorts. However, at the 24 h timepoint, 4 mice from the highly susceptible RXRα^{iAlbKO} group died prior to sample collection; these animals were not included in the downstream tissue analyses."*

7. Please explain how the "low-lethality" was reached in the exp of Fig4A. What is the age and sex of the animals per experiment?

We achieved a "low-lethality" model by modifying the standard CLP protocol. Specifically, we ligated a significantly smaller portion of the cecum (~50% compared to the standard 75% used in our high-lethality models). Because the severity of the CLP model is highly dependent on the length of the ligated tissue, which dictates the volume of necrotic tissue and the extent of polymicrobial leakage into the peritoneum, this reduction produces a milder, sub-lethal septic response. This allowed us to properly assess baseline sensitizations in our knockout mice without immediately overwhelming them. We have now clarified this in both the Results and the Materials & Methods sections.

> *Results section: "Building on the observation that hepatocyte RXRα loss potentially sensitizes mice to septic death (Figure 3I), we sought to further dissect the molecular basis of RXRα-dependent hepatoprotection in sepsis. To do so, we performed a low-lethality CLP (achieved by*

*ligating a smaller portion of the cecum to reduce the severity of the infection) in hepatocyte-specific inducible RXRα knockout (RXRα^{iAlbKO}) and littermate control (RXRα$^{fl/fl}$) mice (**Figure 4A**)."*

M&M section: "For the low-lethality CLP model, the procedure was modified by reducing the ligated portion of the cecum (to approximately 50%) to limit the necrotic burden and subsequent bacterial leakage."

For all experiments utilizing WT mice (including the basal CLP, sham, and Bex treatment studies), we used exclusively 8-week-old male mice sourced from Janvier Labs to minimize biological variance. For the genetic models (the RXRα^{iAlbKO} mice and their fl/fl littermate controls), we utilized both male and female mice aged between 8 and 20 weeks. We have explicitly added this demographic breakdown to the "Mice" subsection of the Materials & Methods.

"For experiments utilizing wild-type (WT) mice (including CLP, sham, and Bex- and DMSO-treated cohorts), exclusively 8-week-old male mice were used (obtained from Janvier Labs). For experiments involving the transgenic RXRα^{iAlbKO} mice and their littermate controls, both male and female mice aged between 8 and 20 weeks were used from in house breeding colonies."

8. The authors suggest a deprivation of hepatocyte-derived metabolic support to the Kuppfer Cells, but this remains very speculative. Any information on the metabolic signature in the CLP RXR KO mice?

We agree that the proposed deprivation of hepatocyte-derived metabolic support to Kupffer cells remains speculative in the absence of direct metabolic analyses. In the current study, we did not perform metabolomic profiling of liver tissue or isolated Kupffer cells from CLP RXRα^{iAlbKO} mice. We acknowledge that the lack of targeted lipidomics or metabolomics is a limitation of our study, which we now explicitly state in the Discussion section.

Therefore, we cannot directly conclude which metabolic pathways are physically altered in these animals that might contribute to altered KC functions. Our interpretation is based on the observed phenotype together with the established role of hepatocyte RXRα signaling in regulating hepatic metabolic homeostasis and hepatocyte–macrophage crosstalk. However, to address your question directly regarding the data we do possess, we utilized our RNA-seq dataset to evaluate the transcriptomic "metabolic signature" of the CLP RXRα knockout mice. We found a severe and significant collapse of the specific hepatocellular machinery required to synthesize and export the lipid mediators, oxysterols, and retinoids that Kupffer cells typically rely on for LXR-mediated survival.

We have added this data in the heatmap in the supplement (Supplementary Figure S4J) and explicitly referenced it in the Results section. Specifically, in the septic RXRα^{iAlbKO} livers (compared to their sham baseline), we observed massive downregulation of:

- Oxysterol synthesis enzymes (critical for producing endogenous LXR ligands): Cyp27a1 (LFC -1.65) and Cyp46a1 (LFC -2.23).
- Cholesterol/sterol efflux transporters: Abcg5 (LFC -2.40) and Abcg8 (LFC -2.01).
- Retinoid and lipid carriers: Rbp4 (LFC -1.23) and Apoe (LFC -0.51).

"Furthermore, the septic RXRα^{iAlbKO} livers exhibited a profound suppression of the enzymatic machinery and transporters required for oxysterol synthesis and lipid/retinoid efflux (e.g., Cyp27a1, Cyp46a1, Abcg5, Abcg8, Rbp4), pointing toward a severe transcriptional failure in the production and export of essential lipid mediators (Supplementary Figure S4J)."

While our biochemical data also show significantly elevated plasma free fatty acids and severe hypoglycemia (Supplementary Figure S4E–F), we recognize that this profound systemic collapse is likely a secondary consequence of the uncontrolled bacterial load rather than the primary driver of KC depletion. We have added these specific gene targets to the Discussion to concretely illustrate the transcriptomic signature that forms the basis of our hypothesis. We have revised the manuscript to more clearly state that this represents a hypothesis rather than a demonstrated mechanism, and that future studies will be required to confirm this.

> *"While direct lipidomic profiling was not performed in this study, our parallel-omics data provide a strong transcriptomic proxy for this hypothesized metabolic deprivation. Specifically, during sepsis, RXRα-deficient livers exhibit a profound suppression of the machinery required to synthesize and export LXR/RXR ligands, including major oxysterol synthesis enzymes (Cyp27a1, Cyp46a1), cholesterol transporters (Abcg5, Abcg8), and lipid/retinoid carriers (Rbp4, Apoe). However, we acknowledge that this deprivation of hepatocyte-derived metabolic support remains speculative in the absence of direct metabolic analyses. Future studies incorporating liver and/or Kupffer cell metabolomic profiling will be required to determine definitively whether altered hepatocyte-derived metabolic support functionally contributes to the impaired immune response observed in RXRα^{iAlbKO} mice during sepsis."*

9. Although the story includes a lot of different interventions, there is no 'rescue' experiment that ties the different aspects together. Furthermore, these findings in an untreated CLP mouse model cannot easily be extrapolated to a therapeutic intervention for humans. The translation from mice to men has failed more than it has succeeded. These limitations need to be discussed.

We thank the reviewer for this critical and realistic assessment of our work. We fully agree that addressing the limitations of our experimental approach, particularly the lack of a singular 'rescue' experiment and the gap between murine models and clinical reality, is necessary for a balanced discussion.

We agree that while our study utilizes several independent angles (genetic knockouts, pharmacological agonism, and multi-omics) that strongly converge on the same mechanistic axis, tying them together into a single causal proof *in vivo* is difficult. As the reviewer rightfully suggests, a definitive 'rescue' experiment would involve preserving RXRα function specifically in hepatocytes during sepsis to demonstrate enhanced survival, preserved phagocytosis, and reduced bacterial counts. However, generating a transgenic model capable of sustaining hepatic RXRα expression against the profound, sepsis-induced transcriptional collapse is technically challenging and beyond the scope of this current study. We have now explicitly acknowledged this inherent limitation of complex *in vivo* modeling in the Discussion.

The reviewer makes an excellent point regarding the historical failure rate of translating mouse sepsis therapies to human medicine. We recognize that our use of an untreated CLP model for mechanistic insights inherently limits direct clinical extrapolation, as it lacks standard clinical interventions like fluid resuscitation and broad-spectrum antibiotic therapy. To partially bridge this translational gap, we deliberately incorporated the canine study to demonstrate that RXRα loss-of-function is also present in other species in a highly clinically relevant, naturally acquired setting. Furthermore, we must point to the work of Dubois et al., which shows that in clinical liver samples from deceased human sepsis patients, master liver-identity transcription factors (including crucial RXRα heterodimeric partners) are significantly repressed.

Finally, we would like to inform the reviewer that our group is currently analyzing genome-wide data from liver biopsies obtained from living human sepsis patients and matched controls. This is a critical

step, as comparing deceased sepsis patients to living controls must be interpreted carefully. Our ongoing analysis aims to verify whether our observations of HNF4α and RXRα loss-of-function hold true in real, living sepsis patients, which will be the subject of a future report.

We have revised the limitations paragraph in the Discussion to highlight the lack of standard-of-care interventions in our untreated CLP model as a major hurdle for immediate therapeutic extrapolation, and we explicitly state that the translation from mice to humans remains a significant challenge.

> *"Although our multi-omics and genetic approaches converge on the HNF4α-RXRα-KC axis, a limitation of our study is the absence of a definitive 'rescue' experiment (such as specifically preserving hepatocyte RXRα expression during infection) to establish absolute causal proof. Furthermore, translating these findings to human sepsis presents inherent challenges. Murine models cannot fully capture the immunological heterogeneity and complex comorbidities of human patients. Crucially, our mechanistic data derive primarily from an untreated CLP model, which lacks the human standard-of-care clinical interventions, like fluid resuscitation and broad-spectrum antibiotics, that define modern sepsis management.*
>
> *However, our inclusion of a naturally acquired canine sepsis model demonstrates that RXRα loss-of-function is a conserved feature across species in a clinically relevant setting. This is further supported by human data from Dubois et al. (2020), showing that master liver-identity transcription factors (including key RXRα partners) are profoundly repressed in the livers of deceased sepsis patients (Dubois et al., 2020). Additionally, the prophylactic and early post-CLP efficacy of Bex observed here may be difficult to replicate clinically, as patients typically present with severe, established systemic inflammation. Overcoming this therapeutic hurdle likely requires finding and reverting the upstream cause of HNF4α loss-of-function. Stabilizing HNF4α could restore the RXRα signaling axis, potentially enabling therapeutic Bex administration, perhaps in combination with other ligands like PPARα that are also 'victims' of the HNF4α collapse. Future translational studies using humanized models or clinical datasets are therefore essential to evaluate the true therapeutic viability of targeting the HNF4α-RXRα axis."*

10th Jun 2026

Dear Dr. Vandewalle,

Thank you for submitting your revised manuscript to EMBO Molecular Medicine. Your manuscript will be ready for acceptance pending the following final amendments:

1) Figures:
- Please upload main figures as high-resolution files in TIFF, EPS or PDF format. Main figure legends should be placed after References.
- Supplementary figures should be renamed to Figure EV1 etc. and uploaded as individual high-resolution files in TIFF, EPS or PDF format. Please updated their callouts in the main manuscript text. EV figure legends should be placed in the manuscript file after main figure legends under the heading "Expanded View Figure Legends".
2) Source Data: Please upload source data for the main figures as one zipped file per figure.
3) In the main manuscript file, please do the following:
- Please address all comments suggested by our data editors listed below:
o Figure legends:
1. Please note that the exact p values are not provided in the legends of 1B,C,F; 2B,F; 3B-G,I; 4B; 5B,C,F-I,K-N,Q,R; Supplementary Figure S3-A-D; Supplementary Figure S4-E,F; Supplementary Figure S5-C,F,G.
2. Please indicate the statistical test used for data analysis in the legends of figures 1B,C,D,F,G,I,J,K; 2B,D,F; 3C-G; 4D; 5E-I,K-O; Supplementary Figure S1-C; Supplementary Figure S2-A,B; Supplementary Figure S4-A-D,H,I; Supplementary Figure S5-H,I,J.
- Remove data not shown (p. 7).
- Rename Material and Methods to Methods.
- In Methods, provide the antibody dilutions that were used for each antibody.
- Indicate in legends number and nature of replicates and exact p= values, not a range, along with the statistical test used. To keep the figures "clear" some authors found providing an Appendix table Sx with all exact p-values preferable. You are welcome to do this if you want to.
-
- Please remove Reagents and Tools Table and upload it as a separate .docx file. More information on how to adhere to this format as well as downloadable templates (.docx) for the Reagents and Tools Table can be found in our author guidelines: https://www.embopress.org/page/journal/17574684/authorguide#structuredmethods
- Please add "Disclosure and competing interests statement". We updated our journal's competing interests policy in January 2022 and request authors to consider both actual and perceived competing interests. Please review the policy https://www.embopress.org/competing-interests and update your competing interests if necessary.
- Please correct the reference citation in the reference list. Where there are more than 10 authors on a paper, 10 will be listed, followed by "et al.". Also, please remove DOIs. DOIs should only be used for preprints and datasets that have not been published. Please check "Author Guidelines" for more information.
https://www.embopress.org/page/journal/17574684/authorguide#referencesformat
4) Funding: Make sure that information about all sources of funding are complete in both our submission system and in the manuscript. Currently the Research Council of Ghent University (GOA and Methusalem Program) is missing in our submission system. Please correct.
5) Tables: Please upload Supplementary Table 1 as a Dataset and rename it to Dataset EV1. Add the legend in a separate tab/sheet and update its callouts in the main manuscript text.
6) The Paper Explained: Please provide "The Paper Explained" and add it to the main manuscript text. Please check any of recently published EMBO Mol Med research articles for reference.
7) Synopsis: Every published paper now includes a 'Synopsis' to further enhance discoverability. Synopses are displayed on the journal webpage and are freely accessible to all readers. They include separate synopsis image and synopsis text.
- Synopsis image: Please provide a visual abstract as a high-resolution jpeg file exactly 550 px-wide x 300 pixels high to illustrate your article.
- Synopsis text: Please provide a short standfirst (maximum of 300 characters, including space) as well as 2-5 one sentence bullet points that summarise the paper as a .doc file. Please write the bullet points to summarise the key NEW findings. They should be designed to be complementary to the abstract - i.e. not repeat the same text. We encourage inclusion of key acronyms and quantitative information (maximum of 30 words / bullet point). Please use the passive voice.
- Please check your synopsis text and image before submission with your revised manuscript. Please be aware that in the proof stage minor corrections only are allowed (e.g., typos).
8) As part of the EMBO Publications transparent editorial process (see our Editorial at http://embomolmed.embopress.org/content/2/9/329), EMBO Molecular Medicine will publish online a Review Process File (RPF) to accompany accepted manuscripts. This file will be published in conjunction with your paper and will include the anonymous referee reports, your point-by-point response and all pertinent correspondence relating to the manuscript. Let us know if you want to remove or not any figures from it prior to publication. Please note that the Authors checklist will be published at the end of the RPF.

9) Please provide a point-by-point letter INCLUDING my comments as well as the reviewer's reports and your detailed responses (as Word file).

I look forward to reading a new revised version of your manuscript as soon as possible.

Yours sincerely,

Zeljko Durdevic

Zeljko Durdevic
Senior Editor
EMBO Molecular Medicine

*** Instructions to submit your revised manuscript ***

*** PLEASE NOTE *** As part of the EMBO Publications transparent editorial process initiative (see our Editorial at https://www.embopress.org/doi/pdf/10.1002/emmm.201000094), EMBO Molecular Medicine will publish online a Review Process File to accompany accepted manuscripts.

In the event of acceptance, this file will be published in conjunction with your paper and will include the anonymous referee reports, your point-by-point response and all pertinent correspondence relating to the manuscript. If you do NOT want this file to be published, please inform the editorial office at contact@embomolmed.org.

*****

When preparing your revised manuscript, please refer to our guidelines: https://link.springer.com/journal/44321/submission-guidelines#cms-Revised-submissions. We perform an initial quality control of all revised manuscripts before re-review; failure to include requested items will delay the evaluation of your revision.

We require:

1) A .docx formatted version of the manuscript text (including legends for main figures, EV figures and tables). Please make sure that the changes are highlighted to be clearly visible.

2) Individual production quality figure files as .eps, .tif, .jpg (one file per figure). For guidance, download the 'Figure Guide PDF': https://media.springernature.com/original/springer-cms/rest/v1/content/27825798/data/v1.

3) A .docx formatted letter INCLUDING the reviewers' reports and your detailed point-by-point responses to their comments. As part of the EMBO Press transparent editorial process, the point-by-point response is part of the Review Process File (RPF), which will be published alongside your paper.

4) A complete author checklist, which you can download from our author guidelines. Please insert information in the checklist that is also reflected in the manuscript. The completed author checklist will also be part of the RPF.

5) Please note that all corresponding authors are required to supply an ORCID ID for their name upon submission of a revised manuscript.

6) It is mandatory to include a 'Data Availability' section after the Materials and Methods. Before submitting your revision, primary datasets produced in this study need to be deposited in an appropriate public database, and the accession numbers and database listed under 'Data Availability'. Please remember to provide a reviewer password if the datasets are not yet public.

In case you have no data that requires deposition in a public database, please state so in this section. Note that the Data Availability Section is restricted to new primary data that are part of this study.

7) For data quantification: please specify the name of the statistical test used to generate error bars and P values, the number (n) of independent experiments (specify technical or biological replicates) underlying each data point and the test used to calculate p-values in each figure legend. The figure legends should contain a basic description of n, P and the test applied. Graphs must include a description of the bars and the error bars (s.d., s.e.m.).

8) At EMBO Press we ask authors to provide source data for the main manuscript figures. You will receive a separate email with instructions for providing source data with your revised manuscript, including how to upload and organize the files.

9) Our journal encourages inclusion of *data citations in the reference list* to directly cite datasets that were re-used and obtained from public databases. Data citations in the article text are distinct from normal bibliographical citations and should directly link to the database records from which the data can be accessed. In the main text, data citations are formatted as follows: "Data ref: Smith et al, 2001" or "Data ref: NCBI Sequence Read Archive PRJNA342805, 2017". In the Reference list, data citations must be labeled with "[DATASET]". A data reference must provide the database name, accession number/identifiers and a resolvable link to the landing page from which the data can be accessed at the end of the reference.

10) We replaced Supplementary Information with Expanded View (EV) Figures and Tables that are collapsible/expandable online. A maximum of 5 EV Figures can be typeset. EV Figures should be cited as 'Figure EV1, Figure EV2" etc... in the text and their respective legends should be included in the main text after the legends of regular figures.

- For the figures that you do NOT wish to display as Expanded View figures, they should be bundled together with their legends in a single PDF file called *Appendix*, which should start with a short Table of Content. Appendix figures should be referred to in the main text as: "Appendix Figure S1, Appendix Figure S2" etc.

- Additional Tables/Datasets should be labeled and referred to as Table EV1, Dataset EV1, etc. Legends have to be provided in a separate tab in case of .xls files. Alternatively, the legend can be supplied as a separate text file (README) and zipped together with the Table/Dataset file.

11) The paper explained: EMBO Molecular Medicine articles are accompanied by a summary of the articles to emphasize the major findings in the paper and their medical implications for the non-specialist reader. Please provide a draft summary of your article highlighting

- the medical issue you are addressing,

- the results obtained and

- their clinical impact.

This may be edited to ensure that readers understand the significance and context of the research. Please refer to any of our published articles for an example.

12) Author contributions: You will be asked to provide CRediT (Contributor Role Taxonomy) terms in the submission system. These replace a narrative author contribution section in the manuscript.

13) A Conflict of Interest statement should be provided in the main text.

14) Every published paper includes a 'Synopsis' to further enhance discoverability. Synopses are displayed on the journal webpage and are freely accessible to all readers. They include a short stand first (maximum of 300 characters, including space) as well as 2-5 one-sentences bullet points that summarizes the paper. Please write the bullet points to summarize the key NEW findings. They should be designed to be complementary to the abstract - i.e. not repeat the same text. We encourage inclusion of key acronyms and quantitative information (maximum of 30 words / bullet point). Please use the passive voice. Please attach these in a separate file or send them by email, we will incorporate them accordingly.

Please also suggest a striking image or visual abstract to illustrate your article as a PNG file 550 px wide x 300 px high.

15) Include a Reagents and Tools Table as part of the Methods section, which can be downloaded from our author guidelines.

EMBO Molecular Medicine has a "scooping protection" policy, whereby similar findings that are published by others during review or revision are not a criterion for rejection. Should you decide to submit a revised version, I do ask that you get in touch after three months if you have not completed it, to update us on the status.

*Additional important information regarding Figures

Each figure should be given in a separate file and should have the following resolution:

Graphs 800-1,200 DPI
Photos 400-800 DPI
Colour (only CMYK) 300-400 DPI"

Figures are not edited by the production team. All lettering should be the same size and style; figure panels should be indicated by capital letters (A, B, C etc). Gridlines are not allowed except for log plots. Figures should be numbered in the order of their appearance in the text with Arabic numerals. Each Figure must have a separate legend and a caption is needed for each panel.

*Additional important information regarding figures and illustrations can be found at https://media.springernature.com/original/springer-cms/rest/v1/content/27825798/data/v1

***** Reviewer's comments *****

Referee #1 (Remarks for Author):

The authors performed new analyses that clarify the functional interactions of different RXR partners in response to Bexarotene. They provide further analyses assessing systemic effects by Bex treatment using several markers and provide new evidence for CLP-mediated impairment of surviving KCs. The authors have also addressed the minor concerns regarding data presentations. I am satisfied with the answers and the improvements made in the revised version.

Referee #2 (Comments on Novelty/Model System for Author):

The revised version of the manuscript by Jelinek et al. has improved significantly in terms of both data presentation and discussion. In the reviewer's opinion, the quality standards of EMBO MM are now fully met.

Referee #2 (Remarks for Author):

The reviewers' critical comments have been consistently addressed in the revised version. This applies in particular to the integration of the data into the current literature on the role of RXR receptors in sepsis and the presentation of the medical relevance of the data derived from animal models. I therefore unreservedly recommend publication of the manuscript in EMBO MM.

Referee #3 (Comments on Novelty/Model System for Author):

the relevance for the patient setting remains to be proven

Referee #3 (Remarks for Author):

I thank the authors for their elaborate reply to my comments - which were answered adequately

The authors addressed the remaining editorial issues.

24th Jun 2026

Dear Dr. Vandewalle,

We are pleased to inform you that your manuscript is accepted for publication and is now being sent to our publisher to be included in the next available issue of EMBO Molecular Medicine.

Your manuscript will be processed for publication by EMBO Press. It will be copy edited and you will receive page proofs prior to publication. Please note that you will be contacted by Springer Nature Author Services to complete licensing and payment information.

You may qualify for financial assistance for your publication charges - either via a Springer Nature fully open access agreement or an EMBO initiative. Check your eligibility: https://link.springer.com/journal/44321/how-to-publish-with-us

Should you be planning a Press Release on your article, please notify the production team via this form in order to coordinate publication and release dates: https://forms.cloud.microsoft/e/eH4A8JVP6g

If you have any questions, please do not hesitate to contact the Editorial Office. Thank you for your contribution to EMBO Molecular Medicine.

Yours sincerely,
Zeljko Durdevic

Zeljko Durdevic
Senior Editor
EMBO Molecular Medicine

>>> Please note that it is EMBO Molecular Medicine policy for the transcript of the editorial process (containing referee reports and your response letter) to be published as an online supplement to each paper. If you do NOT want this, you will need to inform the Editorial Office via email immediately. More information is available here: https://link.springer.com/partners/embo-press/editorial-policies#Peer%20review

EMBO Press Author Checklist

Corresponding Author Name: Jolien Vandewalle
Journal Submitted to: EMBO Molecular Medicine
Manuscript Number: EMM-2026-23502

USEFUL LINKS FOR COMPLETING THIS FORM
The EMBO Journal - Author Guidelines
EMBO Reports - Author Guidelines
Molecular Systems Biology - Author Guidelines
EMBO Molecular Medicine - Author Guidelines

Reporting Checklist for Life Science Articles (updated January
This checklist is adapted from Materials Design Analysis Reporting (MDAR) Checklist for Authors. MDAR establishes a minimum set of requirements in transparent reporting in the life sciences (see Statement of Task: 10.31222/osf.io/9sm4x). Please follow the journal's guidelines in preparing your
Please note that a copy of this checklist will be published alongside your article.

Abridged guidelines for figures
1. Data
The data shown in figures should satisfy the following conditions:
- the data were obtained and processed according to the field's best practice and are presented to reflect the results of the experiments in an accurate and unbiased manner.
- ideally, figure panels should include only measurements that are directly comparable to each other and obtained with the same assay.
- plots include clearly labeled error bars for independent experiments and sample sizes. Unless justified, error bars should not be shown for technical
- if n<5, the individual data points from each experiment should be plotted. Any statistical test employed should be justified.
- Source Data should be included to report the data underlying figures according to the guidelines set out in the authorship guidelines on Data

2. Captions
Each figure caption should contain the following information, for each panel where they are relevant:
- a specification of the experimental system investigated (eg cell line, species name).
- the assay(s) and method(s) used to carry out the reported observations and measurements.
- an explicit mention of the biological and chemical entity(ies) that are being measured.
- an explicit mention of the biological and chemical entity(ies) that are altered/varied/perturbed in a controlled manner.
- the exact sample size (n) for each experimental group/condition, given as a number, not a range;
- a description of the sample collection allowing the reader to understand whether the samples represent technical or biological replicates (including how many animals, litters, cultures, etc.).
- a statement of how many times the experiment shown was independently replicated in the laboratory.
- definitions of statistical methods and measures:
  - common tests, such as t-test (please specify whether paired vs. unpaired), simple χ^2 tests, Wilcoxon and Mann-Whitney tests, can be unambiguously identified by name only, but more complex techniques should be described in the methods section;
  - are tests one-sided or two-sided?
  - are there adjustments for multiple comparisons?
  - exact statistical test results, e.g., P values = x but not P values < x;
  - definition of 'center values' as median or average;
  - definition of error bars as s.d. or s.e.m.

Please complete ALL of the questions below.
Select "Not Applicable" only when the requested information is not relevant for your study.

Materials

Newly Created Materials	Information included in the manuscript?	In which section is the information available? (Reagents and Tools Table, Materials and Methods, Figures, Data Availability Section)
New materials and reagents need to be available; do any restrictions apply?	Yes	Materials and Methods, Reagents and Tools Table

Antibodies	Information included in the manuscript?	In which section is the information available? (Reagents and Tools Table, Materials and Methods, Figures, Data Availability Section)
For **antibodies** provide the following information:<br>- Commercial antibodies: RRID (if possible) or supplier name, catalogue number and or/clone number<br>- Non-commercial: RRID or citation	Yes	Materials and Methods, Reagents and Tools Table

DNA and RNA sequences	Information included in the manuscript?	In which section is the information available? (Reagents and Tools Table, Materials and Methods, Figures, Data Availability Section)
**Short novel DNA or RNA including primers, probes**: provide the sequences.	Yes	Reagents and Tools Table

Cell materials	Information included in the manuscript?	In which section is the information available? (Reagents and Tools Table, Materials and Methods, Figures, Data Availability Section)
**Cell lines:** Provide species information, strain. Provide accession number in repository **OR** supplier name, catalog number, clone number, and/**OR** RRID.	Not Applicable	
**Primary cultures:** Provide species, strain, sex of origin, genetic modification status.	Not Applicable	
Report if the cell lines were recently **authenticated** (e.g., by STR profiling) and tested for mycoplasma contamination.	Not Applicable	

Experimental animals	Information included in the manuscript?	In which section is the information available? (Reagents and Tools Table, Materials and Methods, Figures, Data Availability Section)
**Laboratory animals or Model organisms:** Provide species, strain, sex, age, genetic modification status. Provide accession number in repository **OR** supplier name, catalog number, clone number, **OR** RRID.	Yes	Materials and Methods, Reagents and Tools Table
**Animal observed in or captured from the field:** Provide species, sex, and age where possible.	Not Applicable	
Please detail **housing and husbandry conditions**.	Yes	Materials and Methods

Plants and microbes	Information included in the manuscript?	In which section is the information available? (Reagents and Tools Table, Materials and Methods, Figures, Data Availability Section)
**Plants:** provide species and strain, ecotype and cultivar where relevant, unique accession number if available, and source (including location for collected wild specimens).	Not Applicable	
**Microbes:** provide species and strain, unique accession number if available, and source.	Yes	Materials and Methods

Human research participants	Information included in the manuscript?	In which section is the information available? (Reagents and Tools Table, Materials and Methods, Figures, Data Availability Section)
If collected and within the bounds of privacy constraints report on age, sex and gender or ethnicity for all study participants.	Not Applicable	

Core facilities	Information included in the manuscript?	In which section is the information available? (Reagents and Tools Table, Materials and Methods, Figures, Data Availability Section)
If your work benefited from core facilities, was their service mentioned in the acknowledgments section?	Yes	Acknowlegment

Design

Study protocol	Information included in the manuscript?	In which section is the information available? (Reagents and Tools Table, Materials and Methods, Figures, Data Availability Section)
If study protocol has been **pre-registered, provide DOI in the manuscript**. For clinical trials, provide the trial registration number **OR** cite DOI.	Not Applicable	
Report the **clinical trial registration number** (at ClinicalTrials.gov or equivalent), where applicable.	Not Applicable	

Laboratory protocol	Information included in the manuscript?	In which section is the information available? (Reagents and Tools Table, Materials and Methods, Figures, Data Availability Section)
Provide DOI OR other citation details if **external detailed step-by-step protocols** are available.	Not Applicable	

Experimental study design and statistics	Information included in the manuscript?	In which section is the information available? (Reagents and Tools Table, Materials and Methods, Figures, Data Availability Section)
Include a statement about **sample size** estimate even if no statistical methods were used.	Yes	Figures, Materials and Methods
Were any steps taken to minimize the effects of subjective bias when allocating animals/samples to treatment (e.g. **randomization procedure**)? If yes, have they been described?	Yes	Materials and Methods
Include a statement about **blinding** even if no blinding was done.	Yes	Materials and Methods
Describe **inclusion/exclusion criteria** if samples or animals were excluded from the analysis. Were the criteria pre-established?<br><br>If sample or data points were omitted from analysis, report if this was due to attrition or intentional exclusion and provide justification.	Yes	Materials and Methods
For every figure, are **statistical tests** justified as appropriate? Do the data meet the assumptions of the tests (e.g., normal distribution)? Describe any methods used to assess it. Is there an estimate of variation within each group of data? Is the variance similar between the groups that are being statistically compared?	Yes	Figures, Materials and Methods

Sample definition and in-laboratory replication	Information included in the manuscript?	In which section is the information available? (Reagents and Tools Table, Materials and Methods, Figures, Data Availability Section)
In the figure legends: state number of times the experiment was **replicated** in laboratory.	Yes	Figures
In the figure legends: define whether data describe **technical or biological replicates**.	Yes	Figures

Ethics

Ethics	Information included in the manuscript?	In which section is the information available? (Reagents and Tools Table, Materials and Methods, Figures, Data Availability Section)
Studies involving **human participants**: State details of **authority granting ethics approval** (IRB or equivalent committee(s), provide reference number for approval.	Not Applicable	
Studies involving **human participants**: Include a statement confirming that **informed consent** was obtained from all subjects and that the experiments conformed to the principles set out in the WMA Declaration of Helsinki and the Department of Health and Human Services Belmont Report.	Not Applicable	
Studies involving **human participants:** For publication of **patient photos**, include a statement confirming that consent to publish was obtained.	Not Applicable	
Studies involving experimental **animals**: State details of **authority granting ethics approval** (IRB or equivalent committee(s), provide reference number for approval. Include a statement of compliance with ethical regulations.	Yes	Materials and Methods
Studies involving **specimen and field samples:** State if relevant **permits** obtained, provide details of authority approving study; if none were required, explain why.	Not Applicable	

Dual Use Research of Concern (DURC)	Information included in the manuscript?	In which section is the information available? (Reagents and Tools Table, Materials and Methods, Figures, Data Availability Section)
Could your study fall under dual use research restrictions? Please check biosecurity documents and list of **select agents and toxins** (CDC): https://www.selectagents.gov/sat/list.htm	Not Applicable	
If you used a select agent, is the security level of the lab appropriate and reported in the manuscript?	Not Applicable	
If a study is subject to dual use research of concern regulations, is the name of the **authority granting approval and reference number** for the regulatory approval provided in the manuscript?	Not Applicable	

Reporting

The MDAR framework recommends adoption of discipline-specific guidelines, established and endorsed through community initiatives. Journals have their own policy about requiring specific guidelines and recommendations to complement MDAR.

Adherence to community standards	Information included in the manuscript?	In which section is the information available? (Reagents and Tools Table, Materials and Methods, Figures, Data Availability Section)
State if relevant guidelines or checklists (e.g., **ICMJE, MIBBI, ARRIVE, PRISMA**) have been followed or provided.	Yes	Materials and Methods
For **tumor marker prognostic studies,** we recommend that you follow the **REMARK** reporting guidelines (see link list at top right). See author guidelines, under 'Reporting Guidelines'. Please confirm you have followed these guidelines.	Not Applicable	
For **phase II and III randomized controlled trials**, please refer to the **CONSORT** flow diagram (see link list at top right) and submit the CONSORT checklist (see link list at top right) with your submission. See author guidelines, under 'Reporting Guidelines'. Please confirm you have submitted this list.	Not Applicable	

Data Availability

Data availability	Information included in the manuscript?	In which section is the information available? (Reagents and Tools Table, Materials and Methods, Figures, Data Availability Section)
Have **primary datasets** been deposited according to the journal's guidelines (see 'Data Deposition' section) and the respective accession numbers provided in the Data Availability Section?	Yes	Materials and Methods, Data Availability Section
Were **human clinical and genomic datasets** deposited in a public access-controlled repository in accordance to ethical obligations to the patients and to the applicable consent agreement?	Not Applicable	
Are **computational models** that are central and integral to a study available without restrictions in a machine-readable form? Were the relevant accession numbers or links provided?	Yes	Materials and Methods, Reagents and Tools Table
If publicly available data were reused, provide the respective **data citations in the reference list**.	Yes	Figures, Materials and Methods
