# Supplementary material for: RXRα suppression drives hepatic metabolic and immune dysfunction in sepsis
Source: EMBO Mol Med. 2026 Jul 16;18(8):3318–44. doi: 10.1038/s44321-026-00480-y (PMC13469149; doi:10.1038/s44321-026-00480-y)
Supplement: Supplementary file 2 [file 44321_2026_480_MOESM8_ESM.pdf]

Expanded View Figures

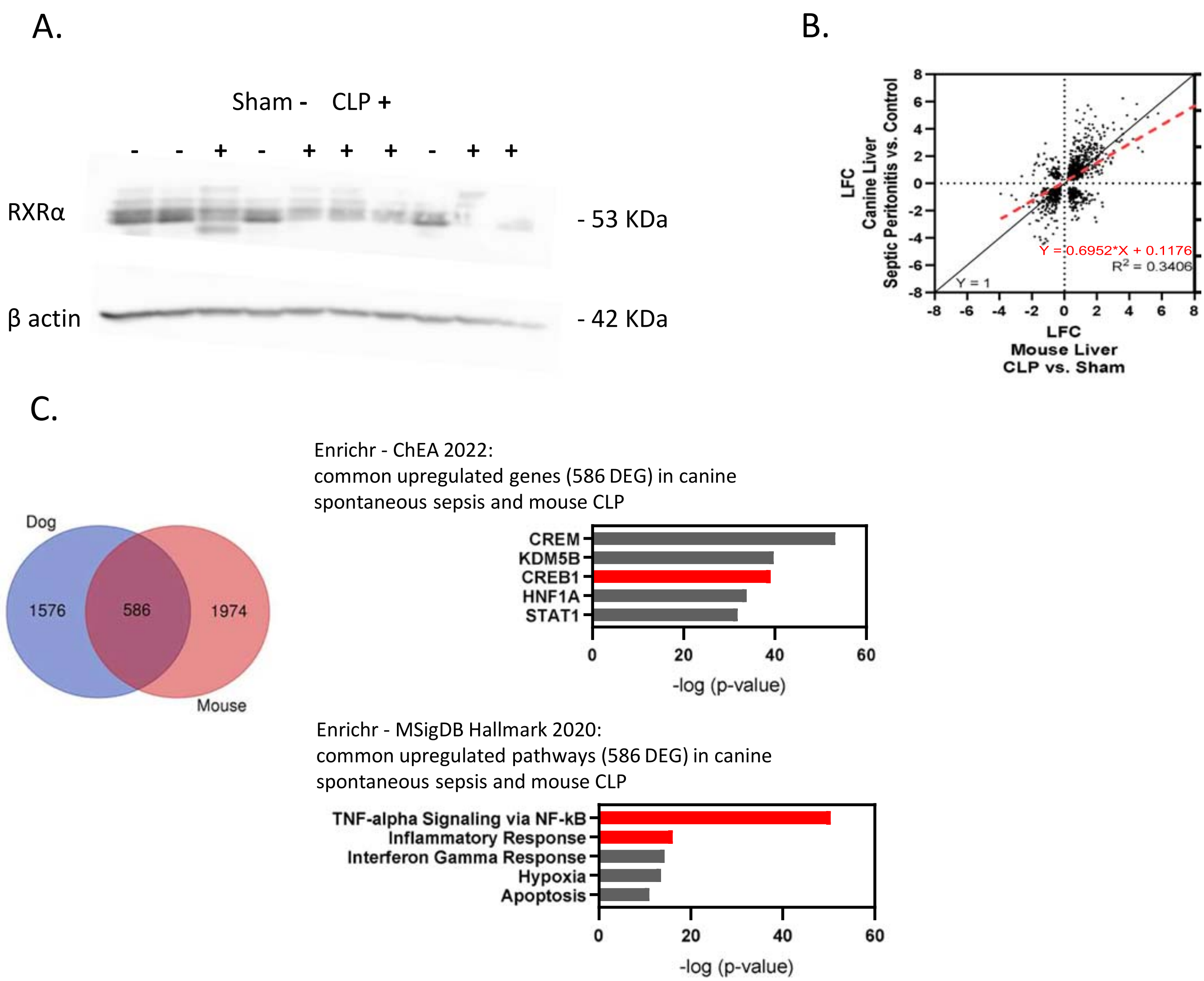

Figure EV1. Cross-species transcriptional conservation and hepatic RXRα protein dynamics.

(**A**) Western blot analysis of RXRα protein expression in WT liver homogenate 8 h after CLP ($n = 4$ sham; $n = 6$ CLP individual mice). (**B**) Correlation analysis of $\log_2$ fold-changes (LFC) between spontaneous canine sepsis and murine induced CLP (8 h) evaluated using linear regression and Pearson correlation analysis ($y = 0.6952x + 0.1176$; $R^2 = 0.3406$). (**C**) Venn diagram illustrating overlap of upregulated DEGs between septic dogs and mice (586 shared genes), with corresponding enrichment analysis (ChEA 2022, top; MSigDB Hallmark 2020, bottom). Pathways and regulators directly associated with the RXRα signaling axis are highlighted in red.

© The Author(s)

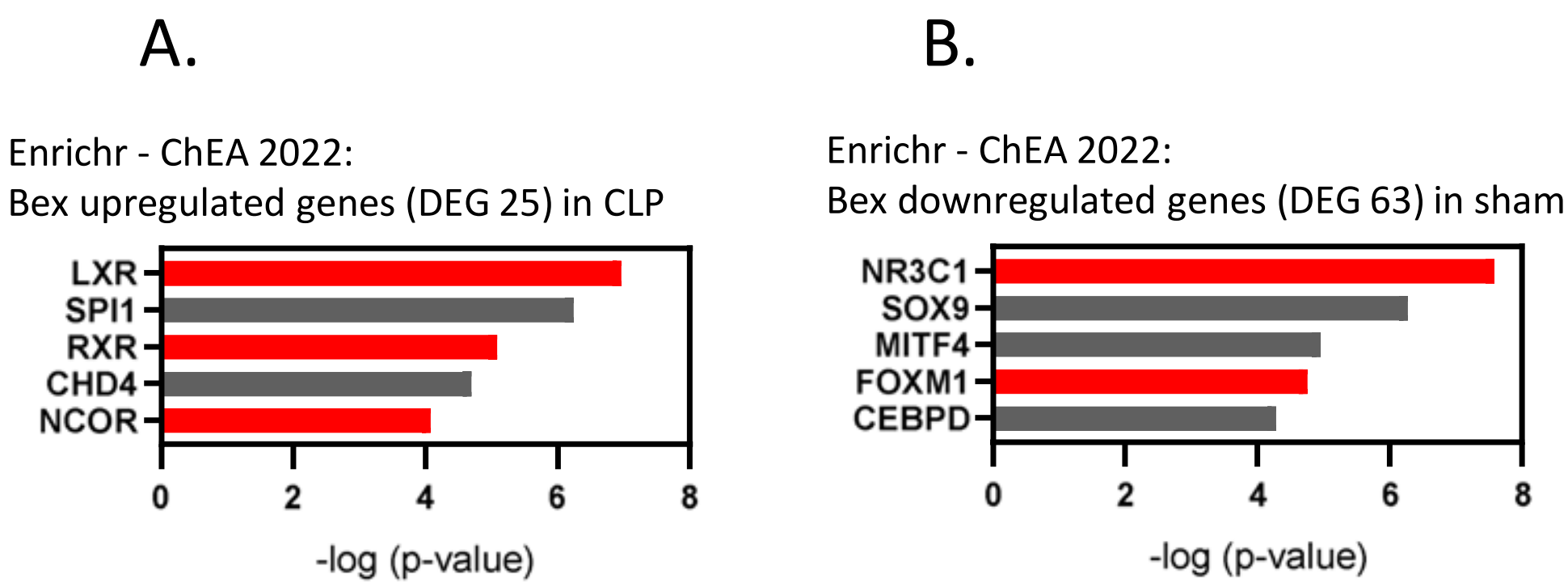

Figure EV2. Enrichment analysis of bexarotene treatment.

(**A**) Transcription factor (ChEA 2022) enrichment analysis of genes significantly upregulated by Bex in CLP mice compared to DMSO controls (adj. $p < 0.05$) ($n = 3$ individual mice per group). (**B**) Transcription factor (ChEA 2022) enrichment analysis of genes significantly downregulated by Bex in Sham mice compared to DMSO controls (adj. $p < 0.05$). Bars represent negative log10 *p* values. Pathways and regulators directly associated with the RXRα signaling axis are highlighted in red.

 © The Author(s)

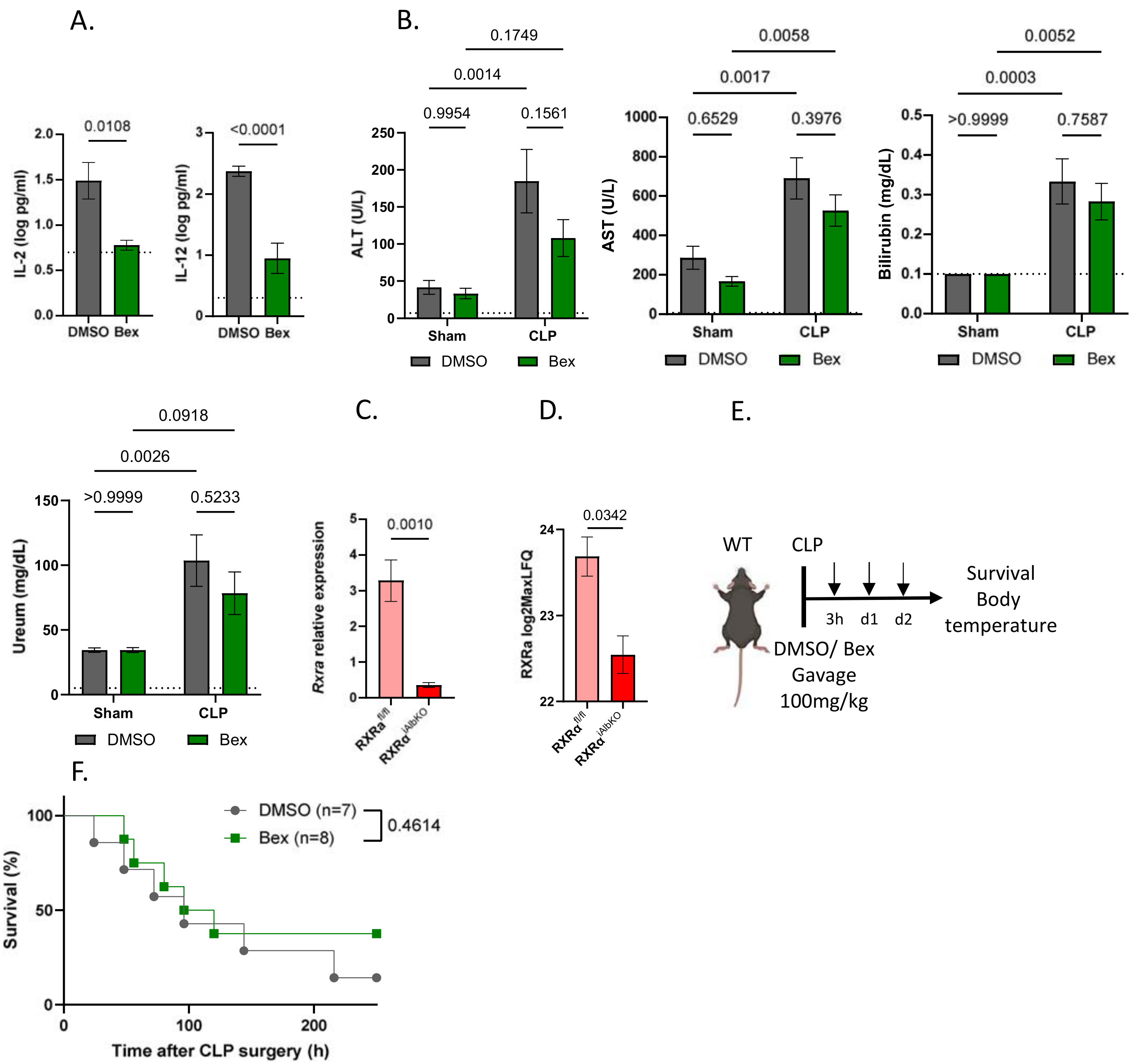

Figure EV3. Validation of hepatocyte-specific RXRα deletion and survival analysis of therapeutic bexarotene administration.

(**A**) Plasma IL-2 and IL-12 cytokine levels in DMSO and Bex-treated mice after CLP ($n = 10$ individual mice per group; data were log-transformed and analyzed using an unpaired Student's *t*-test). (**B**) Plasma organ damage parameters (alanine aminotransferase (ALT), aspartate aminotransferase (AST), bilirubin and ureum) in DMSO and Bex-treated mice ($n = 12$ individual mice per group; analyzed using an unpaired Student's *t*-test). Dashed lines indicate the limit of detection (LOD) for each respective biochemical assay. (**C**) Hepatic *Rxra* mRNA expression in $RXR\alpha^{fl/fl}$ and $RXR\alpha^{iAlbKO}$ mice was measured using primers specific for the deleted exon 4 ($RXR\alpha^{fl/fl}$ $n = 9$; $RXR\alpha^{iAlbKO}$ $n = 6$ individual mice; data were log-transformed and analyzed using an unpaired Student's *t*-test). (**D**) Hepatic RXRα protein abundance (MaxLFQ intensity) in $RXR\alpha^{fl/fl}$ and $RXR\alpha^{iAlbKO}$ livers determined by shotgun proteomics ($RXR\alpha^{fl/fl}$ $n = 7$; $RXR\alpha^{iAlbKO}$ $n = 8$ individual mice; analyzed using a *limma* moderated *t*-test). (**E**) Schematic representation of the therapeutic dosing regimen. Mice were subjected to CLP surgery and subsequently treated with DMSO or Bex (100 mg/kg, oral gavage) at 3, 24, and 48 h post-surgery. (**F**) Survival of septic mice following therapeutic administration of DMSO ($n = 7$) or Bex ($n = 8$ mice per group; analyzed using the log-rank (Mantel–Cox) test). Where applicable, data represent mean ± SD. Exact *p* values are indicated directly within the figure panels.

© The Author(s)

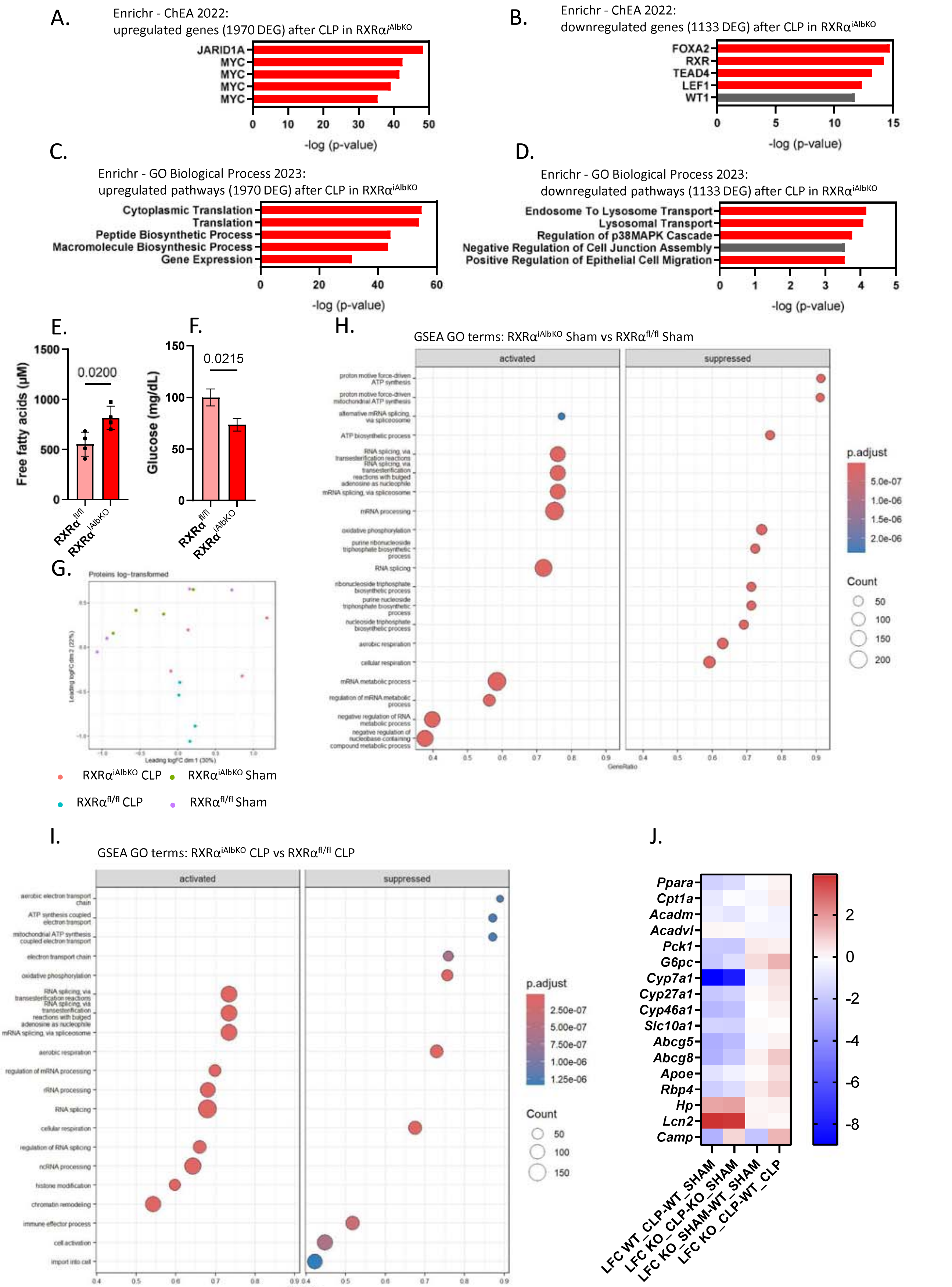
A.
Enrichr - ChEA 2022:
upregulated genes (1970 DEG) after CLP in RXRα^iAlbKO
JARID1A
MYC
MYC
MYC
MYC
0 10 20 30 40 50
-log (p-value)
B.
Enrichr - ChEA 2022:
downregulated genes (1133 DEG) after CLP in RXRα^iAlbKO
FOXA2
RXR
TEAD4
LEF1
WT1
0 5 10 15
-log (p-value)
C.
Enrichr - GO Biological Process 2023:
upregulated pathways (1970 DEG) after CLP in RXRα^iAlbKO
Cytoplasmic Translation
Translation
Peptide Biosynthetic Process
Macromolecule Biosynthesis Process
Gene Expression
0 20 40 60
-log (p-value)
D.
Enrichr - GO Biological Process 2023:
downregulated pathways (1133 DEG) after CLP in RXRα^iAlbKO
Endosome To Lysosome Transport
Lysosomal Transport
Regulation of p38MAPK Cascade
Negative Regulation of Cell Junction Assembly
Positive Regulation of Epithelial Cell Migration
0 1 2 3 4 5
-log (p-value)
E.
Free fatty acids (μM)
0.0200
RXRα^fl/fl
RXRα^iAlbKO
F.
Glucose (mg/dL)
0.0215
RXRα^fl/fl
RXRα^iAlbKO
G.
Proteins log-transformed
Leading logFC dim 1 (30%)
Leading logFC dim 2 (22%)
RXRα^iAlbKO CLP
RXRα^iAlbKO Sham
RXRα^fl/fl CLP
RXRα^fl/fl Sham
H.
GSEA GO terms: RXRα^iAlbKO Sham vs RXRα^fl/fl Sham
activated
suppressed
proton motive force-driven ATP synthesis
proton motive force-driven mitochondrial ATP synthesis
alternative mRNA splicing, via spliceosome
ATP biosynthetic process
RNA splicing, via transesterification reactions
RNA splicing, via transesterification reactions with bulged adenosine as nucleophile
mRNA splicing, via spliceosome
mRNA processing
oxidative phosphorylation
purine ribonucleoside triphosphate biosynthetic process
RNA splicing
ribonucleoside triphosphate biosynthetic process
purine nucleoside triphosphate biosynthetic process
nucleoside triphosphate biosynthetic process
aerobic respiration
cellular respiration
mRNA metabolic process
regulation of mRNA metabolic process
negative regulation of RNA metabolic process
negative regulation of nucleobase-containing compound metabolic process
GeneRatio
p.adjust
5.0e-07
1.0e-06
1.5e-06
2.0e-06
Count
50
100
150
200
I.
GSEA GO terms: RXRα^iAlbKO CLP vs RXRα^fl/fl CLP
activated
suppressed
aerobic electron transport chain
ATP synthesis coupled electron transport
mitochondrial ATP synthesis coupled electron transport
electron transport chain
oxidative phosphorylation
RNA splicing, via transesterification reactions
RNA splicing, via transesterification reactions with bulged adenosine as nucleophile
mRNA splicing, via spliceosome
aerobic respiration
regulation of mRNA processing
rRNA processing
RNA splicing
cellular respiration
regulation of RNA splicing
ncRNA processing
histone modification
chromatin remodeling
immune effector process
cell activation
import into cell
GeneRatio
p.adjust
2.50e-07
5.00e-07
7.50e-07
1.00e-06
1.25e-06
Count
50
100
150
J.
Ppara
Cpt1a
Acadm
Acadvl
Pck1
G6pc
Cyp7a1
Cyp27a1
Cyp46a1
Slc10a1
Abcg5
Abcg8
Apoe
Rbp4
Hp
Lcn2
Camp
2
0
-2
-4
-6
-8
LFC WT_CLP-WT_SHAM
LFC KO_CLP-KO_SHAM
LFC KO_SHAM-WT_SHAM
LFC KO_CLP-WT_CLP

 © The Author(s)

Figure EV4. Transcriptomic and proteomic landscape of the septic liver in the absence of hepatocyte RXRα.

(**A–D**) Transcription factor (ChEA 2022) and pathway (GO Biological Process 2023) enrichment analysis of (**A**, **C**) upregulated and (**B**, **D**) downregulated DEGs (adj. $p < 0.05$) in $RXR\alpha^{iAlbKO}$ vs. $RXR\alpha^{fl/fl}$ livers following CLP ($n = 3$ individual mice per group). Pathways and regulators directly associated with the RXRα signaling axis are highlighted in red. (**E**) Plasma free fatty acids (FFAs) concentration 24 h post-CLP ($n = 4$ individual mice per group; analyzed using an unpaired Student's *t*-test). (**F**) Blood glucose levels 24 h post-CLP ($RXR\alpha^{fl/fl}$ $n = 8$; $RXR\alpha^{iAlbKO}$ $n = 11$ individual mice; analyzed using an unpaired Student's *t*-test). (**G**) PCA plot of the nuclear-enriched proteome from $RXR\alpha^{fl/fl}$ and $RXR\alpha^{iAlbKO}$ livers 24 h after sham or CLP surgery. (**H**) Gene set enrichment analysis (GSEA) of the proteome in Sham $RXR\alpha^{iAlbKO}$ vs. $RXR\alpha^{fl/fl}$ livers. (**I**) GSEA of the proteome in CLP $RXR\alpha^{iAlbKO}$ vs. $RXR\alpha^{fl/fl}$ livers. Dot size in GSEA represents gene count, and color indicates the adjusted p-value. (**J**) Heatmap of LFC for representative candidate genes involved in master regulation of fatty acid oxidation, gluconeogenesis, oxysterol and bile acid synthesis, lipid and retinoid transport, and antimicrobial defense across the specified experimental comparisons. Where applicable, data represent mean ± SD. Exact *p* values are indicated directly within the figure panels.

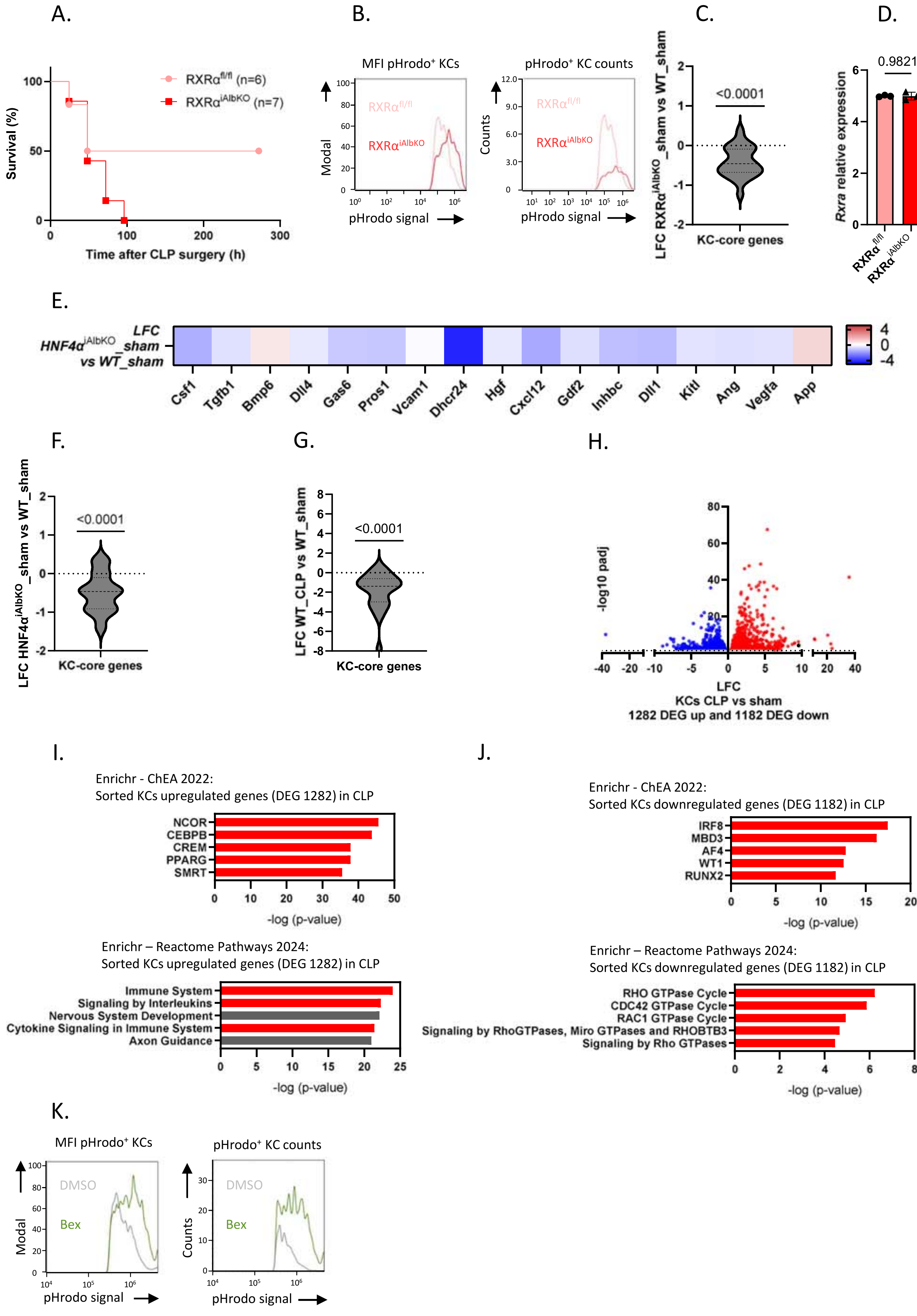
A.
B.
C.
D.
Survival (%)
Time after CLP surgery (h)
RXRα fl/fl (n=6)
RXRα iAlbKO (n=7)
MFI pHrodo+ KCs
pHrodo+ KC counts
Modal
Counts
pHrodo signal
0.9821
<0.0001
KC-core genes
LFC RXRα iAlbKO_sham vs WT_sham
Rxra relative expression
E.
LFC HNF4α iAlbKO_sham vs WT_sham
Csf1
Tgfb1
Bmp6
Dll4
Gas6
Pros1
Vcam1
Dhcr24
Hgf
Cxcl12
Gdf2
Inhbc
Dll1
Kitl
Ang
Vegfa
App
F.
G.
H.
LFC WT_CLP vs WT_sham
-log10 padj
LFC
KCs CLP vs sham
1282 DEG up and 1182 DEG down
I.
J.
Enrichr - ChEA 2022:
Sorted KCs upregulated genes (DEG 1282) in CLP
NCOR
CEBPB
CREM
PPARG
SMRT
-log (p-value)
Sorted KCs downregulated genes (DEG 1182) in CLP
IRF8
MBD3
AF4
WT1
RUNX2
Enrichr – Reactome Pathways 2024:
Immune System
Signaling by Interleukins
Nervous System Development
Cytokine Signaling in Immune System
Axon Guidance
RHO GTPase Cycle
CDC42 GTPase Cycle
RAC1 GTPase Cycle
Signaling by RhoGTPases, Miro GTPases and RHOBTB3
Signaling by Rho GTPases
K.
DMSO
Bex

◀ **Figure EV5. Transcriptomic and functional characterization of Kupffer cells in RXRα deficiency and sepsis.**

(**A**) Survival curve corresponding to the time-course analysis of bacterial dissemination (Fig. 5C) (RXRα$^{fl/fl}$ $n = 6$ and RXRα^{iAlbKO} $n = 7$ individual mice; analyzed using the log-rank (Mantel–Cox) test). (**B**) Representative spectral flow cytometry plots demonstrating in vivo pHrodo-labeled *E. coli* uptake (MFI) in naïve RXRα$^{fl/fl}$ and RXRα^{iAlbKO} mice. (**C**) Log_2 fold-change (LFC) of the KC-core gene signature in bulk liver RNA-seq from sham RXRα^{iAlbKO} mice compared to littermate controls ($n = 3$ independent sequencing replicates per group; analyzed using a one-sample *t*-test against a theoretical LFC mean of 0). (**D**) Relative mRNA expression of *Rxra* in FACS-sorted KCs using deleted exon-specific primers ($n = 3$ independent biological replicates per group; data were log-transformed and analyzed using an unpaired Student's *t*-test). (**E**) LFC of essential KC niche factors in bulk liver RNA-seq from sham HNF4α^{iAlbKO} mice compared to littermate controls ($n = 4$ independent sequencing replicates per group). (**F**) LFC of the KC-core gene signature in bulk liver RNA-seq from sham HNF4α^{iAlbKO} mice compared to littermate controls ($n = 4$ independent sequencing replicates per group; analyzed using a one-sample *t*-test against a theoretical LFC mean of 0). (**G**) LFC of the KC-core gene signature in bulk liver RNA-seq from WT mice 8 h post-CLP compared to sham ($n = 3$ independent sequencing replicates per group; analyzed using a one-sample *t*-test against a theoretical LFC mean of 0). (**H**) Volcano plot displaying differentially expressed genes (DEGs; adj. $p < 0.05$) in FACS-sorted KCs from WT mice following CLP compared to sham surgery. (**I**) Pathway enrichment analysis of significantly upregulated genes (adj. $p < 0.05$) in FACS-sorted KCs following CLP. (**J**) Pathway enrichment analysis of significantly downregulated genes (adj. $p < 0.05$) in FACS-sorted KCs following CLP. (**K**) Representative spectral flow cytometry plots demonstrating pHrodo-labeled *E. coli* uptake (MFI) in WT mice subjected to CLP and treated prophylactically with DMSO or Bexarotene. Where applicable, data represent mean ± SD. Exact *p* values are indicated directly within the figure panels.
